# Quest for an Efficient 2-in-1 MOF-Based Catalytic System for Cycloaddition of CO_2_ to Epoxides under Mild Conditions

**DOI:** 10.1021/acsami.0c20437

**Published:** 2021-02-09

**Authors:** Marzena Pander, Mateusz Janeta, Wojciech Bury

**Affiliations:** Faculty of Chemistry, University of Wrocław, 14 F. Joliot-Curie, 50-383 Wrocław, Poland

**Keywords:** metal−organic framework, postsynthetic functionalization, carbon dioxide fixation, cycloaddition, heterogeneous catalysis

## Abstract

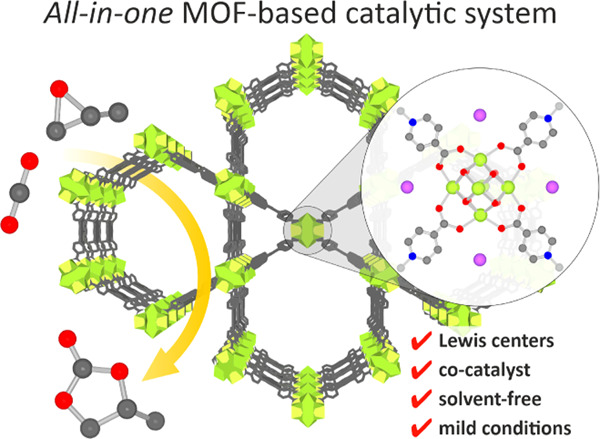

We have devised a straightforward tandem postsynthetic modification strategy for Zr-based metal–organic framework (MOF) materials, which resulted in a series of well-defined 2-in-1 heterogeneous catalysts, **cat1**–**cat8**, exhibiting high catalytic activity in the synthesis of cyclic carbonates under solvent-free and co-catalyst-free conditions. The materials feature precisely located co-catalyst moieties decorating the metal nodes throughout the bulk of the MOF and yield cyclic carbonates with up to 99% efficiency at room temperature. We use diffuse reflectance infrared Fourier transform (DRIFT) and solid-state nuclear magnetic resonance (NMR) measurements to elucidate the role of each component in this model catalytic reaction. Establishing a method to precisely control the co-catalyst loading allowed us to observe the cooperativity between Lewis acid sites and the co-catalyst in the 2-in-1 heterogeneous system.

## Introduction

In recent years, metal–organic frameworks (MOFs) have emerged as excellent candidates for heterogeneous catalysis, meeting some of the still-growing demands required in novel catalytic systems. Their activity and selectivity have already been tested in numerous catalytic reactions, often proving MOFs to be a superior alternative to many conventional catalysts.^1−5^ The unique possibility of their further postsynthetic modification offers a convenient way of introducing new functionalities into a system,^6,7^ for example, by direct functionalization of metal nodes.^8^ This approach is of high relevance in reactions where multiple chemical components are required for the catalytic cycle (e.g., catalytic cycloaddition of CO_2_ to reactive substrates).^9^

The reaction of CO_2_ with epoxides is an example where MOFs have been found to be especially efficient catalytic systems.^10−12^ However, in the materials developed to date, the activation of CO_2_ typically requires elevated temperatures (>100 °C) and pressures.^13,14^ More recently, MOF-based catalysts active at ambient conditions, i.e., room temperature and 1 atm of carbon dioxide, have been developed.^15−18^ In this context, zirconium-based MOFs (Zr-MOFs) have been proven to be active heterogeneous catalysts in various reactions, including cycloaddition of CO_2_ to epoxides in the presence of an external co-catalyst.^19^ Zr-MOFs represent a particularly interesting class of materials due to their remarkable thermal and chemical stabilities.^20−22^ To date, a number of design strategies have been presented based on the accessibility of Lewis acid sites^23,24^ and the connectivity of Zr_6_-nodes in microporous^25^ or hierarchically mesoporous MOFs.^26^ For example, Lyu et al. have compared the activity of metal Lewis acid sites in 8-connected M_6_^IV^-oxo nodes,^27^ which revealed the importance of terminal water molecules in the reactivity of the M_6_ clusters during the catalytic cycle.

In the abovementioned examples of the catalytic synthesis of cyclic carbonates, the presence of an additional co-catalyst (usually tetrabutylammonium bromide or iodide are used, TBAB and TBAI, respectively) was required.^10^ The use of an external co-catalyst brings about challenges related to diffusion of reactants into the pores of the catalytic system. First, the amount of the co-catalyst used by different researchers varies considerably, ranging from 0.3 to 10 mol %.^17,28^ Second, the spatial location of the catalytic process raises fundamental questions, namely, whether it takes place on the surface of the crystal or inside the porous network.^17^ Up till now, only a handful of examples have shown MOF-based materials that work without the presence of an additional co-catalyst.^29,30^ The introduction of nucleophilic anions (Br^–^ or I^–^) into the MOF structure is often ensured by de novo or postsynthetic incorporation of linkers containing quaternary ammonium,^31^ imidazolium,^32−36^ and pyridinium^37^ groups. Other catalyst design strategies are based on entrapping ionic polymers inside MOF pores.^38,39^ The main concern in these systems that needs to be addressed is the high probability of uneven distribution and accessibility of the nucleophilic anions in the bulk of the MOF. The rational design of a tailored catalytic system requires an in-depth analysis of the co-catalyst accessibility.

**Figure 1:**
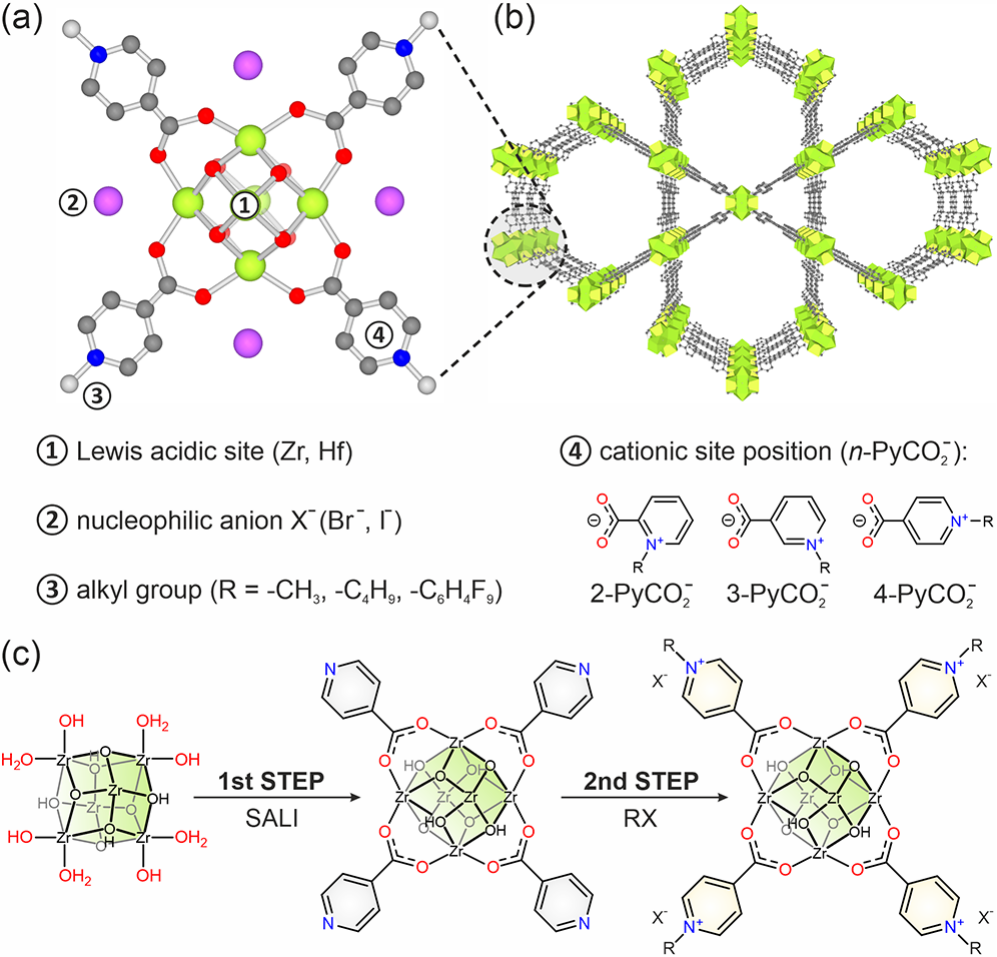
Design of a 2-in-1 MOF-based catalytic system based on the **NU-1000** platform: (a) functionalization of inorganic nodes; color code: C (gray), O (red), N (blue), Zr (light green), Br or I (violet), and R group (light gray); (b) the structure of **NU-1000**; and (c) the schematic representation of tandem postsynthetic functionalization of the 8-connected Zr-nodes.

Here, we report a facile strategy of tandem postsynthetic modifications of metal nodes in selected mesoporous MOFs that converts a nonactive porous material into a very efficient 2-in-1 catalytic system in the cycloaddition of carbon dioxide to epoxides under ambient conditions (Figure 1). As a platform for this study, we selected well-established MOF platforms, namely, Zr- and Hf-based **NU-1000** frameworks, consisting of 8-connected Zr_6_-nodes and TBAPy^4–^ (1,3,6,8-tetrakis(*p*-benzoate)pyrene) linkers that feature high chemical stability, hierarchical porosity, and a unique tendency to undergo various postsynthetic modifications.^15,40,41^ In our synthetic approach, we focused our experimental endeavors on four ways of viable functionalization of the Zr-MOF nodes by (1) introduction of various metal centers (Zr vs Hf), (2) modification of the nucleophilic counterion (Br^–^ vs I^–^), (3) incorporation of different pyridinium cationic moieties (pyridine-*n*-carboxylic acids, where *n* = 2–4), and (4) the influence of aliphatic or fluorinated alkyl substituents. As a result, we prepared and examined a broad group of **NU-1000** derivatives, which can be conveniently obtained by a well-developed solvent-assisted ligand incorporation (SALI) protocol and further extended by subsequent alkylation with selected haloalkyl reagents. This library of well-parameterized catalysts allowed us to perform a more detailed analysis of factors governing the structure–reactivity relationship for this widely explored catalytic model system.

## Experimental Section

### Synthesis of **NU-1000(M)** (M = Zr, Hf)

The materials were synthesized according to the procedure described by Islamoglu et al., where, in addition to benzoic acid, trifluoroacetic acid (TFA) was used as a co-modulator.^42^ The synthetic procedure for **NU-1000(Hf)** was analogous to that of **NU-1000(Zr)** (with TFA as a co-modulator; see Section S.3.1).^15^ The synthesized **NU-1000(M)** materials were characterized by powder X-ray diffraction (PXRD), proton nuclear magnetic resonance (^1^H NMR), diffuse reflectance infrared Fourier transform spectroscopy (DRIFTS), thermogravimetric analysis (TGA), and N_2_ sorption measurements.

### SALI Reaction with Pyridinecarboxylic Acids^43^

Briefly, 20 mg of **NU-1000(Zr)** (0.009 mmol) or 25 mg of **NU-1000(Hf)** (0.009 mmol) was soaked in 5 mL of 0.03 M solution of pyridine-4-carboxylic acid (**4-PyCOOH**) in EtOH at 60 °C for 24 h. After this time duration, the supernatant was removed and the solid was soaked in 15 mL of a fresh portion of ethanol and incubated at 60 °C for the next 24 h to remove the unbound ligand. The obtained material was washed three times with ethanol and dried at 60 °C. The same procedure was applied in the case of **NU-1000(Zr)** for other isomers of the introduced ligands, namely, pyridine-2-carboxylic acid (**2-PyCOOH**) and pyridine-3-carboxylic acid (**3-PyCOOH**). The number of incorporated molecules of pyridinecarboxylic acids was determined by ^1^H NMR analysis of the samples digested in D_2_SO_4_/dimethyl sulfoxide (DMSO)-*d*_6_ mixture. The obtained materials were characterized by PXRD, DRIFTS, TGA, and N_2_ sorption measurements.

### Alkylation of **SALI-*****n*****-Py(M)** (*n* = 2–4, M = Zr, Hf) with Selected Haloalkanes

Initially, the alkylation reaction of the pyridine moiety in **NU-1000(Zr)** was performed in toluene at 100 °C based on a literature procedure.^44^ The synthetic approach was then optimized in acetonitrile at lower temperatures. In this case, 30 mg of **SALI-*****n-*****Py(Zr)** (*n* = 2–4, 0.012 mmol) or 30 mg of **SALI-4-Py(Hf)** (0.011 mmol) was soaked in 2 mL of acetonitrile in a microwave reaction vial (Biotage). Then, the selected alkyl halide (RX) was added to the solution, and the vial was sealed and placed in an Eppendorf ThermoMixer (500 rpm, 60 or 80 °C) for 24 or 48 h (for reaction details, see Section S3.3). Next, the obtained solid was washed three times with acetonitrile and dried at 80 °C. The yield of pyridine moiety alkylation was determined by ^1^H NMR analysis of the digested samples.

### Catalytic Reactions of Carbon Dioxide with Epoxides

In a typical reaction, a 5 mL vial, equipped with a small magnetic stirring bar, was charged with the selected epoxide (0.087 mmol), 1 mol % prepared MOF-based catalyst, and 30 mg of solid CO_2_ (0.682 mmol). The vial was tightly sealed, and the reaction mixture was stirred at 80 °C for 4 h (the CO_2_ pressure in the vial was approx. 4 bar). After this time duration, the vial was cooled in a liquid nitrogen bath and excess CO_2_ was slowly released. The vial was weighed before and after the performed reaction to ensure the tightness of the system. Then, 5 μL of mesitylene (internal standard, 4.32 mg, 0.036 mmol) was added to the crude reaction mixture, and the catalyst was separated by washing twice with 0.4 mL of CDCl_3_ (6000 rpm, 2 min). For the recycling experiment, the recovered catalyst was washed thrice with acetone, dried under vacuum, and reused for the next cycle.

## Results and Discussion

### Design, Synthesis, and Characterization of 2-in-1 Catalytic Systems

Bearing in mind our design criteria to construct an effective catalyst simultaneously composed of active metal centers and nucleophilic anions, as a platform for our studies, we selected the well-known **NU-1000** material because its micro/mesoporous framework offers large room for further functionalization. The zirconium-based **NU-1000(Zr)** and its hafnium analogue, **NU-1000(Hf)**, were obtained according to literature procedures (see the Supporting Information for details). It has been shown previously that Zr-MOFs easily undergo functionalization of inorganic nodes by incorporation of nonstructural ligands.^44^ Therefore, in our approach, we decided to use postsynthetic modification as a convenient way to precisely control the distribution of introduced functionalities inside the porous framework. This allowed us to investigate the influence of the co-catalyst moieties introduced in proximity to the inorganic node. In the first step, the incorporation of *ortho-*, *meta-*, and *para-*pyridinecarboxylic acids into the framework of **NU-1000(M)** was performed (step 1 in Figure 1). In the second step, pyridine moieties were alkylated with various haloalkanes, namely, methyl iodide (CH_3_I), butyl iodide (C_4_H_9_I), butyl bromide (C_4_H_9_Br), and 1*H*,1*H*,2*H*,2*H*-perfluorohexyl iodide (C_6_H_4_F_9_I) (Figure 1b). The selected alkylating agents differ in alkyl chain length, polarity, or type of the halogen and hence might affect the properties of the pore and metal nodes. The effectiveness of each step of the postsynthetic functionalization of metal nodes in **NU-1000(M)** and hence the chemical composition of organic constituents in **cat1**–**cat8** were determined by spectral analysis in solution and the solid state (Sections S4.2 and S4.3). The yields of the SALI reaction and subsequent alkylation of pyridine moieties were calculated by integrating proton signals of introduced nonstructural ligands against those of the TBAPy^4–^ linker. The determined compositions of **cat1**–**cat8**, including their sorption properties, are summarized in Table 1. In most cases, the yield of the performed postsynthetic modification was close to the theoretical maximum of four nonstructural ligands per 8-connected M_6_-node (M = Zr, Hf).

**Table 1:** Structural Compositions and Sorption Data of the MOF-Based Catalysts by Tandem PSM of M_6_-Nodes (M = Zr, Hf)

	reagents for tandem PSM		composition of the catalyst				
catalyst (M = Zr, Hf)	*n*-PyCOOH (step 1)	RX (step 2)	*n*-PyCO_2_^–^/node^a^	RX-*n*-PyCO_2_^–^/node^a^	*S*_BET_ (m^2^/g)	*V*_pore_ (cm^3^/g)^b^	*Q*_st_ (kJ/mol)^c^
**NU-1000(Zr)**	n/a	n/a	n/a	n/a	2062	1.42	
**NU-1000(Hf)**	n/a	n/a	n/a	n/a	1783	1.21	
**SALI-4-Py(Zr)**	4-PyCOOH	n/a	4	n/a	1920	1.09	
**SALI-4-Py(Hf)**	4-PyCOOH	n/a	4	n/a	1797	0.97	
**SALI-3-Py(Zr)**	3-PyCOOH	n/a	4	n/a	2061	1.17	
**SALI-2-Py(Zr)**	2-PyCOOH	n/a	4	n/a	1852	1.06	
**cat1** (Zr)	4-PyCOOH	CH_3_I	0	3.8	1201	0.72	28.5–19.6
**cat2** (Zr)	3-PyCOOH	CH_3_I	0	4	1157	0.67	
**cat3** (Zr)	2-PyCOOH	CH_3_I	1.5	2	1523	0.93	
**cat4** (Zr)	4-PyCOOH	C_4_H_9_I	0	3.8	1205	0.73	20.6–19.3
**cat5** (Zr)	4-PyCOOH	C_4_H_9_Br	0	3.7	1510	0.94	27.6–24.4
**cat6** (Zr)	4-PyCOOH	C_6_H_4_F_9_I	1	2.5	1339	0.75	22.3–21.7
**cat7** (Hf)	4-PyCOOH	C_4_H_9_I	0	4	1142	0.67	21.8–18.6
**cat8** (Hf)	4-PyCOOH	C_4_H_9_Br	0	4	1097	0.69	24.9–22.9

a Based on ^1^H NMR analysis of respective samples digested in the D_2_SO_4_/DMSO-*d*_6_ mixture.

b Total pore volume calculated from single-point nitrogen uptake at *p*/*p*_0_ = 0.9.

c Based on the dual-site Langmuir (DSL) model.

Among selected alkylating agents, methyl iodide was the most reactive, for which the reaction easily occurred under mild conditions (60 °C, less than 24 h). Similar reactivity was observed for butyl iodide, which may be regarded as a more convenient and less toxic alkylating reagent than CH_3_I. When butyl bromide was used as an alkylating agent, more demanding conditions were required, and full conversion was observed after a longer reaction time (80 °C, 48 h). The lower yield (>50%) of the alkylation reaction was observed in the case of **cat6**, where the longer chain 1*H*,1*H*,2*H*,2*H*-perfluorohexyl iodide was used. The complete methylation of pyridine *ortho-*isomer in **cat3** was not achieved even when reactions were performed at higher temperatures and for longer durations.^45^

**Figure 2:**
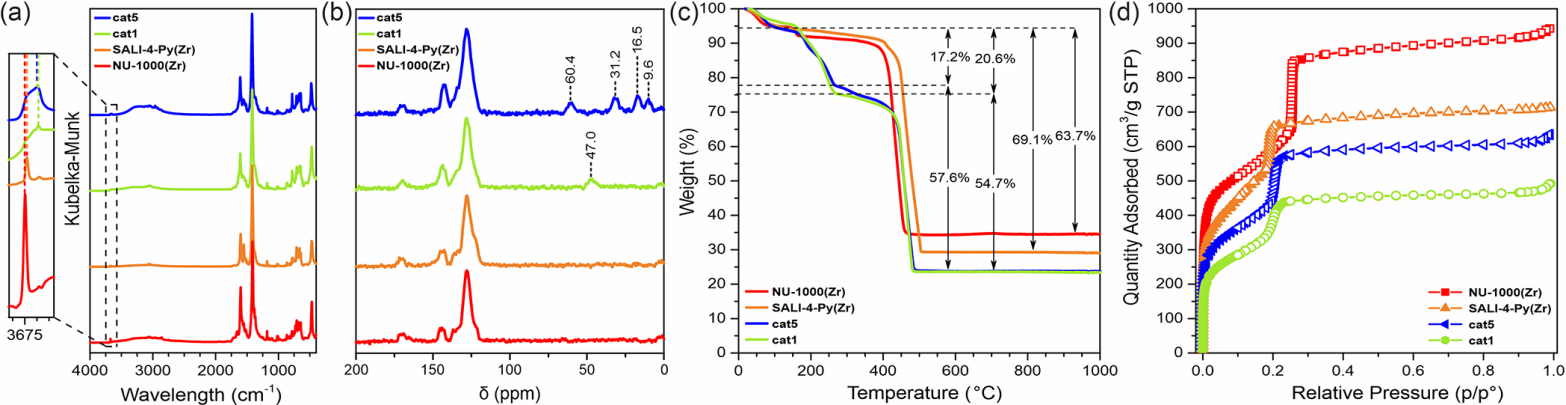
Structural characterization of representative 2-in-1 catalysts: (a) DRIFT spectra of the representative samples, (b) ^13^C CP-MAS spectra of examined materials, (c) TGA profiles under oxidative conditions (O_2_/N_2_ = 20/80); the corresponding weight loss is indicated with arrows, and (d) N_2_ sorption isotherms at 77 K; filled symbols, adsorption and open symbols, desorption.

To gain additional insight into the framework composition in the solid state, we resorted to ^13^C cross-polarization magic angle spinning (CP-MAS) NMR spectroscopy (Figure 2b). In the ^13^C CP-MAS spectrum of **NU-1000(Zr)**, two main bands at 143 and 128 ppm, from overlapping signals of aromatic carbon atoms of the TBAPy^4–^ linker, and a signal at 170 ppm that can be attributed to the carbon atom in the carboxylate group of the linker are present.^46^ In the ^13^C CP-MAS spectra of **cat1** and **cat5** (Figure 2b), a new set of signals is present in the aliphatic region; the one at 47 ppm was assigned to the carbon atom of the −CH_3_ group of the methylated pyridinium cation in **cat1**, and the others at 60, 32, 16, and 10 ppm were assigned to carbon atoms of the −C_4_H_9_ group in **cat5**. These signals confirm the presence of permanently attached alkyl groups in the **NU-1000** framework. The crystallinity and morphology of all of the materials during each step of the postsynthetic functionalization were maintained as confirmed by the PXRD data (Figures S1 and S2) and scanning electron microscopy (SEM) images (Section S4.7). Additionally, energy-dispersive X-ray spectroscopy (EDS) of **cat1**–**cat8** further confirmed the presence of introduced halogen atoms (F, Br, or I) in the **NU-1000(M)** structures (Section S4.7). The thermal stability of the obtained catalysts was examined using thermogravimetric analysis (TGA). In samples of **cat1**–**cat8** we observed that, while the MOF framework itself remains stable up to 300 °C, an additional significant weight loss is evident at lower temperatures (150–300 °C), which corresponds to the loss of the haloalkane molecules introduced in the MOF structure during the second step of functionalization (Section S4.6).

### Sorption Studies

To characterize the porosities of **cat1**–**cat8**, nitrogen adsorption–desorption isotherms (at 77 K) were collected, and the results are summarized in Table 1. For all samples, type IV nitrogen sorption isotherms were observed, which indicate the presence of both micropores and mesopores in the structure (Figures 2d and S24–S26a). In general, the systematic decrease of total pore volumes and Brunauer–Emmett–Teller (BET) specific surface areas was observed after each step of functionalization, which agrees well with previous studies reported for **NU-1000** and its SALI derivatives.^44^ A systematic decrease of the mesopore size (from ∼30 to ∼25 Å) in **NU-1000(M)** after incorporation of pyridinecarboxylic acids was indicated in the density functional theory (DFT)-calculated pore size distributions (PSDs, Figures S24–S26b), similar to **NU-1000(Zr)** derivatives functionalized with various *para*-substituted benzoic acids.^47^ Subsequent alkylation of pyridine moieties in MOFs further decreases the porosities of **cat1**–**cat8**, preserving, however, its hierarchical composition with micro- and mesopores in the framework (as shown in the corresponding PSDs, Figures S24–S26b). Using iodoalkanes as alkylating agents resulted in similar BET surface areas and isotherm traces, suggesting that the main influence on the decrease of the pore volume was the molar mass of the introduced anion. Nevertheless, for all materials (**cat1**–**cat8**), sufficient pore space has been preserved for the catalytic reaction.

Due to the use of carbon dioxide as one of the reagents in catalytic reactions, we first studied its interactions with the surfaces of **cat1**–**cat8** (see Section S4.6), using CO_2_ sorption. Then, the analysis of the enthalpy of carbon dioxide adsorption was performed. The isosteric heats of CO_2_ adsorption (*Q*_st_) were calculated from CO_2_ isotherms of **cat1** and **cat4**–**cat8**, measured in the temperature range of 273–293 K. The dual-site Langmuir (DSL) model for fitting the isotherms and the Clausius–Clapeyron equation for *Q*_st_ calculation were used (Figures S27–S33). In general, the calculated *Q*_st_ values for **cat1** and **cat4**–**cat8** are higher than those of nonfunctionalized **NU-1000(Zr)** (17 kJ/mol).^48^ Interestingly, the highest *Q*_st_ values (27.6–24.4 kJ/mol) were observed for **cat5** functionalized with BuBr, which is ca. 6 kJ/mol higher than those for the analogous BuI-functionalized system (**cat4**), and a similar tendency was evident for hafnium analogues (**cat8** and **cat7**, respectively). The presence of fluorinated alkyl chains did not increase *Q*_st_ significantly as compared to nonfluorinated analogues.^48^

### Cycloaddition of CO_2_ to Epoxides

**Figure 3:**
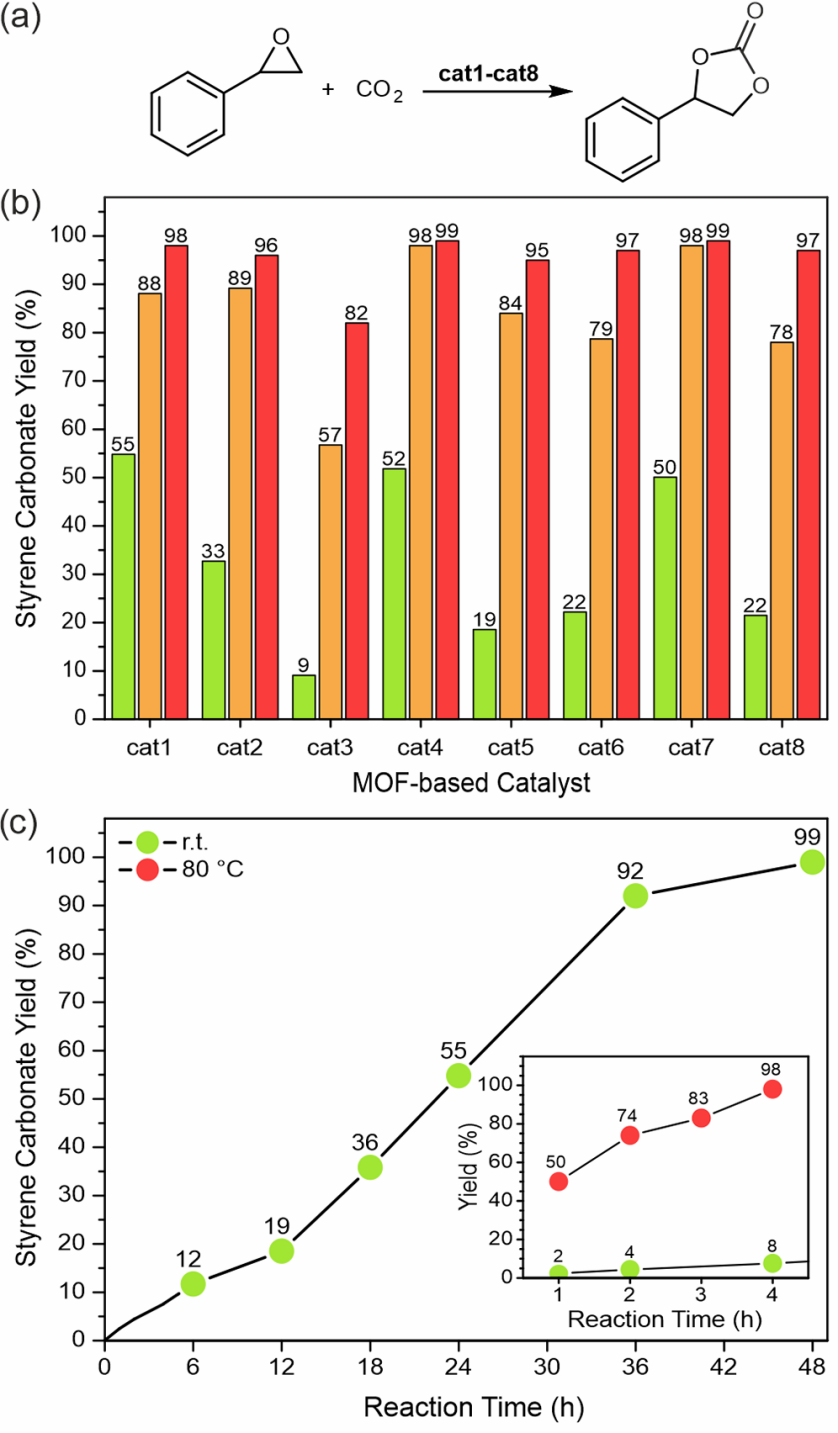
(a) Scheme of the cyclic styrene carbonate synthesis in cycloaddition reaction of CO_2_ to styrene oxide, (b) catalytic activities of **cat1**–**cat8** in the reaction of CO_2_ with styrene oxide at room temperature after 24 h (green), at 60 °C (orange), and at 80 °C (red) after 4 h; (c) time-dependent analysis of **cat1** performance at room temperature (25 °C) during 48 h; the inset shows a comparison of **cat1** at 25 and 80 °C during the first 4 h.

Given the established structural composition, as well as the high porosity and stability of **cat1**–**cat8** materials, we investigated their activities as 2-in-1 catalysts in reactions of various epoxides with CO_2_. Initially, we challenged our catalysts in a model reaction of styrene oxide with CO_2_ under very mild conditions, similar to those described by Beyzavi et al.^15^ Our first catalytic tests were performed at room temperature (25 °C) under autogenous CO_2_ pressure (3.4–4 bar, depending on the reaction temperature) for over 24 h, without the addition of a solvent or an external co-catalyst. The increased reaction pressure helps to avoid contamination from the outside atmosphere and the use of large volumes of gases in these reactions. In our system, we observed a slight decrease of the reaction yield after lowering the CO_2_ pressure to 1–3 bar (see Figure S62c). We observed that **cat1**–**cat8** were highly active in the selective formation of styrene carbonate (Figure 3a,b).^49^ We also discovered that the catalytic performance of **cat1**–**cat8** strongly depended on the functionality introduced during the postsynthetic modification step. The highest styrene carbonate yield was achieved for **cat1** in only 24 h, and its performance was followed by those of **cat4** and **cat7**. All of these systems contain iodide anions as nucleophiles and are more efficient than their bromide analogues.

To gain further insight into the development of the catalytic activity of the prepared systems, we selected the most active **cat1** material and observed its performance for 48 h in the reaction of CO_2_ with styrene oxide at room temperature (Figure 3a). A slow increase of the styrene carbonate formation was observed during the first 24 h of the reaction. A more rapid increase was noticed after 36 h, reaching the yield of 92%. This nonlinear time-dependent performance might result from the kinetic barrier of used substrates caused by their lower diffusivity through the MOF’s bulk at room temperature. To overcome these limitations, we repeated this experiment at a higher temperature of 80 °C (Figure 3a). In this case, the complete formation of styrene carbonate was achieved in only 4 h.

Encouraged by these findings, in the next step, we tested the performance of **cat1**–**cat8** at higher temperatures, i.e., 60 and 80 °C. At 80 °C, all catalysts, except **cat3**, had a nearly quantitative formation of styrene carbonate after only 4 h (Figure 3b). More diverse results were obtained when the catalytic tests were performed at 60 °C (Figure 3b). In this case, the highest yields of styrene carbonate were recorded for **cat4** and **cat7**, which were functionalized with butyl iodide. This result is consistent with the activity trend observed at 25 °C. Notably, we did not observe a significant influence of the alkyl chain length on the activity of the catalyst (**cat1** vs **cat4**; Figure 3b); however, in other studies, the effect of charge separation has been discussed.^31,50^ Moreover, we did not observe any significant influence on the catalytic activity of the metal centers (Zr vs Hf) for systems supported by bromide (**cat5** vs **cat8**) and iodide (**cat4** vs **cat7**) counterions, bearing in mind that catalytic systems featured with bromide anions (**cat5** and **cat8**) were less active than their iodide analogues. The difference in activity between Br^–^ and I^–^, known as the anion effect in commonly used external co-catalysts (for example TBAB vs TBAI),^50^ has been previously discussed for homogenous systems, and here we show that this trend is present in heterogeneous systems as well. Moreover, by comparing the catalytic performance of **cat1–cat3**, we observed the decrease of their activity following the ortho < meta < para trend of isomers of the alkylpyridinium cation. This effect might be associated with the change of the steric and electronic properties of the introduced moieties; conversely, the lower activity of **cat3** may also result from the decreased iodide loading in this material, due to the lower alkylation efficiency for **SALI-2-Py(Zr)**. In their recent work, Ji et al.^37^ have noted that the postsynthetic alkylation of pyridine in the ortho-position is also a difficult process. The **cat6** material, bearing fluorinated alkyl chains, exhibited only a moderate activity of 22% yield of styrene carbonate at room temperature.

Having found the optimal conditions for carrying out the catalytic reaction between styrene oxide and CO_2_, we next analyzed the scope and versatility of the best candidate catalyst **cat1** in the cycloaddition of CO_2_ to other epoxides under optimized reaction conditions. The corresponding results are collected in Table 2. Excellent conversion yields were achieved for aliphatic epoxides (Table 2, entries 1–4), which are regarded as more active compared to styrene oxide and often used in catalytic tests. In the case of the reaction with a bifunctional 1,4-diepoxybutane, we observed the formation of two products bearing one and two carbonate groups (Table 2, entry 5). Remarkably high yields were also obtained for less reactive aromatic epoxides, with more bulky substituents (Table 2, entry 7) or bearing electron-withdrawing groups, including 4-chloro- and 4-fluorostyrene oxide, (Table 2, entries 8 and 9). Finally, for cyclohexene oxide, low conversion was observed (∼4%), since this substrate usually requires more forcing reaction conditions.^51,52^

**Table 2:**
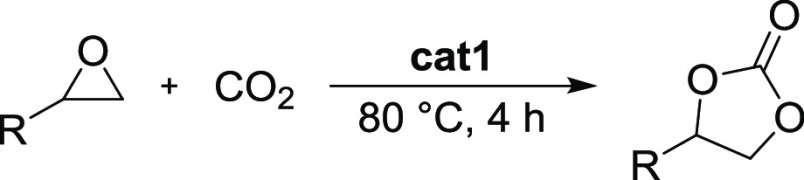

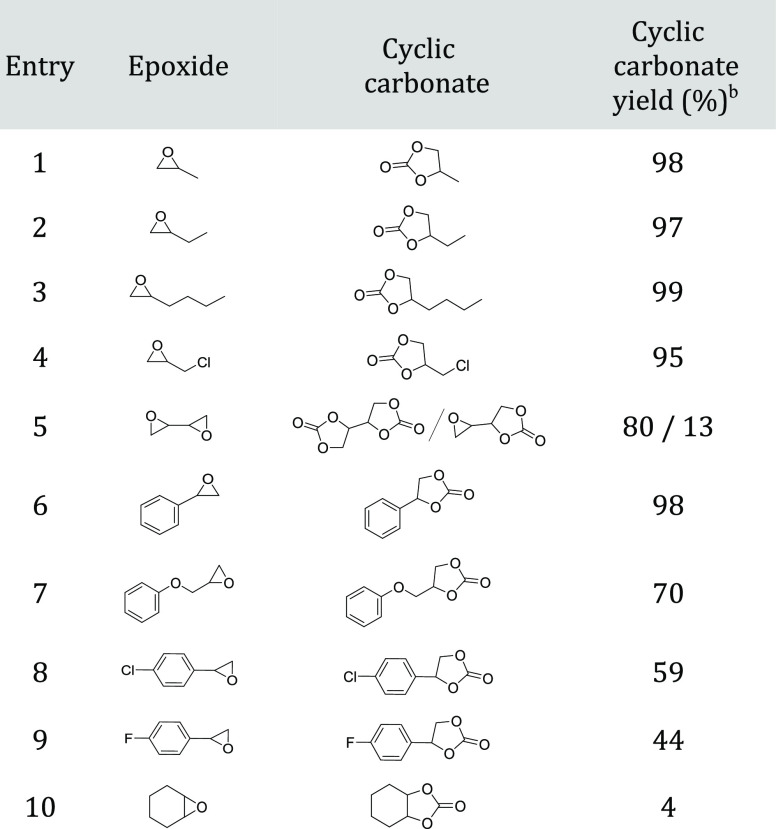
Catalytic Activity of **cat1** in Cycloaddition of CO_2_ to Different Epoxides^a^^,^^b^

a Reaction conditions: 1 mol % **cat1**, 10 μL of epoxide, 4 h, 80 °C, and no solvent.

b Based on ^1^H NMR analysis of the reaction mixture using mesitylene as the internal standard.

The reusability tests of **cat1** in the reaction of CO_2_ with styrene oxide were then performed. As a result, a significant decrease of the cyclic carbonate yield was observed just after the second run (Figure S66a). The performed PXRD, TEM, and SEM studies confirmed that the morphology and crystallinity of **cat1** after catalysis were maintained (Figures S67, S69, and S75). However, the ^1^H NMR analysis of the recycled catalyst (Figure S68) revealed the reduction of the amount of the MeI-4-PyCO_2_^–^ ligand as compared to that of **cat1**. Moreover, XPS and EDS analyses were performed to study the content of iodide anions after each catalytic cycle. These results demonstrate a fast depletion of I^–^ from the MOF framework (Figures S76–S78 and Tables S4 and S5). Therefore, the remodification experiment of the recycled **cat1** was attempted, which resulted in a full recovery of the initial activity of **cat1**.

### Mechanistic Considerations

**Figure 4:**
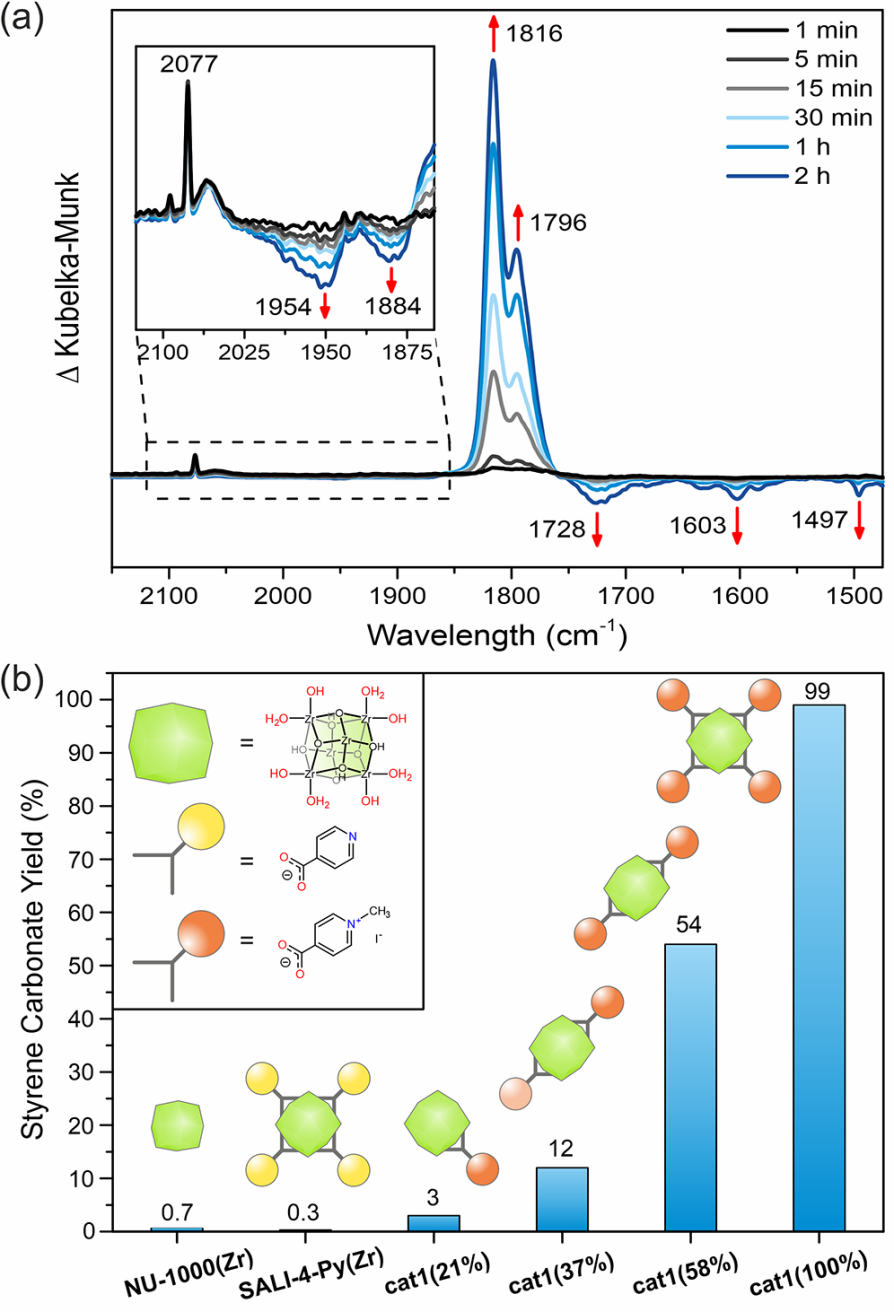
(a) Time-resolved difference DRIFT spectra of **cat1** with styrene oxide at 80 °C under 1 atm of CO_2_ and (b) influence of the amount of pyridinium moieties introduced into **SALI-4-Py(Zr)** by alkylation with methyl iodide on the catalytic activity of **cat1** in the formation of styrene carbonate.

To construct an effective all-in-one catalyst, several factors should be considered. One of the important questions is whether the catalytic process takes place on the surface or inside the crystal, which relates to the diffusion rates of the reactants. Slow diffusion might be an issue when heterogeneous, especially microporous, MOFs are used as catalysts. Typically, larger pores should allow for faster diffusion of reactants inside the porous network. To answer this question and get insight into the initial step of the catalytic process, we resorted to in situ DRIFT measurements and monitored the progress of the reaction between CO_2_ with styrene oxide at its early stage in the presence of **cat1**. The DRIFT spectra were measured under static conditions (80 °C, 1 atm of CO_2_) and were referenced to **cat1** with styrene oxide (at 80 °C) before the introduction of CO_2_. The resulting difference DRIFT spectra after the introduction of 1 atm of CO_2_ are presented in Figure 4a.^53^ The appearance of new strong bands at 1816 and 1796 cm^–1^ from emerging styrene carbonate was observed starting from the first minute of the reaction, and it was accompanied by a decrease in intensity of the bands at 1954, 1884, 1728, 1603, and 1497 cm^–1^, corresponding to unreacted styrene oxide.^54^ Based on these results, we conclude that the catalytic process at 80 °C is not limited by diffusion and occurs not only on the crystal surface but also mainly inside the MOF pores.

We subsequently tested the effect of the number of alkylated pyridinium moieties inside the MOF framework on the activity of the 2-in-1 catalysts. To this end, we designed control experiments using **NU-1000** before and after the SALI reaction and with various ratios of pyridine-4-carboxylate and pyridinium-4-carboxylate attached to the MOF nodes. The tests were conducted under optimized reaction conditions (4 h, 80 °C, 1 mol % catalyst, solvent- and co-catalyst-free). In agreement with our expectations, when **NU-1000(Zr)** or **SALI-4-Py(Zr)** was used, only a small amount of styrene carbonate was formed (Figure 4b, entries 1 and 2). Furthermore, we prepared a series of materials with an increasing number of alkylated pyridines per Zr_6_-node denoted as **cat1** (0–100%), as shown in Figure 4b (entries 3–6). These results unambiguously show that the second functionalization step is required to obtain the active 2-in-1 catalytic system. Noteworthily, the comparison of the number of alkylpyridinium (MeI-4-PyCO_2_^–^) moieties per zirconium node in **cat1** vs its catalytic activity shows a nonlinear trend; hence, we conclude that the alkylpyridinium moieties act in a cooperative mode during the catalytic process. This observation agrees with the kinetic studies on homogenous systems, where it was demonstrated that the relation between the co-catalyst concentration and the reaction rate is of the second order.^55^

**Figure 5:**
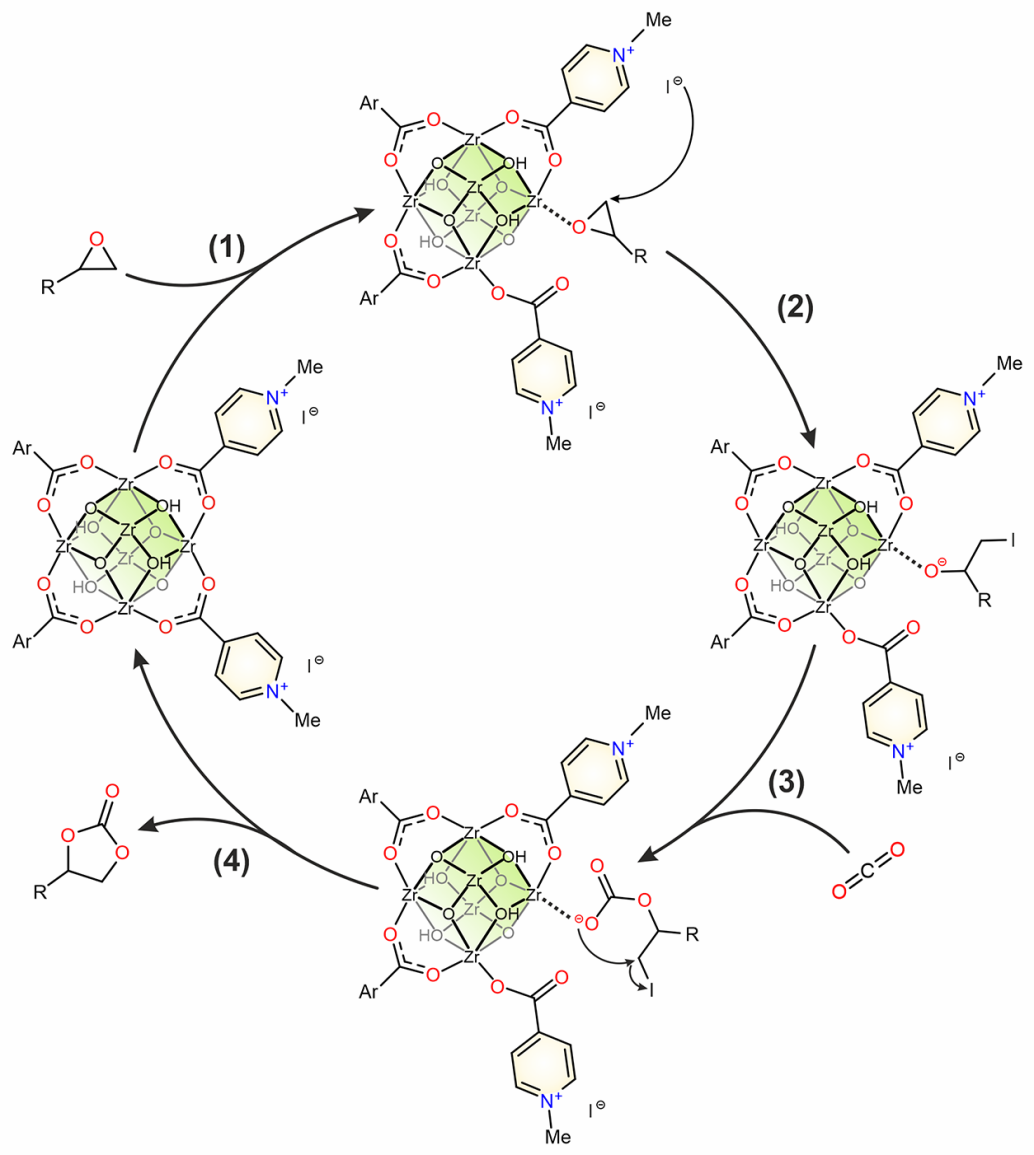
Proposed catalytic cycle for the formation of cyclic carbonate catalyzed by **cat1**; Ar = MeI-4-Py.

Based on the structural features and the catalytic activity of the 2-in-1 catalysts and previous reports,^32,36,55^ we propose a plausible catalytic cycle (Figure 5). In the first step, the epoxy ring is activated by coordinating to the Zr^4+^ center (Lewis acid site). Direct epoxide coordination to the metal center should be possible, due to the coordination mode lability of the nonstructural carboxylate ligands attached to the metal nodes.^47^ Subsequently, the halogen anion (X^–^ = Br^–^, I^–^) counterbalancing alkylpyridinium cations coordinated to the Zr_6_-node participates in the nucleophilic attack on the less sterically hindered carbon atom of the activated substrate to open the epoxide ring yielding the corresponding intermediate (step 2, Figure 5). This haloalkoxide intermediate then interacts with carbon dioxide and the insertion of CO_2_ occurs (step 3, Figure 5). In the next step, intramolecular cyclization takes place yielding the corresponding cyclic carbonate with concomitant regeneration of the catalyst (step 4, Figure 5). The recycling test demonstrated that alkylpyridinium moieties counterbalance the halide ions, which are necessary for completion of the catalytic cycle.

## Conclusions

In summary, we have devised a new family of 2-in-1 MOF-based catalytic systems for cycloaddition of carbon dioxide to epoxides under mild conditions. We have shown that tandem postsynthesis modification of the **NU-1000(M)** (M = Zr, Hf) framework converts a nonactive system into a highly active catalyst. The postsynthetic functionalization of the metal nodes allowed for the precise allocation of both the Lewis acid sites and the nucleophilic halide ions throughout the mesoporous framework. The proximity of the components of the catalytic system improves the cooperation of the Lewis sites and co-catalyst moieties, which correlates with the previously reported kinetic studies on homogenous systems. We believe that our findings will in the future help design new multimodal catalytic systems for other types of heterogeneous reactions.

## Supporting Information Available

Materials, synthesis, and characterization methods of prepared materials before and after the performed functionalization; solution ^1^H and ^13^C NMR spectra; solid-state ^13^C CP-MAS NMR spectra; TGA data; PXRD studies; SEM/EDS images; DRIFT studies; and N_2_ and CO_2_ sorption data (PDF)
