# Supplementary material for: Quest for an Efficient 2-in-1 MOF-Based Catalytic System for Cycloaddition of CO2 to Epoxides under Mild Conditions
Source: ACS Appl Mater Interfaces. 2021 Feb 9;13(7):8344–52. doi: 10.1021/acsami.0c20437 (PMC8023534; doi:10.1021/acsami.0c20437)
Supplement: Supplementary file 1 — am0c20437_si_001.pdf [file am0c20437_si_001.pdf]

Supporting Information for

Quest for an Efficient *2-in-1* MOF-Based Catalytic System for Cycloaddition of CO_2 to Epoxides under Mild Conditions

Marzena Pander, Mateusz Janeta, Wojciech Bury*

Faculty of Chemistry, University of Wrocław, 14 F. Joliot-Curie, 50-383 Wrocław, Poland

*corresponding author: wojciech.bury@chem.uni.wroc.pl

Table of Contents

S1. Materials 3

S2. Instrumentation 3

S3. Experimental procedures 4

S3.1. Synthesis of NU-1000(M) materials 4

S3.2. SALI reaction with pyridinecarboxylic acids[4,5] 4

S3.3. Alkylation of SALI-*n*-Py(M) (*n* = 2-4, M = Zr, Hf) with selected haloalkanes 5

S3.4. Catalytic reactions of carbon dioxide with epoxides 5

S4. Structural characterization of prepared MOF-based materials 6

S4.1. PXRD analysis 6

S4.2. NMR spectra of liquid samples 7

S4.3. CP-MAS NMR spectra of selected materials 11

S4.4. Nitrogen sorption studies and BET surface area calculations 13

S4.6. Carbon dioxide sorption studies and isosteric heats of CO_2 adsorption (Q_{st}) 17

S4.6. TGA-DTG analysis 20

S4.7. SEM images and EDS analysis 23

S4.8. DRIFT spectra 32

S4.9. VT-DRIFTS 34

S5. Studies on catalytic performance of prepared *2-in-1* catalysts 35

S5.1. Probing cat1 as a model catalyst for cycloaddition of CO_2 to epoxides 35

S5.2. *In situ* IR studies on interactions of CO_2 with cat1 and NU-1000(Zr) 38

S5.3. The recyclability tests of cat1 in reaction of CO_2 with styrene oxide 40

S5.4. Re-insertion of CH_3I-4-$PyCO_2^-$ in cat1 (after catalysis) 47

S6. References 48

S1. Materials

Zirconium oxide dichloride octahydrate ($ZrOCl_2 \times 8H_2O$, Alfa Aesar, 98%), Hafnium oxide dichloride octahydrate, ($HfOCl_2 \times 8H_2O$, Alfa Aesar, 98%), benzoic acid (Acros, 99%), hydrochloric acid (Stanlab Sp. J., 35-38%), trifluoroacetic acid (TCI, 99%), pyridine-4-carboxylic acid (**4-PyCOOH**, Roth, 98%), pyridine-3-carboxylic acid (**3-PyCOOH**, J.T.Baker, 99%), pyridine-2-carboxylic acid (**2-PyCOOH**, Sigma-Aldrich, 99%), iodomethane (Sigma-Aldrich, 99%), 1-iodobutane (Sigma-Aldrich, 99%), 1-bromobutane (POCH, 99%), 1*H*,1*H*,2*H*,2*H*-perfluorohexyl iodide (Sigma-Aldrich, ≥95%), 1,2-epoxypropane (Sigma-Aldrich, ≥99.5%), 1,2-epoxybutane (Sigma-Aldrich, 99%), 1,2-epoxyhexane (Acros, 97%), epichlorohydrin (Acros, 99%), 1,3-butadiene diepoxide (Sigma-Aldrich, 97%), styrene oxide (Sigma-Aldrich, 97%), 1,2-epoxy-3-phenoxypropane (Acrcos, 99%), 4-chlorostyrene oxide (Alfa Aesar, 98%), 4-fluorostyrene oxide (Alfa Aesar, 98%), 1,2-epoxycyclohexane (EGA-Chemie, 95-98%), 1,2-epoxycyclododecane (Fluka, 95%), *trans*-stilbene oxide (Acros, 99%), mesitylene (Alfa Aesar, 98%) were used without further purification. 1,3,6,8-tetrakis(p-benzoic-acid)pyrene (H_4TBAPy) was synthesized as described in literature.[1]

Solvents: ethanol (J.T.Baker, 99.5%), acetonitrile (POCH, 99%), *N,N*-dimethylformamide (Chempur, 98%), toluene (Stanlab Sp. J., 98%), acetone (Chempur, 98%), deuterated chloroform ($CDCl_3$, stab. with Ag, Sigma-Aldrich, ≥99.8 atom% D), deuterated dimethylsulfoxide (DMSO-d_6, Deutero, 99.8 atom% D), deuterated acetone (acetone-d_6, Sigma-Aldrich, 99.9 atom% D), deuterated sulfuric acid (D_2SO_4, Sigma-Aldrich, 96-98% solution in D2O, 99.5 atom% D), were used as received.

S2. Instrumentation

^1H nuclear magnetic resonance (NMR) spectra in solutions were recorded using a Bruker Avance 500 spectrometer at 298 K and were calibrated on the residual solvent signal (DSMO-d_6: 2.50 ppm, $CDCl_3$: 7.26 ppm). The NMR samples of obtained MOFs before and after functionalization were prepared by digesting approximately 1 mg of dried material in D_2SO_4 and then diluting it with 0.6 ml of DMSO-d_6. The ^{13}C cross-polarization magic angle spinning (CP-MAS) nuclear magnetic resonance measurements were carried out on a Bruker Avance III HD 600 MHz spectrometer using a CP-MAS probe and 3.2 mm rotor with recycle delay of 5 s. All spectra were recorded at room temperature and referenced using adamantane peak at 38.5 ppm. The operating spinning rate was set at 19 kHz and the number of scans was 1024. Diffuse reflectance infrared Fourier transform (DRIFT) spectra were collected on Nicolet iS50 FT-IR Spectrometer (Thermo Scientific) with a Praying Mantis DRIFT accessory. The spectra were collected in a 4000-400 cm^{-1} range with number of scans set to 128. Samples were prepared under air atmosphere by grinding in a mortar with KBr and then placed under N_2 purge for collection time. Variable-temperature DRIFTS were collected using high temperature reaction chamber (Harrick Scientific Products Inc), accessory with temperature control performed with EZ-ZONE software with a heating step set to 1 °C/min under the N_2 purge. Powder XRD data were collected on Bruker D8 ADVANCE diffractometer equipped with a copper lamp (CuKα radiation, λ = 1.5406 Å) at 30 kV and 40 mA with a slit of 0.1°. Standard measurements were done in 2θ range of 4°-40° with a 2θ step of 0.008° and a counting time of 0.5 s. All gas sorption isotherms were measured on a Micromeritics ASAP 2020. Prior to the measurements, the samples were degassed at 120 °C for 24 h (for **NU-1000(M)**, **SALI-*n*-Py**, M = Zr, Hf, n = 2, 3, 4) or 80 °C for 16 h (for **cat1–cat8**). N_2 sorption measurements were performed at 77 K using a liquid N_2 bath, whereas for CO_2 sorption measurements chilled water/ethylene glycol bath was used for temperature control (measurements were performed

at 273 K, 283 K and 293 K). SEM images were collected on a Hitachi S-3400N-II variable-pressure scanning electron microscope. Samples were sputter-coated with 7 nm Au to facilitate viewing by SEM. Energy dispersive X-ray spectra (EDS) were obtained using an EDS Thermo Scientific Ultra Dry system. Thermogravimetric and differential thermal analyses (simultaneous TG-DTA) were recorded on a Setaram SETSYS 16/18 instrument. Samples for thermogravimetric characterization were placed in alumina crucibles in synthetic air (O_2:N_2 = 20:80) (flow rate: 1 dm^3/h) at heating rate 5 ºC/min, samples were studied between 30 and 1000 ºC. XPS spectra were recorded on a SPECS UHV/XPS/AES system equipped with a hemispherical PHOIBOS 100 analyzer operating in the fixed analyzer transmission (FAT) mode and a dual Mg/Al X-ray source, Mg-Kα X-ray source excitation (1253.6 eV) was operated at 250 W and 12 kV. The base pressure in the analysis chamber was less than 5×10^{-10} mbar. The spectrometer energy scale was calibrated with Au 4*f 7/2*, Ag 3*d 5/2*, and Cu *2p 3/2* lines at 84.2, 367.9, and 932.4 eV, respectively. The binding energies were calibrated using carbon C 1s (C-C, C-H in aromatic) with BE at 284.8 eV as a reference. Spectra were processed and fitted by SPECLAB2 and CasaXPS v.2.3.19 software using Gaussian-Lorentzian curve profile and Shirley baseline.

S3. Experimental procedures

S3.1. Synthesis of NU-1000(M) materials

NU-1000(Zr): The material was synthesized according to the procedure described by Islamoglu et al. where in addition to the benzoic acid, a trifluoroacetic acid is used as co-modulator.[2]

NU-1000(Hf): The synthetic procedure[3] was modified in respect to the same strategy as described above (by using TFA as a co-modulator). 0.125 g of hafnium oxide dichloride octahydrate ($HfOCl_2 \times 8H_2O$, 0.305 mmol) and 2.00 g of benzoic acid (16.4 mmol) were dissolved in 8 mL of DMF in a glass vial. The solution was then incubated at 100 °C for 1 h and cooled down to room temperature. 40 mg of H_4TBAPy (0.059 mmol) and 40 µL of TFA (0.53 µmol) were added to the clear solution, sonicated for 15 min and heated at 100 °C for 24 h. Obtained yellow powder was then washed three times with DMF and activated with 8 M HCl to remove coordinated benzoate groups on Hafnium node according to the standard procedure.

Synthesized **NU-1000(M)** (M = Zr, Hf) materials were characterized by powder X-ray diffraction (PXRD), proton nuclear magnetic resonance (^{1}H NMR), diffuse reflectance infrared Fourier transform spectroscopy (DRIFTS), thermogravimetric analysis (TGA) and N_2 sorption measurements.

S3.2. SALI reaction with pyridinecarboxylic acids[4,5]

In each reaction, 20 mg of activated **NU-1000(Zr)** (0.009 mmol) or 25 mg of activated **NU-1000(Hf)** (0.009 mmol) were soaked in a 5mL of 0.03 M solution of pyridine-4-carboxylic acid (**4-PyCOOH**) in EtOH at 60 °C for 24 h. After that time, the supernatant was removed and the solid was soaked in a 15 mL of a fresh portion of ethanol and incubated at 60 °C for the next 24 h to remove unbound ligand. The obtained material was washed three times with ethanol and dried at 60 °C. The same procedure was applied in case of **NU-1000(Zr)** for other isomers of introduced ligands, namely pyridine-2-carboxylic acid (**2-PyCOOH**) and pyridine-3-carboxylic acid (**3-PyCOOH**). The number of incorporated molecules of pyridinecarboxylic acids was determined by ^{1}H NMR analysis of the samples digested in D_2SO_4/DMSO-d_6 mixture. The obtained materials were characterized by PXRD, DRFITS, TGA and N_2 sorption measurements.

S3.3. Alkylation of SALI-*n*-Py(M) (*n* = 2-4, M = Zr, Hf) with selected haloalkanes

Initially, the alkylation reaction of pyridine moiety in **NU-1000(Zr)** material was performed in toluene at 100 °C based on literature procedure.[4] The synthetic approach was then optimized in acetonitrile at lower temperatures. In that case, 30 mg of **SALI-*n*-Py(Zr)** (*n* = 2-4, 0.012 mmol,) or 30 mg of **SALI-4-Py(Hf)** (0.011 mmol) were soaked in 2 mL of acetonitrile in a microwave reaction vial (Biotage). Then, selected alkyl halide (RX) was added to the solution: 50 µL of methyl iodide (MeI, 114.00 mg, 0.80 mmol) for **cat1** and **cat2**, 100 µL of methyl iodide (MeI, 228.00 mg, 1.60 mmol) for **cat3**, 200 µL of butyl iodide (BuI, 324.00 mg, 1.76 mmol) for **cat4** and **cat7**, 1 mL of butyl bromide (BuBr, 1.27 mg, 9.27 mmol) for **cat5** and **cat8**, and 250 µL 1*H*,1*H*,2*H*,2*H*-perfluorohexyl iodide ($C_6H_4F_9I$, 485.00 mg, 1.27 mmol) for **cat6**. The vial was sealed and placed in Eppendorf ThermoMixer (500 rpm) at 60 °C for 24 h (for **cat1, cat2**, **cat4**, **cat7**) or at 80 °C for 48 h (for **cat3**, **cat5, cat6**, **cat8**). After that time, the obtained solids were washed three times with acetonitrile and dried at 80 °C. The yield of pyridine moiety alkylation was determined by ^1H NMR analysis of the digested samples.

S3.4. Catalytic reactions of carbon dioxide with epoxides

In a typical reaction, a 5 mL vial, equipped with a small magnetic stirring bar, was charged with 0.01 ml of selected epoxide (0.087 mmol), 1 mol% of prepared MOF-based catalyst and 30 mg of solid CO_2 (0.682 mmol). The vial was sealed, and reaction mixture was stirred at 80 °C for 4 h (the CO_2 pressure in a vial was approx. 4 bar). After that time, the vial was cooled in liquid nitrogen bath and the excess of CO_2 was slowly released. The vial was weighted before and after performed reaction to ensure the tightness of the system. Then, 5 µL of mesitylene (*internal standard*, 4.32 mg, 0.036 mmol) was added to the crude reaction mixture and the catalyst was separated by washing it twice with 0.4 mL of $CDCl_3$ (6000 rpm, 2 min). In case of the reaction with 1,3-butadiene diepoxide, the crude reaction mixture was washed with acetone-d_6. For the recycling experiment, the recovered catalyst was washed with acetone three times, and then the dried material was reused for the next cycle. The reaction yield was calculated based on ^1H NMR analysis from the collected supernatant fraction. The NMR spectroscopy also confirmed high selectivity of the catalyst where for all studied epoxides, the cyclic carbonate was the main product with an absence of polycarbonate or hydrolysis products.

S4. Structural characterization of prepared MOF-based materials

S4.1. PXRD analysis

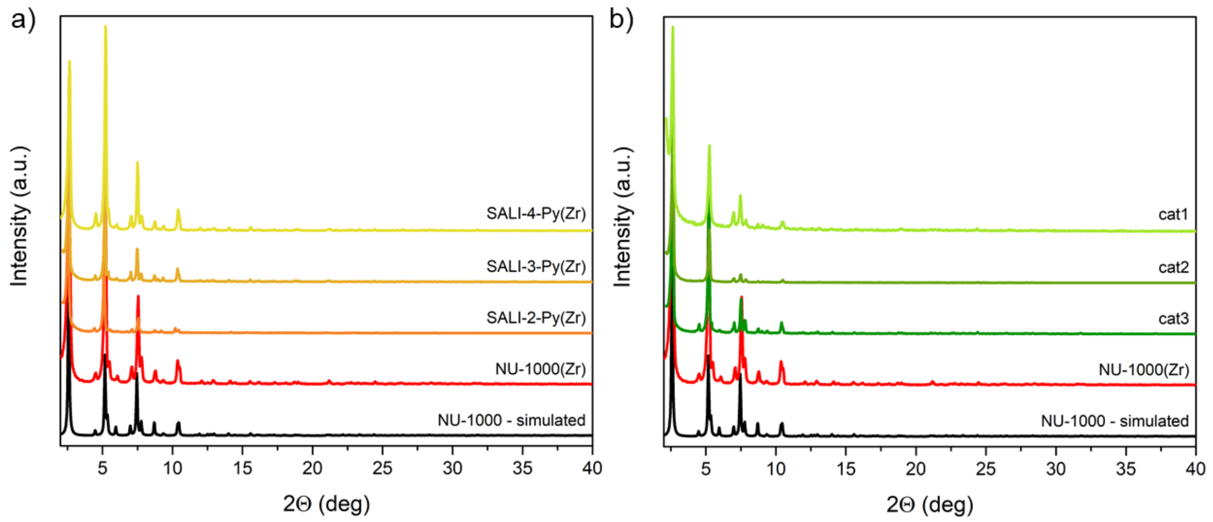

Figure S1. a) PXRD patterns of prepared **NU-1000(Zr)** materials before and after SALI reaction with isomers of 2-, 3- and 4-pyridinecarboxylic acid (***n*-PyCOOH**, where *n* = 2,3,4, respectively). b) PXRD patterns of **cat1–cat3** materials obtained after alkylation of pyridine moieties with methyl iodide.

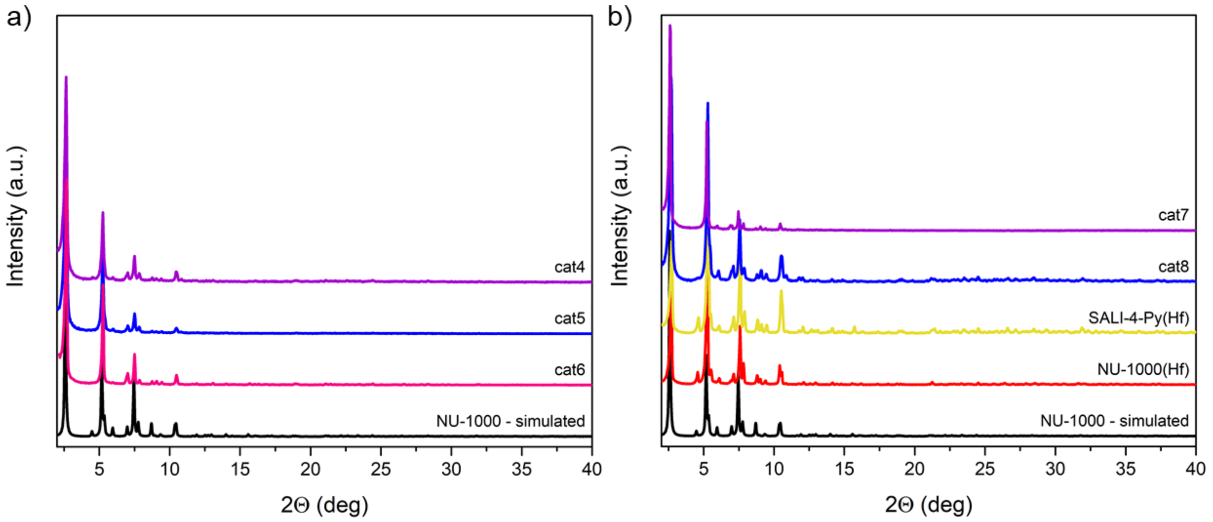

Figure S2. a) PXRD patterns of Zr-based *2-in-1* catalysts **cat4–cat6**. b) PXRD patterns of Hf-based *2-in-1* catalysts **cat7–cat8** compared with **NU-1000(Hf)** before and after SALI reaction.

S4.2. NMR spectra of liquid samples

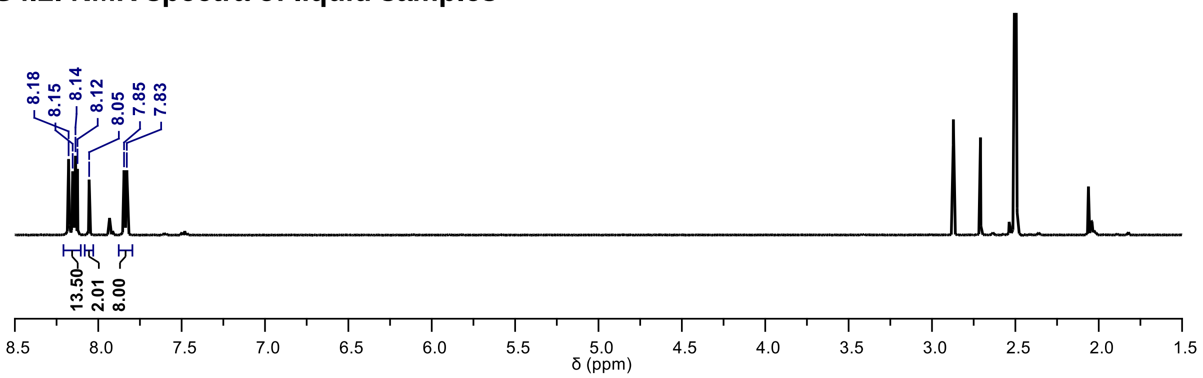

Figure S3. ^{1}H NMR spectrum of **NU-1000(Zr)**.

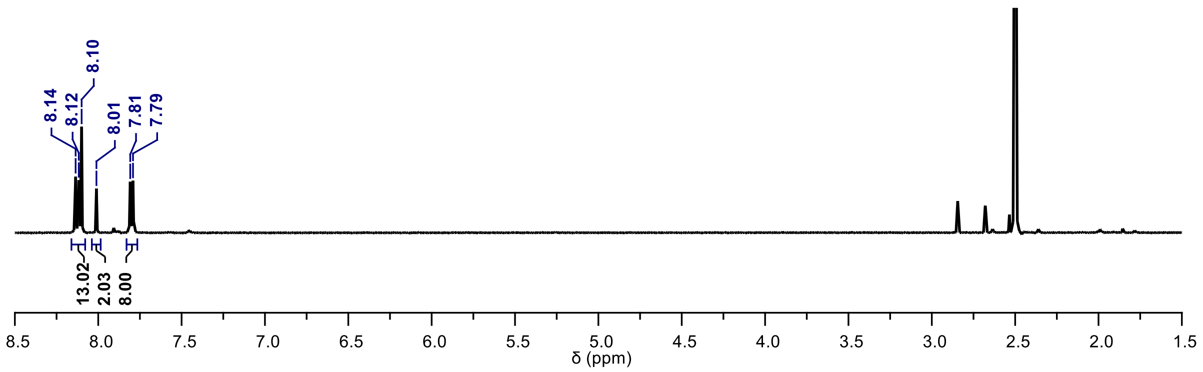

Figure S4. ^{1}H NMR spectrum of **NU-1000(Hf)**.

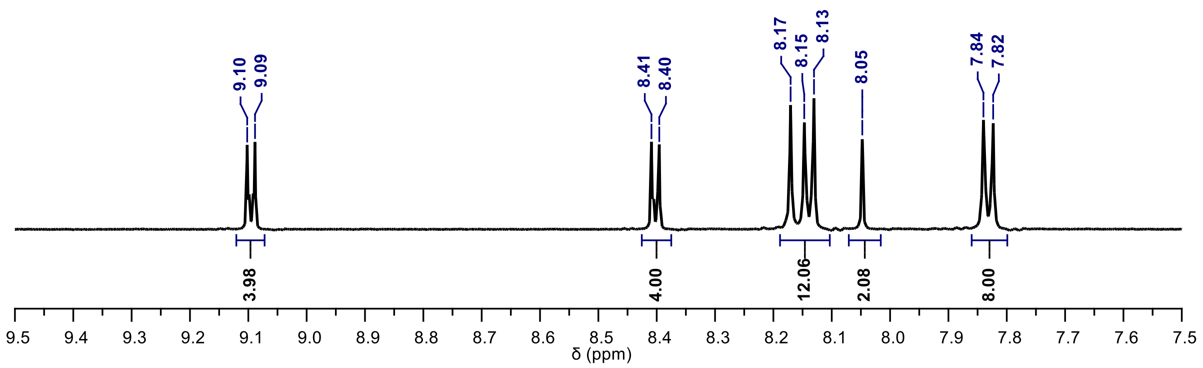

Figure S5. ^{1}H NMR spectrum of **SALI-4-Py(Zr)**.

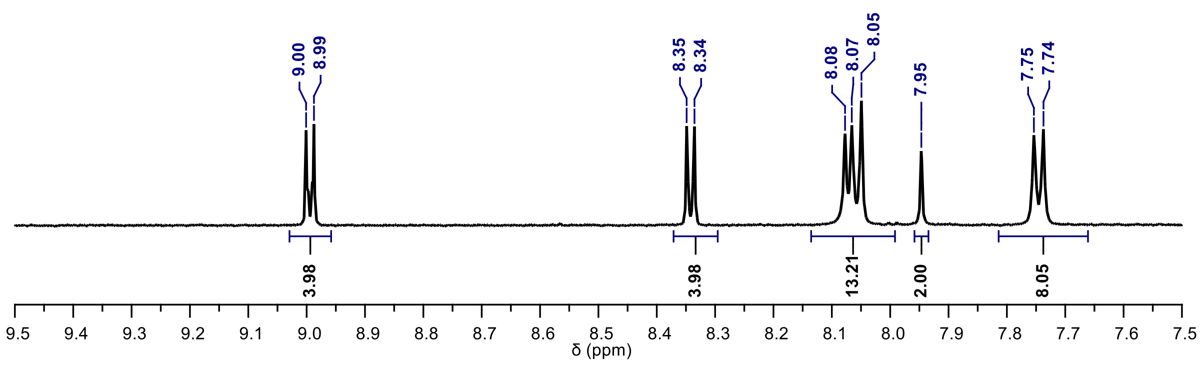

Figure S6. ^{1}H NMR spectrum of **SALI-4-Py(Hf)**.

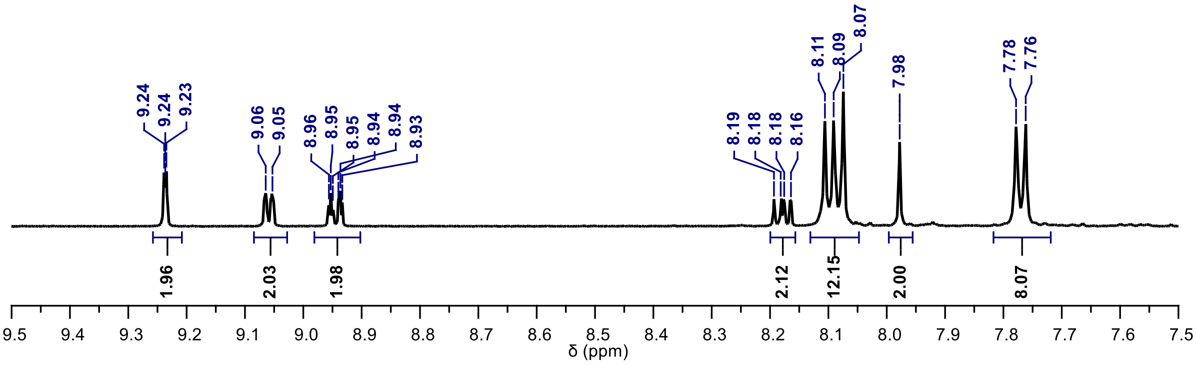

Figure S7. ^{1}H NMR spectrum of **SALI-3-Py(Zr)**.

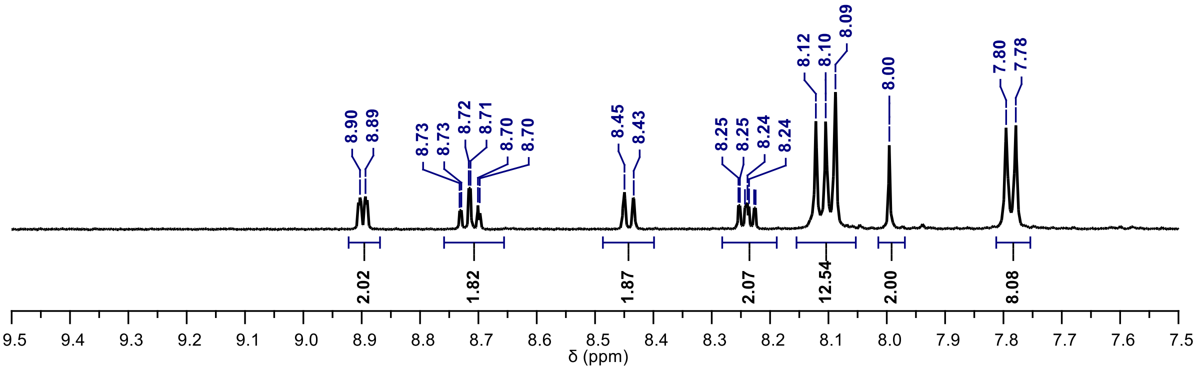

Figure S8. ^{1}H NMR spectrum of **SALI-2-Py(Zr)**.

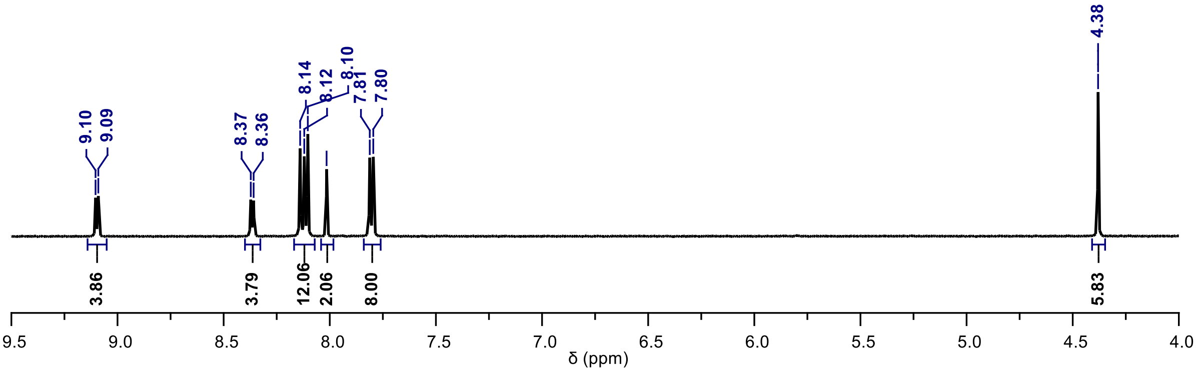

Figure S9. ^{1}H NMR spectrum of **cat1**.

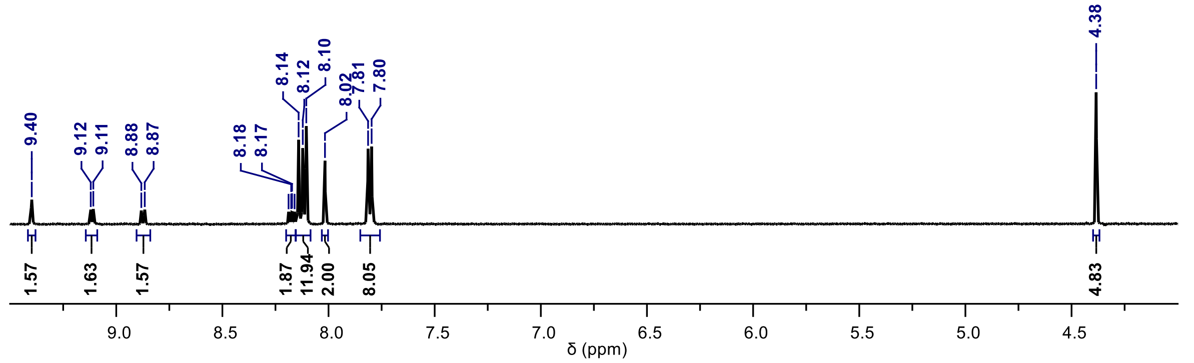

Figure S10. ^{1}H NMR spectrum of **cat2**.

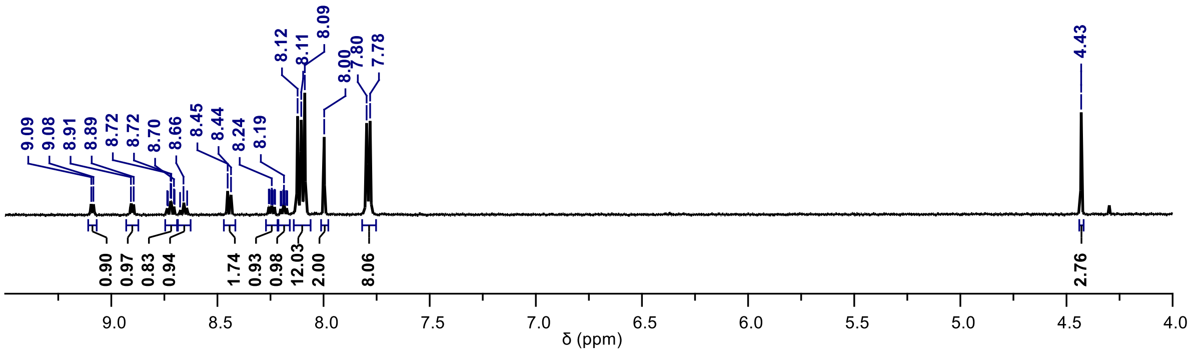

Figure S11. ^{1}H NMR spectrum of **cat3**.
The alkylation yield was 50% (approx. 1.9 CH_3I-2-$PyCO_2^-$ molecules vs 1.8 non-alkylated 2-$PyCO_2^-$ molecules).

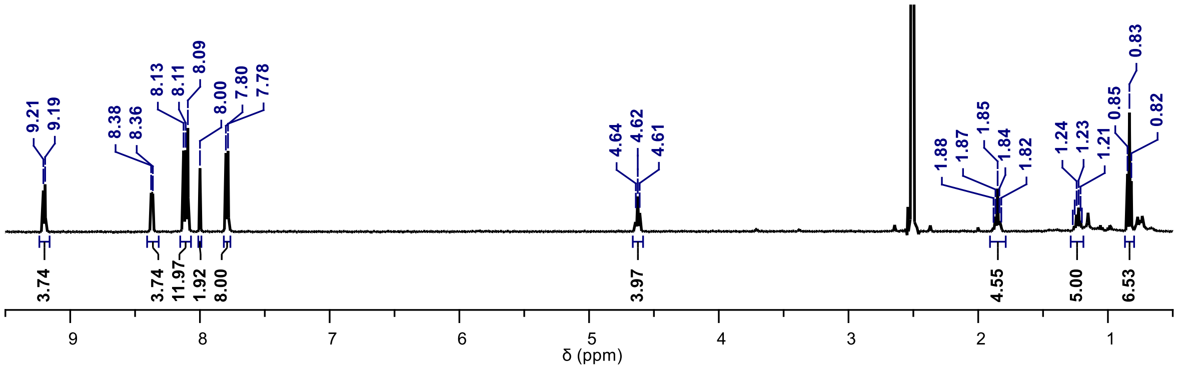

Figure S12. ^{1}H NMR spectrum of **cat4**.

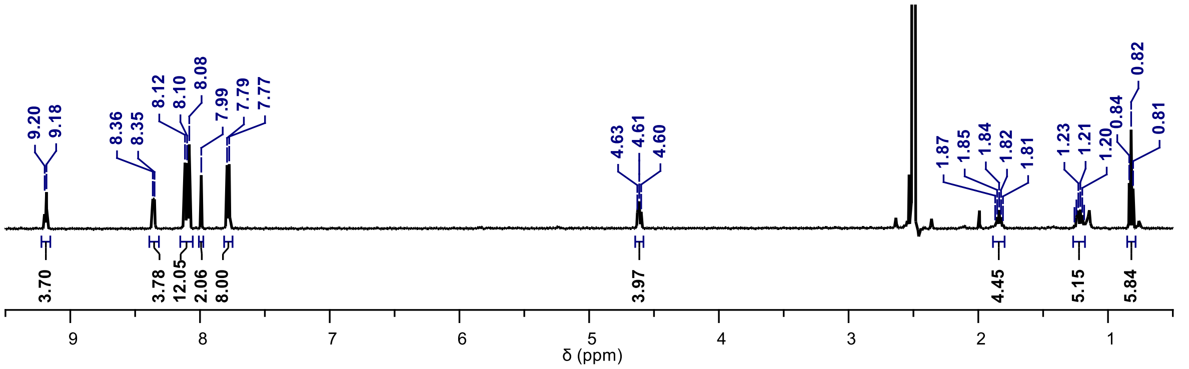

Figure S13. ^{1}H NMR spectrum of **cat5**.

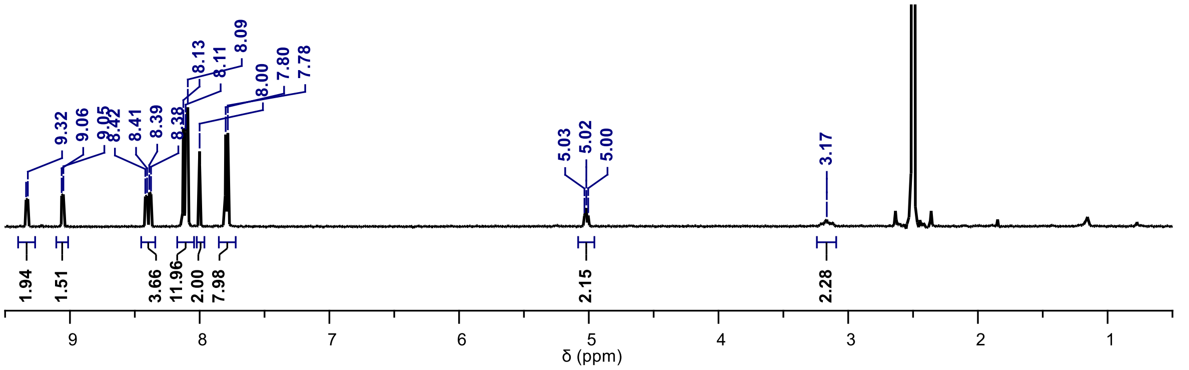

Figure S14. ^{1}H NMR spectrum of **cat6**.
The alkylation yield was 57% (approx. 2.0 $C_6H_4F_9I$-4-$PyCO_2^-$ molecules vs 1.5 non-alkylated 4-$PyCO_2^-$ molecules).

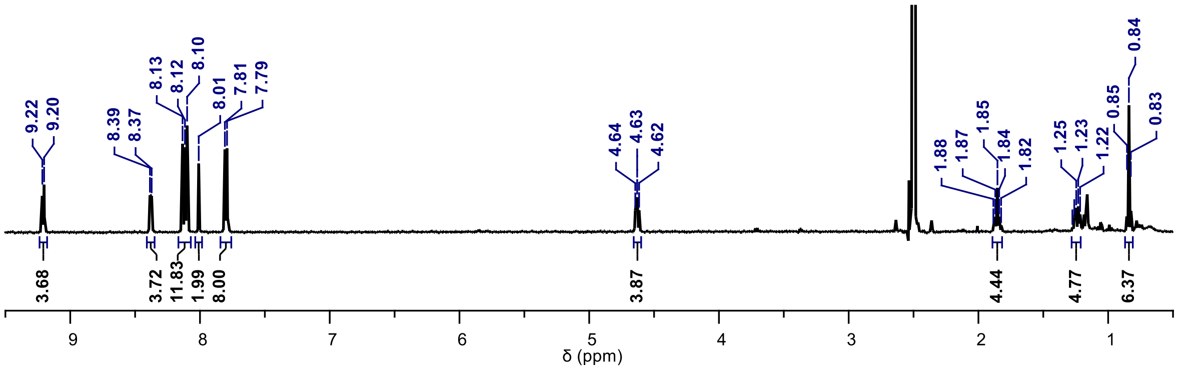

Figure S15. ^{1}H NMR spectrum of **cat7**.

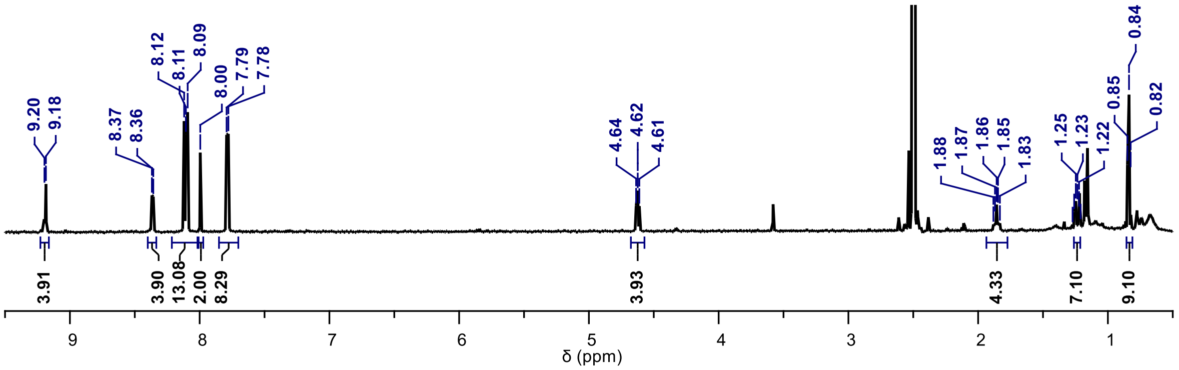

Figure S16. ^{1}H NMR spectrum of **cat8**.

S4.3. CP-MAS NMR spectra of selected materials

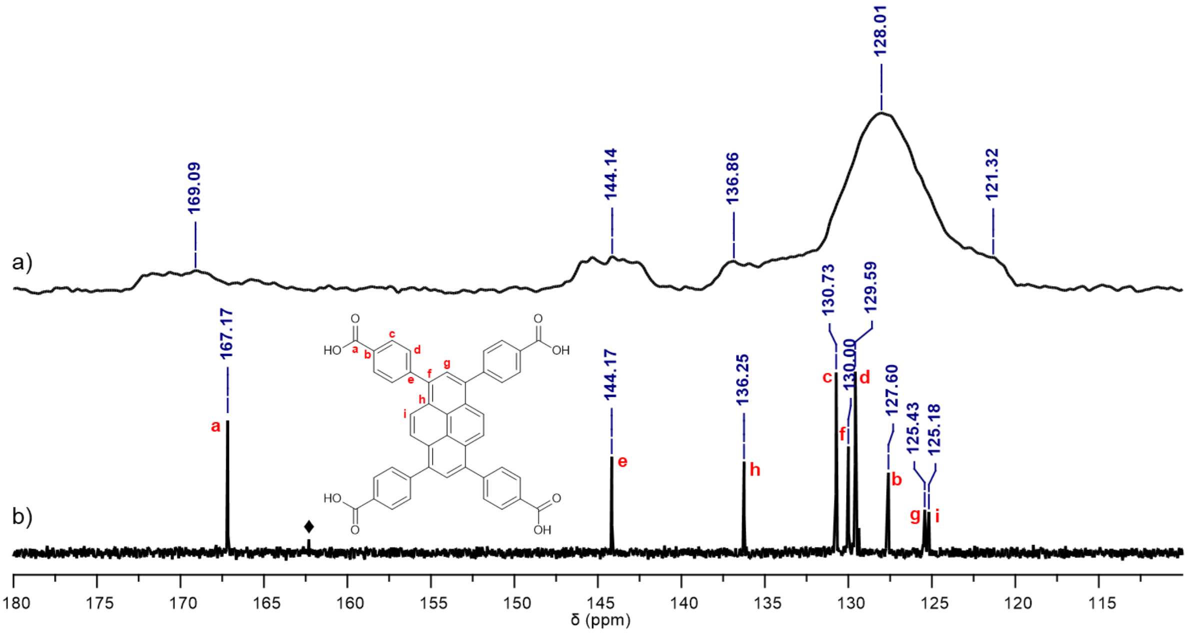

Figure S17. a) ^{13}C CP-MAS spectrum of **NU-1000(Zr)**. b) ^{13}C NMR spectrum of linker H_4TBAPy dissolved in DMSO-d_6; ♦ denotes a residual signal of DMF.

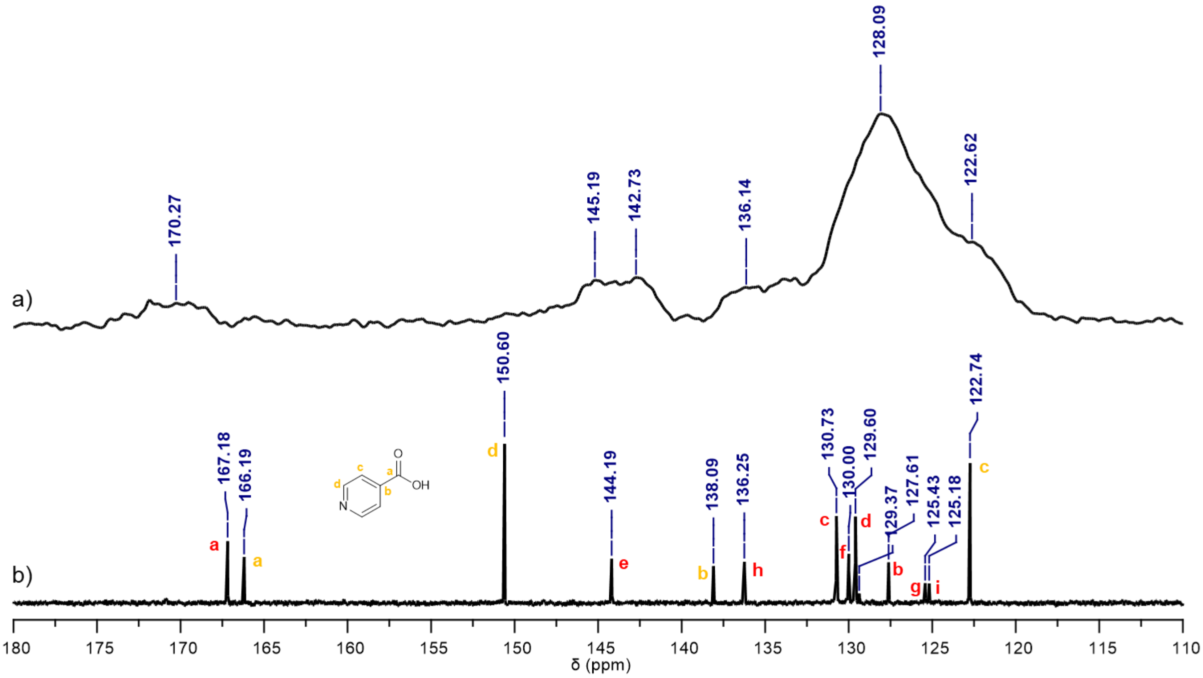

Figure S18. a) ^{13}C CP-MAS spectrum of **SALI-4-Py(Zr)**. b) ^{13}C NMR spectrum of mixture of ligand pyridine-4-carboxylic **4-PyCOOH** and linker H_4TBAPy dissolved in DMSO-d_6.

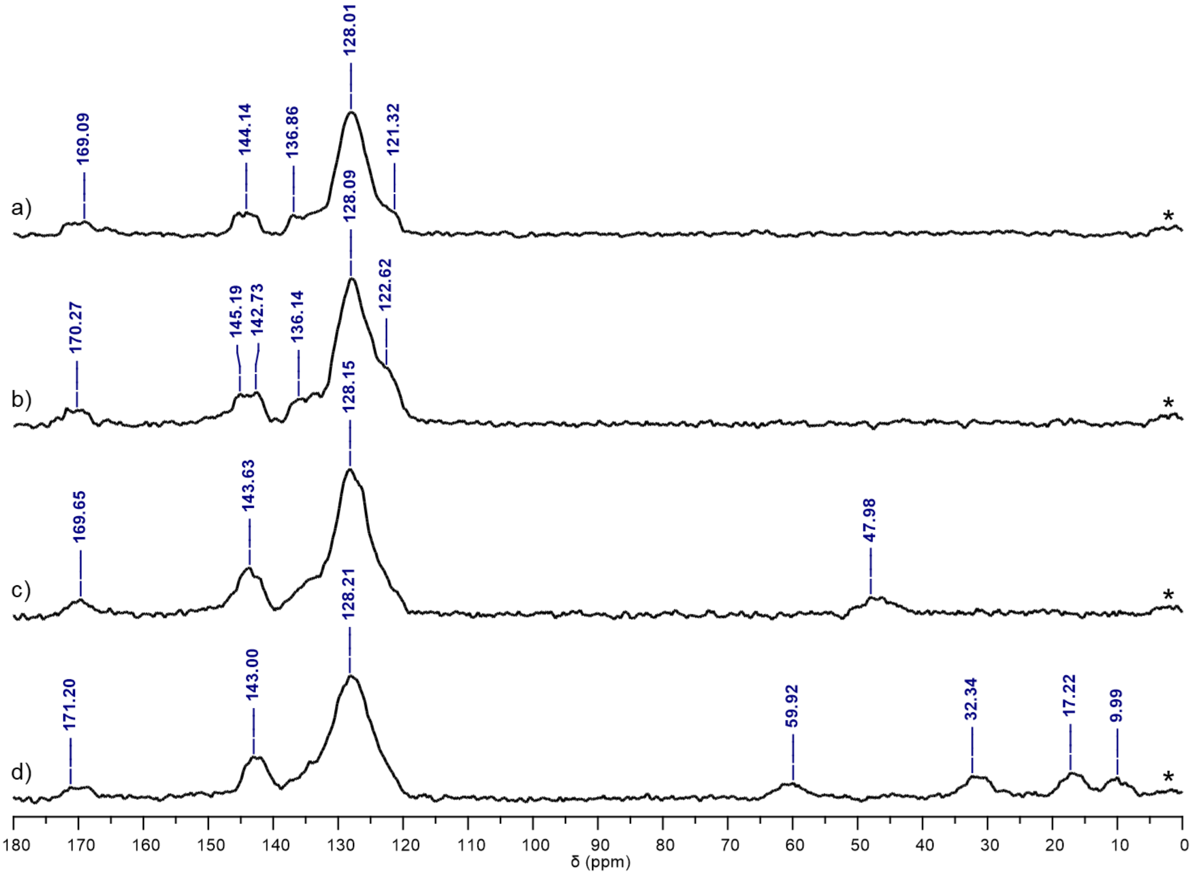

Figure S19. ^{13}C CP-MAS spectra of: a) **NU-1000(Zr)**, b) **SALI-4-Py(Zr)**, c) **cat1**, d) **cat5**; asterisk denotes spinning side bands.

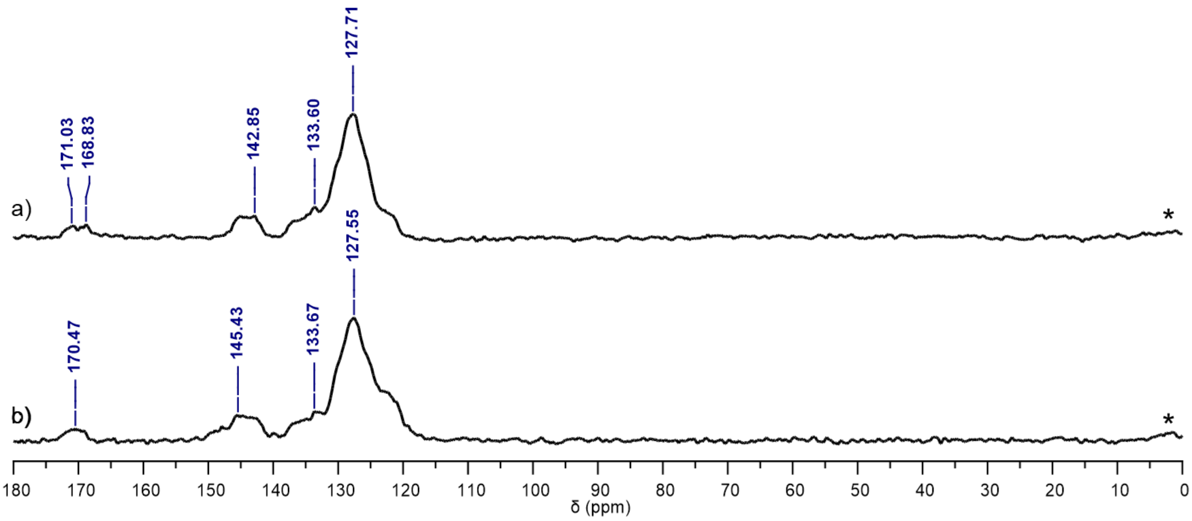

Figure S20. ^{13}C CP-MAS spectra of: a) **NU-1000(Hf)**, b) **SALI-4-Py(Hf)**; asterisk denotate spinning side bands.

S4.4. Nitrogen sorption studies and BET surface area calculations

The Brunauer-Emmett-Teller (BET) theory was used to calculate the specific surface areas of obtained materials. For all isotherm analyses we ensured that the two consistency criteria described by Roquerol et al.[6] and Walton et al.[7] were satisfied. In **Tables S1** are collected obtained BET parameters. The pore sized distribution plots were derived from sorption data by DFT calculations using a carbon slit pore model with a N_2 kernel.

Table S1. BET parameters for Zr-based materials.

#	sample	C	Q_m (cm^3/g STP)	BET Surface Area (m^2/g)
1	**NU-1000(Zr)**	380.6	473.7	2062
2	**NU-1000(Hf)**	404.9	409.7	1783
3	**SALI-4-Py(Zr)**	109.8	441.1	1920
4	**SALI-4-Py(Hf)**	86.9	412.9	1797
5	**SALI-3-Py(Zr)**	110.7	473.5	2061
6	**SALI-2-Py(Zr)**	141.2	452.5	1852
7	**cat1**	140.1	275.9	1201
8	**cat2**	116.0	265.7	1157
9	**cat3**	142.2	349.9	1523
10	**cat4**	131.2	276.9	1205
11	**cat5**	171.7	346.9	1510
12	**cat6**	98.7	307.8	1339
13	**cat7**	133.5	262.4	1142
14	**cat8**	177.1	251.9	1097

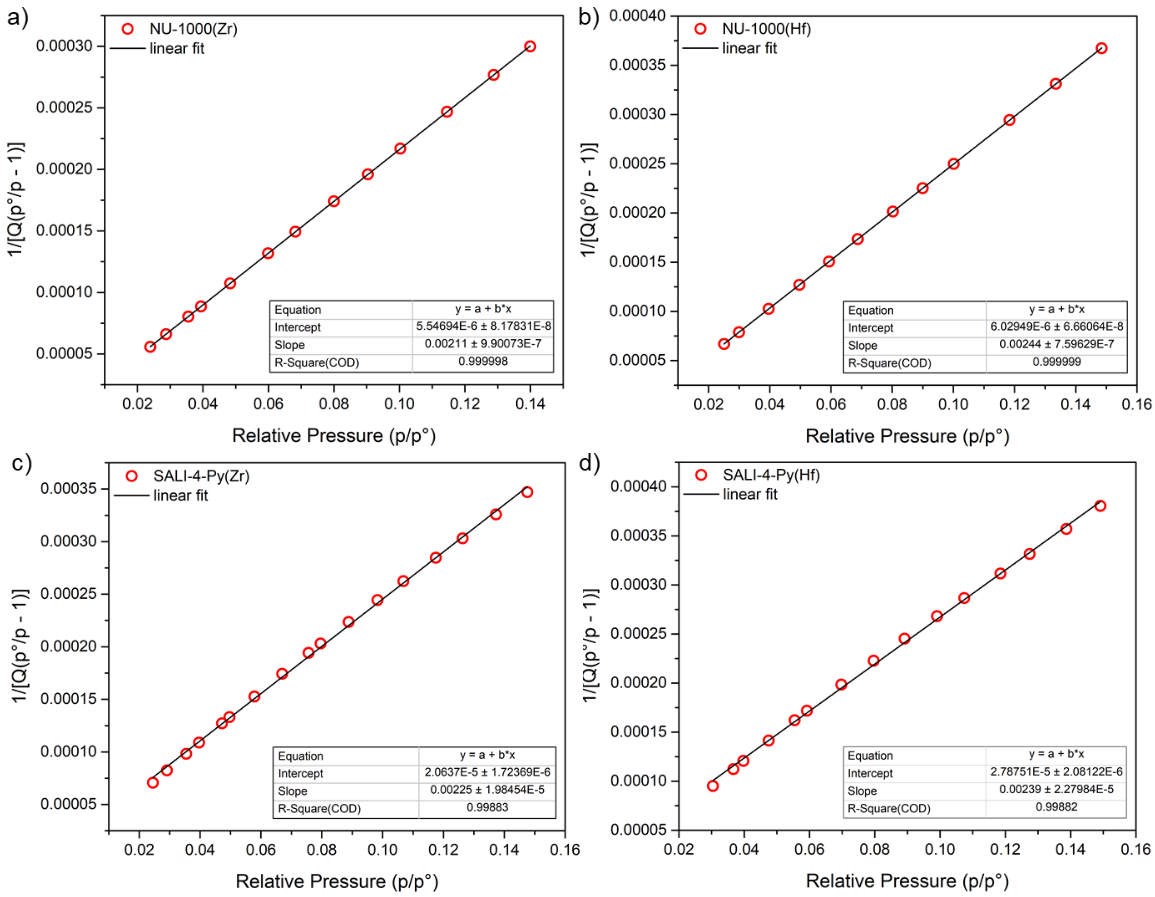

Figure S21. BET plot and fitting parameters for a-b) **NU-1000(M)**, c-d) **SALI-4-Py(M)**; M = Zr, Hf.

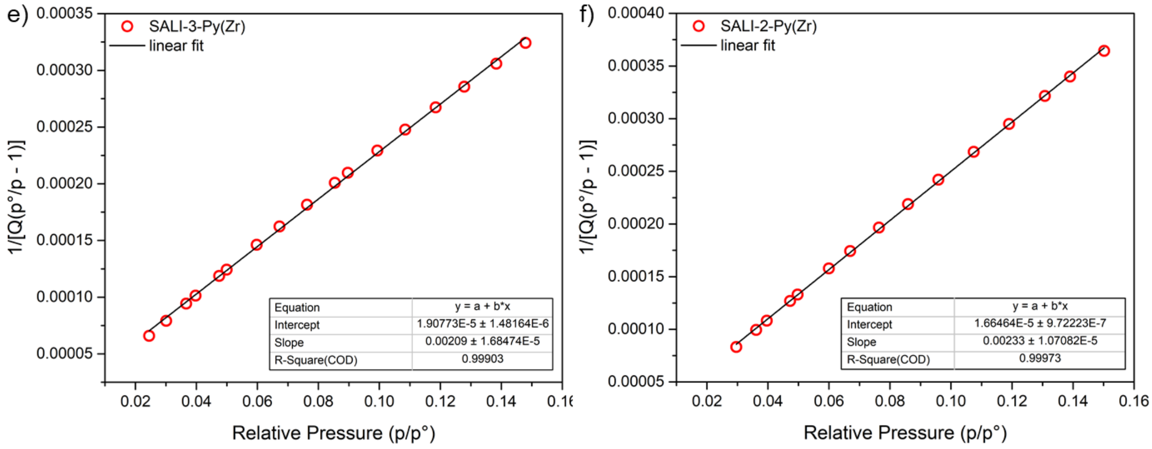

Figure S21 (continued). BET plot and fitting parameters for e) **SALI-3-Py(Zr)** and f) **SALI-2-Py(Zr)**.

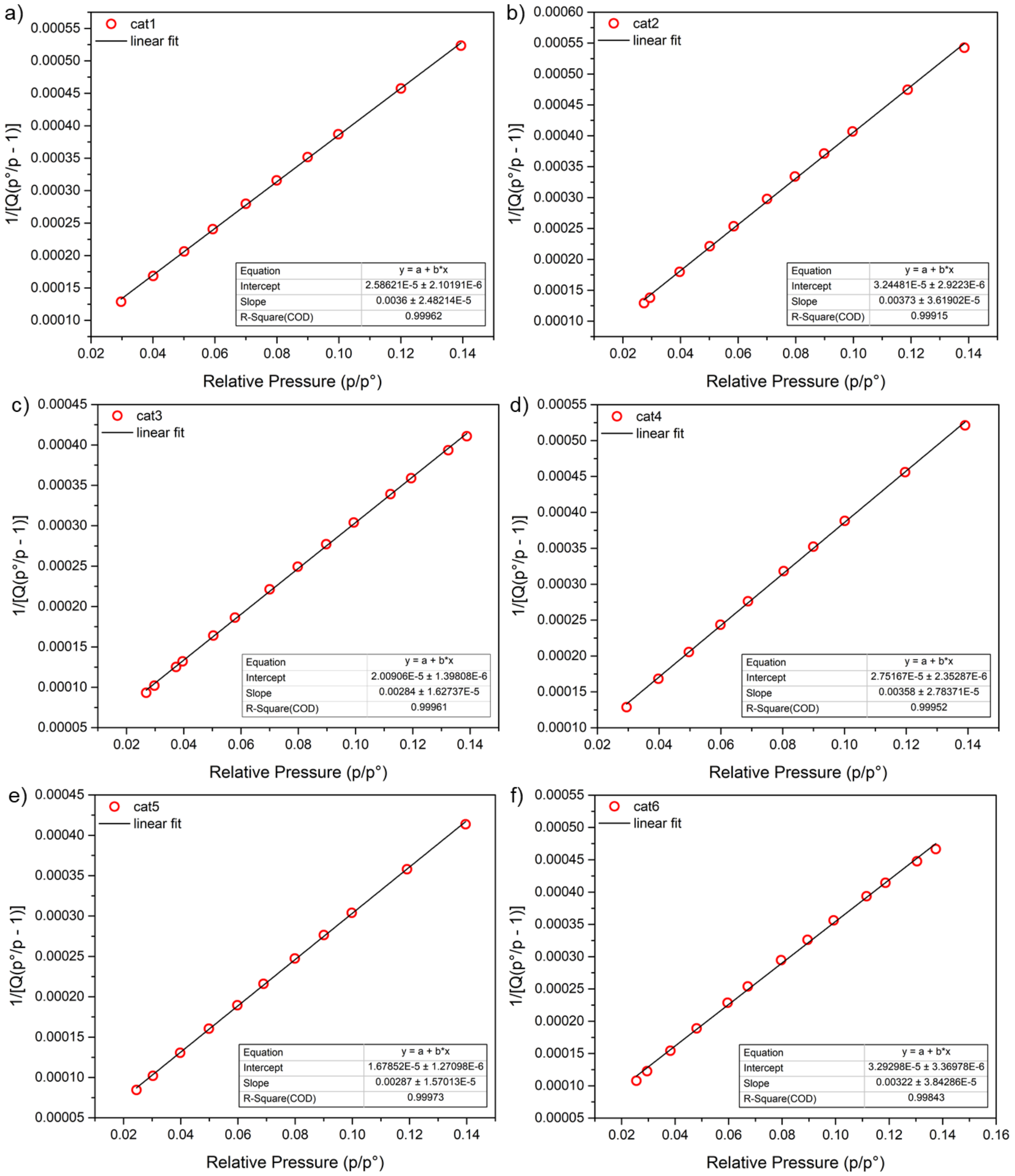

Figure S22. BET plot and fitting parameters for Zr-based *2-in-1* catalysts: a-f) **cat1–cat6**.

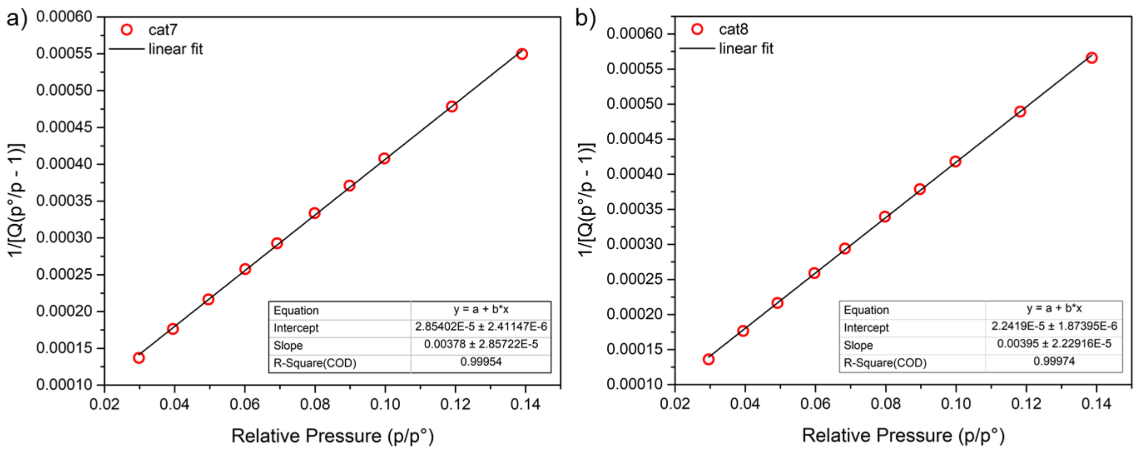

Figure S23. BET plot and fitting parameters for Hf-based *2-in-1* catalyst: a) **cat7** and b) **cat8**.

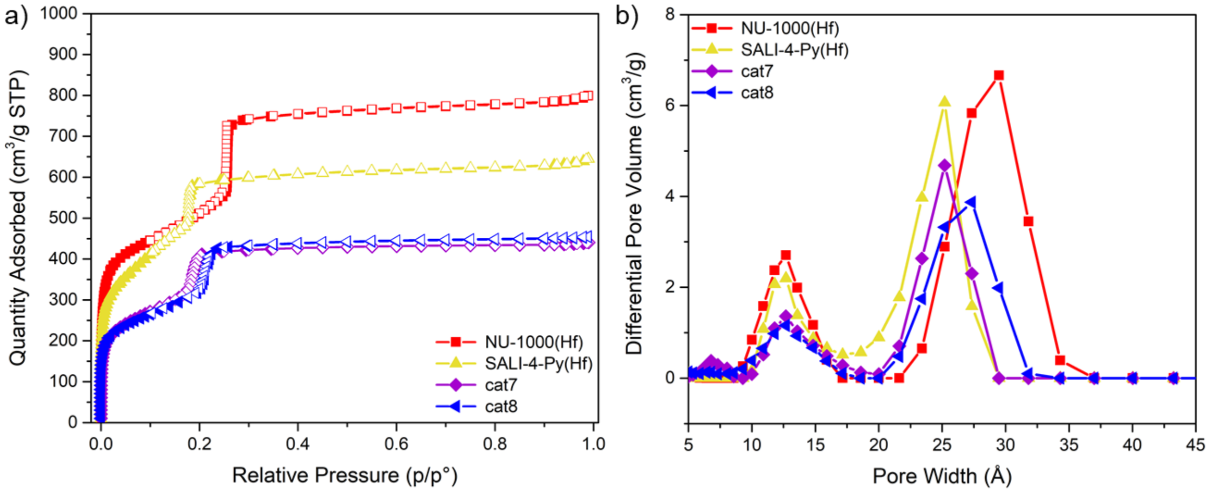

Figure S24. a) Nitrogen isotherms measured at 77 K in selected Hf-based *2-in-1* catalysts **cat7** and **cat8** compared with **NU-1000(Hf)** before and after SALI reaction (filled symbols - adsorption, open symbols - desorption). b) Density functional theory (DFT) pore size distribution (PSD) of selected Zr-MOFs before and after functionalization.

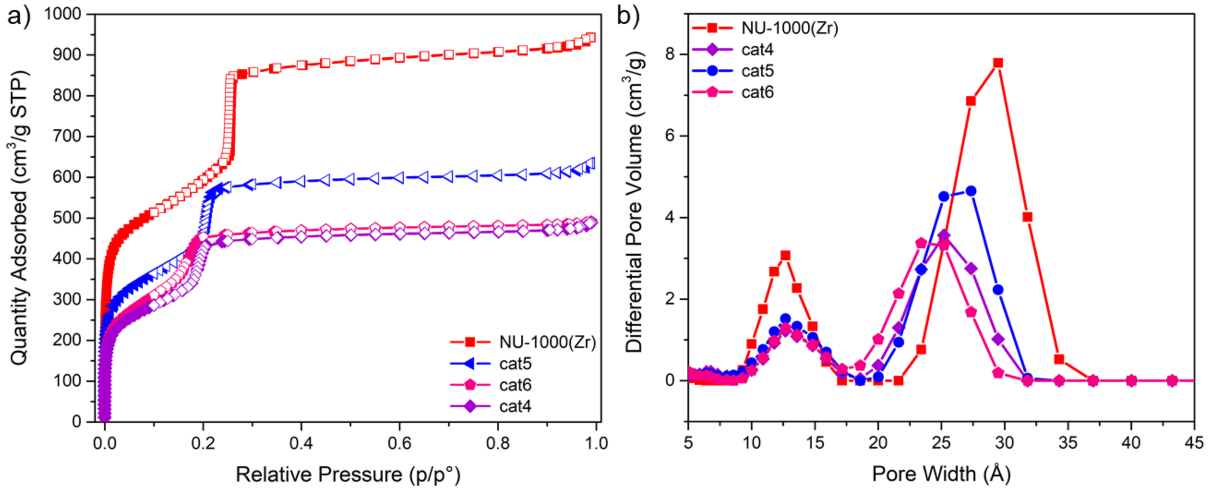

Figure S25. a) Nitrogen isotherms measured at 77 K in selected Zr-based *2-in-1* catalysts **cat4–cat6** (filled symbols - adsorption, open symbols - desorption). b) Density functional theory (DFT) pore size distribution (PSD) of selected Zr-MOFs before and after functionalization.

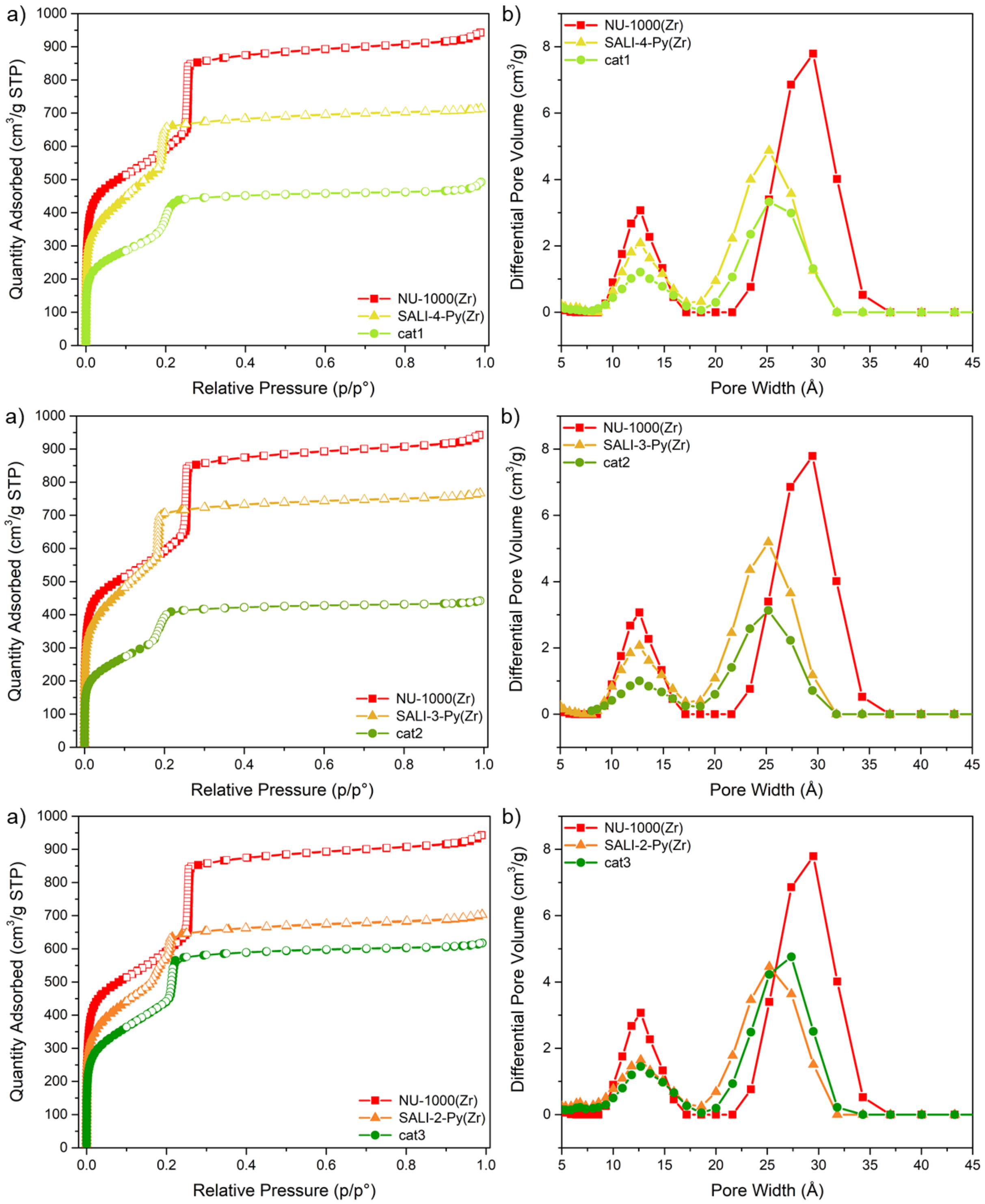

Figure S26. a) Nitrogen isotherms measured at 77 K in selected Zr-based materials (filled symbols - adsorption, open symbols - desorption). b) Density functional theory (DFT) pore size distribution (PSD) of selected Zr-MOFs before and after functionalization.

S4.6. Carbon dioxide sorption studies and isosteric heats of CO_2 adsorption (Q_{st})

The CO_2 sorption studies in **cat1, cat4–cat8** samples result in Langmuir-type isotherms. The measurements were carried out at 273, 283 and 293 K up to 1.0 bar. The isosteric heats of adsorption (Q_{st}) were calculated by fitting the CO_2 adsorption isotherms using the dual-site Langmuir model and Clausius-Clapeyron equation.[8]

The measured CO_2 isotherms at 273, 283 and 293 K were fit with the dual-site Langmuir model given by equation 1 with two distinct adsorption sites *A* and *B*:

$$q \equiv q_A + q_B = \frac{q_{sat,A} \cdot b_A \cdot p}{1 + b_A \cdot p} + \frac{q_{sat,B} \cdot b_B \cdot p}{1 + b_B \cdot p} \qquad (1)$$

where q is the total gravimetric uptake of CO_2 (mmol/g) at pressure p, $q_{sat,i}$ and b_i are the saturation loading and the Langmuir affinity parameters for site i, respectively.

The temperature dependence of the Langmuir constant b_i is described by equation 2:

$$b_i = b_{i0} \cdot exp\left(\frac{E_i}{RT}\right) \qquad (2)$$

where E_i is the heat of the adsorption for site i, b_{i0} is the pre-exponential parameter and R is the gas constant.

a)

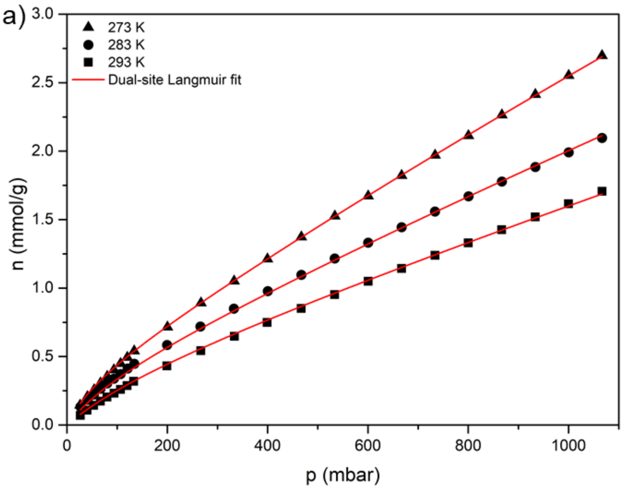

b)

Parameter	cat1
$q_{sat,A}$ (mol/kg)	0.33
$q_{sat,B}$ (mol/kg)	37.50
b_{A0} (Pa^{-1})	3.59×10^{-9}
b_{B0} (Pa^{-1})	1.90×10^{-8}
E_A (kJ/mol)	34.66
E_B (kJ/mol)	18.42
Q_{st} (kJ/mol)	28.5 - 19.6

Figure S27. a) CO_2 isotherms for **cat1**. b) Dual-site Langmuir parameters for CO_2 adsorption in **cat1**. These parameters were determined by fitting CO_2 adsorption isotherms measured at 273, 283 and 293 K.

a)

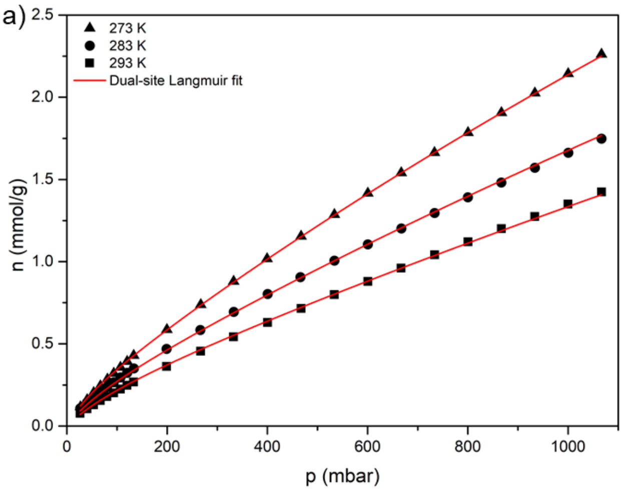

b)

Parameter	cat4
$q_{sat,A}$ (mol/kg)	0.19
$q_{sat,B}$ (mol/kg)	15.18
b_{A0} (Pa^{-1})	1.36×10^{-6}
b_{B0} (Pa^{-1})	3.06×10^{-8}
E_A (kJ/mol)	21.56
E_B (kJ/mol)	19.25
Q_{st} (kJ/mol)	20.6 - 19.3

Figure S28. a) CO_2 isotherms for **cat4**. b) Dual-site Langmuir parameters for CO_2 adsorption in **cat4**. These parameters were determined by fitting CO_2 adsorption isotherms measured at 273, 283 and 293 K.

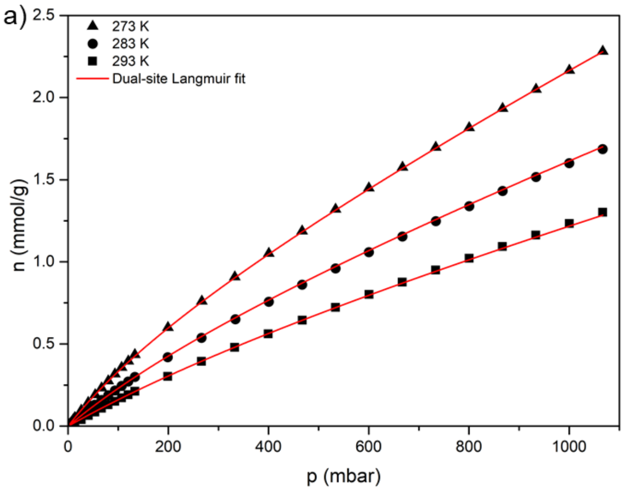

b)

Parameter	cat5
$q_{sat,A}$ (mol/kg)	0.52
$q_{sat,B}$ (mol/kg)	27.42
b_{A0} (Pa^{-1})	2.39×10^{-9}
b_{B0} (Pa^{-1})	2.79×10^{-9}
E_A (kJ/mol)	32.53
E_B (kJ/mol)	22.94
Q_{st} (kJ/mol)	27.6 - 24.4

Figure S29. a) CO_2 isotherms for **cat5**. b) Dual-site Langmuir parameters for CO_2 adsorption in **cat5**. These parameters were determined by fitting CO_2 adsorption isotherms measured at 273, 283 and 293 K.

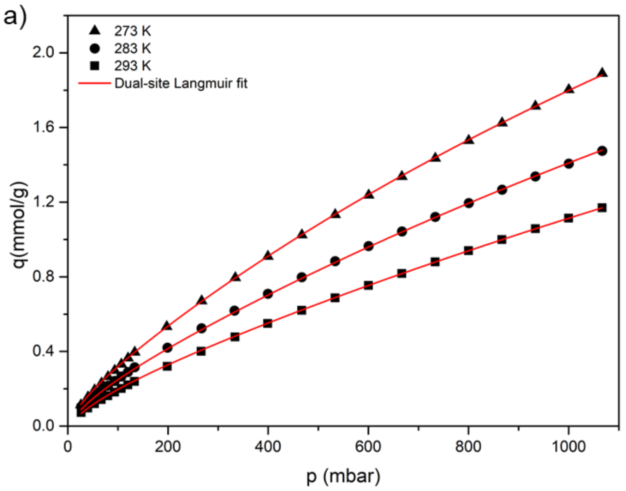

b)

Parameter	cat6
$q_{sat,A}$ (mol/kg)	6.85
$q_{sat,B}$ (mol/kg)	0.15
b_{A0} (Pa^{-1})	2.24×10^{-8}
b_{B0} (Pa^{-1})	1.16×10^{-6}
E_A (kJ/mol)	21.70
E_B (kJ/mol)	22.65
Q_{st} (kJ/mol)	22.3 - 21.7

Figure S30. a) CO_2 isotherms for **cat6**. b) Dual-site Langmuir parameters for CO_2 adsorption in **cat6**. These parameters were determined by fitting CO_2 adsorption isotherms measured at 273, 283 and 293 K.

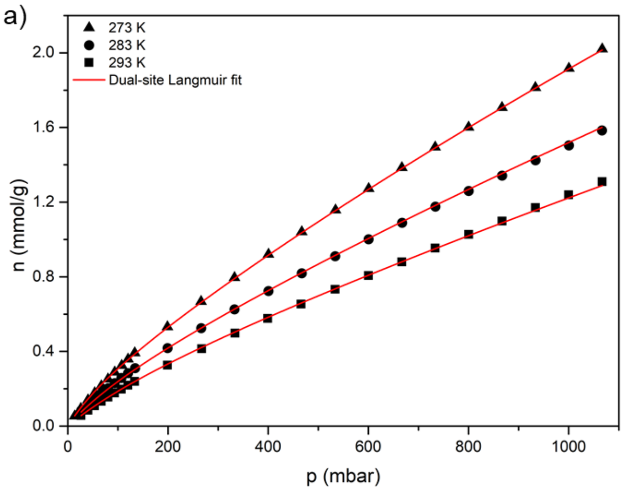

b)

Parameter	cat7
$q_{sat,A}$ (mol/kg)	15.22
$q_{sat,B}$ (mol/kg)	0.23
b_{A0} (Pa^{-1})	3.99×10^{-8}
b_{B0} (Pa^{-1})	1.98×10^{-7}
E_A (kJ/mol)	18.30
E_B (kJ/mol)	24.80
Q_{st} (kJ/mol)	21.8 - 18.6

Figure S31. a) CO_2 isotherms for **cat7**. b) Dual-site Langmuir parameters for CO_2 adsorption in **cat7**. These parameters were determined by fitting CO_2 adsorption isotherms measured at 273, 283 and 293 K.

a)

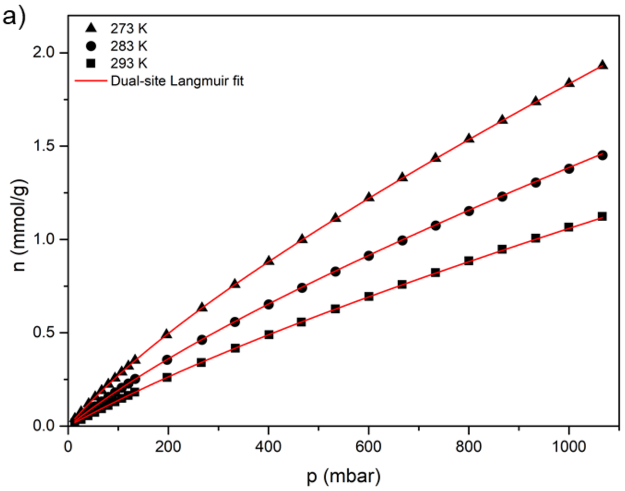

b)

Parameter	cat8
$q_{sat,A}$ (mol/kg)	22.95
$q_{sat,B}$ (mol/kg)	0.48
b_{A0} (Pa^{-1})	4.17×10^{-9}
b_{B0} (Pa^{-1})	1.35×10^{-8}
E_A (kJ/mol)	22.02
E_B (kJ/mol)	28.07
Q_{st} (kJ/mol)	24.9 - 22.9

Figure S32. a) CO_2 isotherms for **cat8**. b) Dual-site Langmuir parameters for CO_2 adsorption in **cat8**. These parameters were determined by fitting CO_2 adsorption isotherms measured at 273, 283 and 293 K.

a)

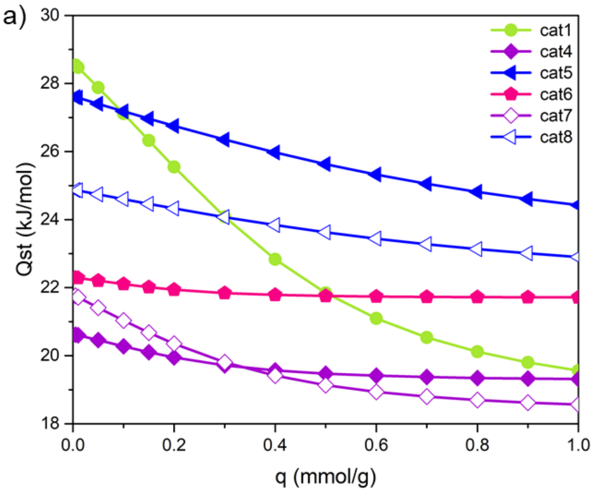

b)

	Qst (kJ/mol)[1]
**cat1**	28.5 - 19.6
**cat4**	20.6 - 19.3
**cat5**	27.6 - 24.4
**cat6**	22.3 - 21.7
**cat7**	21.8 - 18.6
**cat8**	24.9 - 22.9

[1] based on dual-site Langmuir (DSL) model

Figure S33. a) CO_2 isosteric heat of adsorption plots for representative Zr- and Hf-based catalysts. b) Calculated isosteric heats of adsorption (Q_{st}) of CO_2 in selected MOF-based *2-in-1* catalysts.

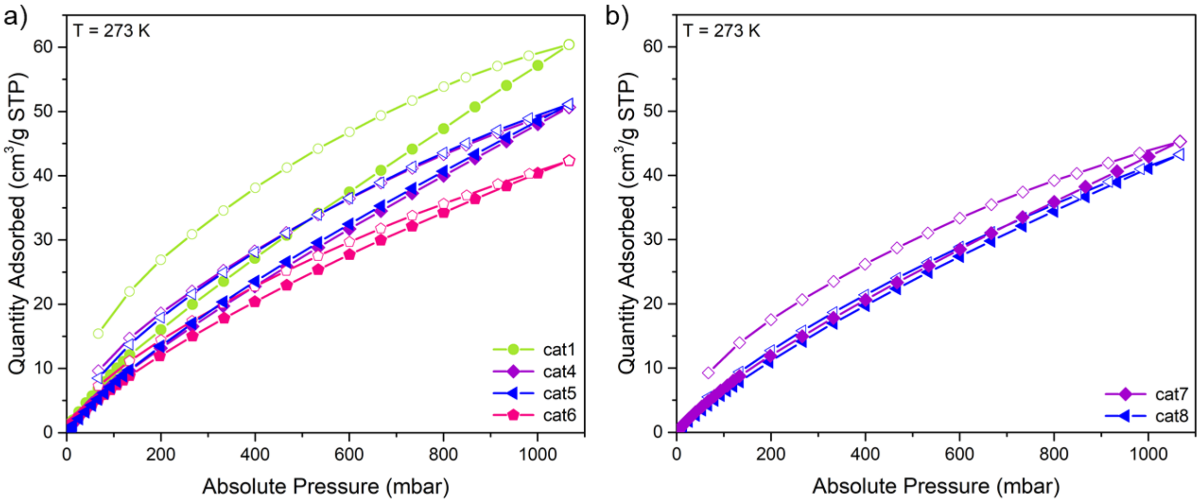

Figure S34. CO_2 isotherms at 273 K for representative *2-in-1* catalytic systems: a) Zr-based **cat1, cat4–cat6** and b) Hf-based **cat7** and **cat8**. Filled and open symbols represent adsorption and desorption, respectively.

S4.6. TGA-DTG analysis

All performed TGA measurements were conducted in the range of 30-1000 °C in oxidative conditions (O_2/N_2 = 20/80) resulting in only metal oxide (MO_2, where M = Zr, Hf) remaining as a final product (originating from inorganic node of analyzed material).[9] Similar weight losses (less than 10%) can be observed at the beginning of the TGA curves and are associated with the removal of the adsorbed nonbonded solvent molecules (up to ca. 100 °C) and further removal of coordinated monocarboxylates and dehydroxylation of the metal nodes (up to 300 °C).[9] The highest weight loss is caused by the decomposition of the framework which occurs in the temperature range of ca. 400-500 °C. For **cat1–cat8** samples obtained after the alkylation reaction we observed an additional significant weight loss appearing in the range of 150-300 °C which we determined to be a result of the removal of haloalkane molecules (CH_3I, C_4H_9I, C_4H_9Br, $C_6H_4F_9I$). The weight losses calculated from TGA profiles were compared with the theoretical values (based on the material composition determined in ^{1}H NMR studies) where the ZrO_2 or HfO_2 were assumed to be the final product of thermal decomposition.[9] For example, in TGA profile of **cat4** we recorded two weight losses of 22.2 wt% and 54.0 wt%, which are in a good agreement with the theoretical values of 22.6 wt% and 54.8 wt%, corresponding respectively to the removal of butyl iodide and combined loss of 4-$PyCO_2^-$ ligand and $TBAPy^{4-}$ linker.

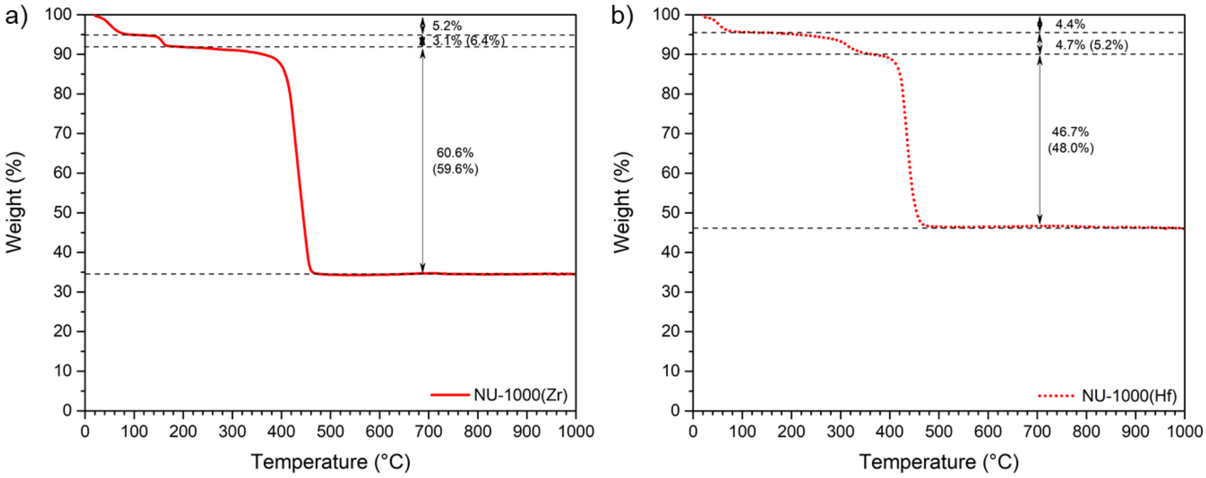

Figure S35. TGA profile of a) **NU-1000(Zr)** and b) **NU-1000(Hf)**. The values in the parentheses are calculated theoretical weight losses based on the chemical formula of selected MOFs.

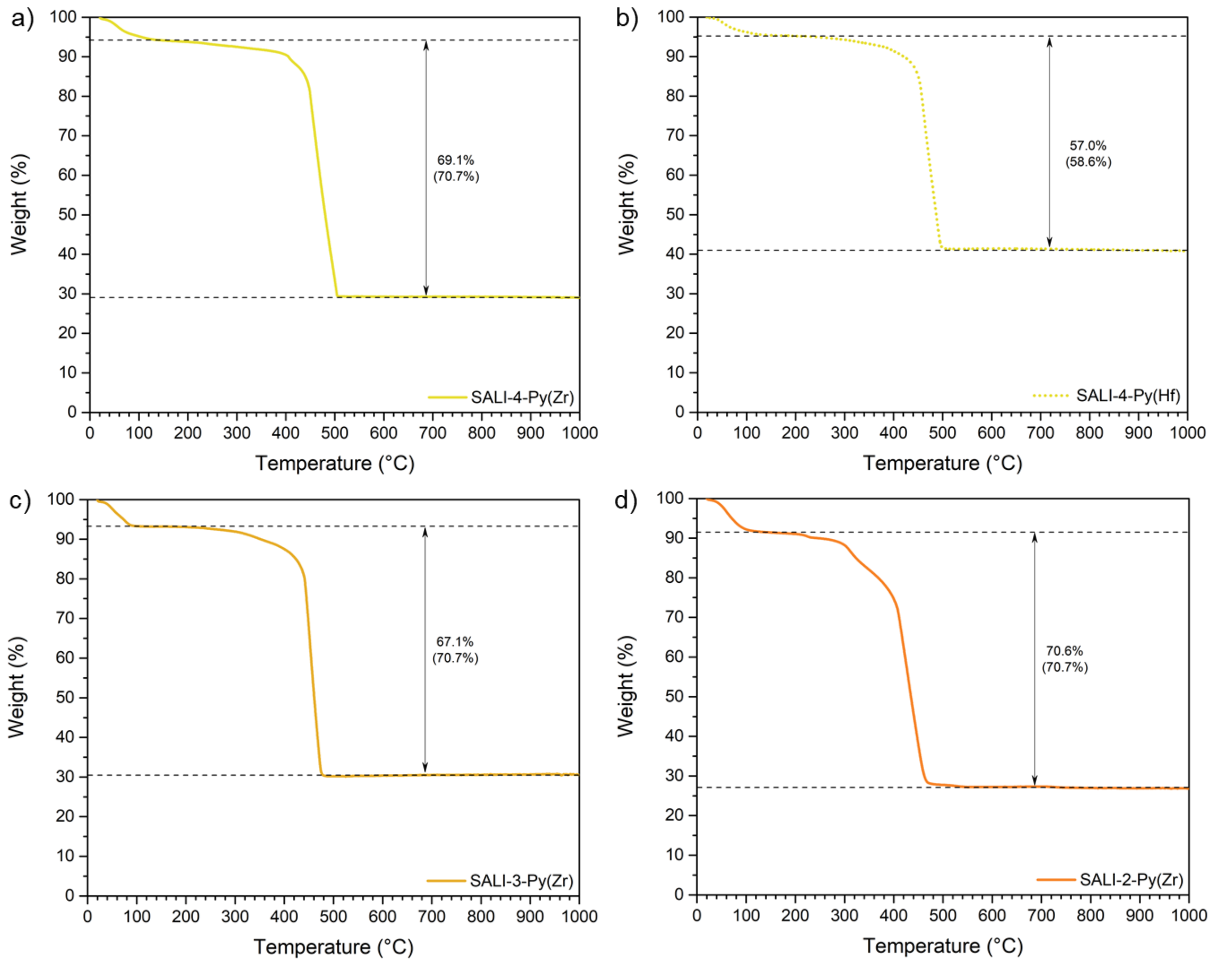

Figure S36. TGA profile of NU-1000(M) after SALI functionalization with pyridinecarboxylates: a) **SALI-4-Py(Zr)**, b) **SALI-4-Py(Hf)**, c) **SALI-3-Py(Zr)** and d) **SALI-2-Py(Zr)**. The values in the parentheses are calculated theoretical weight losses based on the chemical formula of selected MOFs.

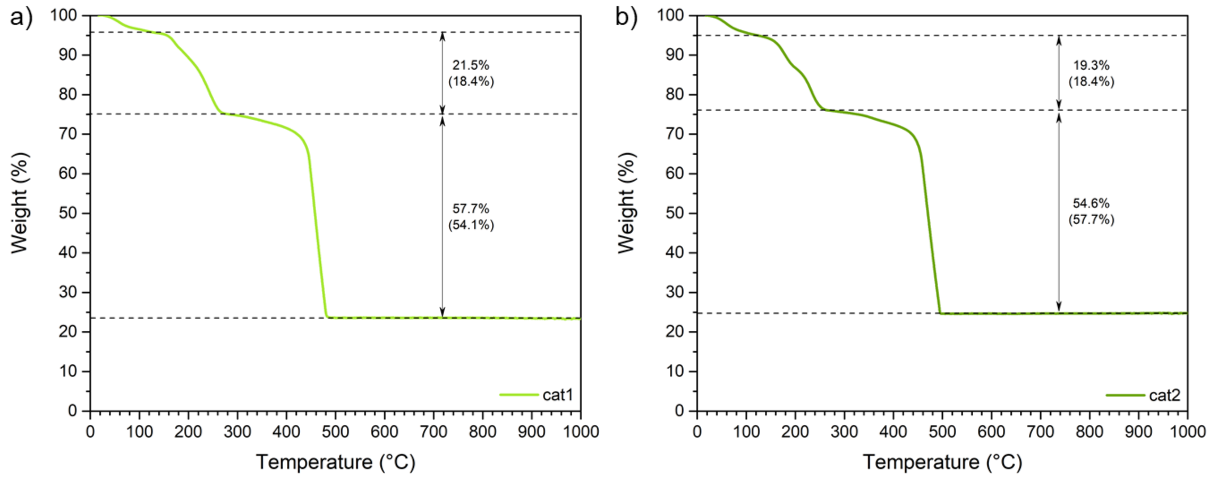

Figure S37. TGA profile of *2-in-1* catalytic systems: a) **cat1** and b) **cat2**. The values in the parentheses are calculated theoretical weight losses based on the chemical formula of selected MOFs.

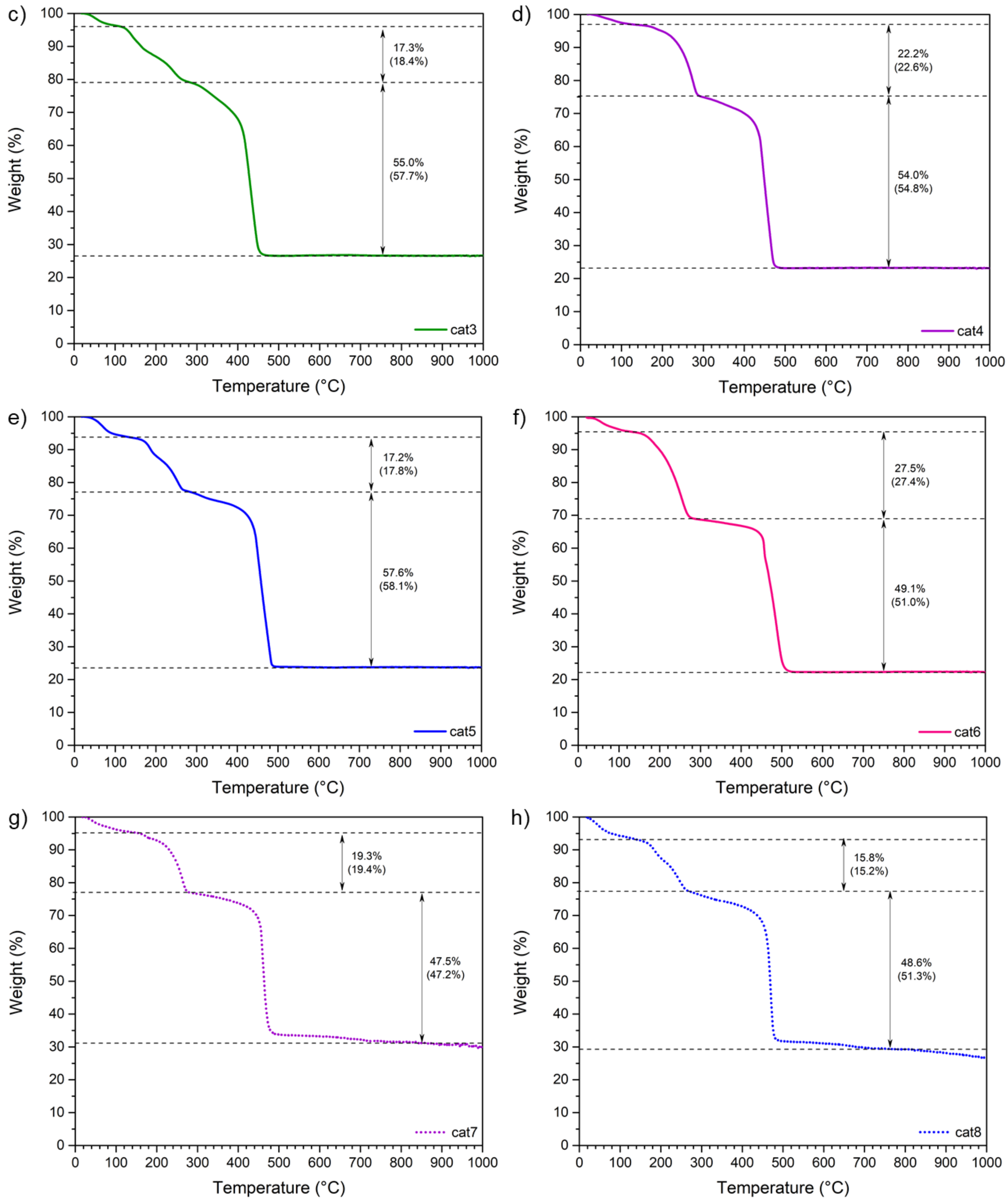

Figure S37 (continued). TGA profiles of *2-in-1* catalytic systems: c-h) **cat3–cat8**. The values in the parentheses are calculated theoretical weight losses based on the chemical formula of selected MOFs.

S4.7. SEM images and EDS analysis

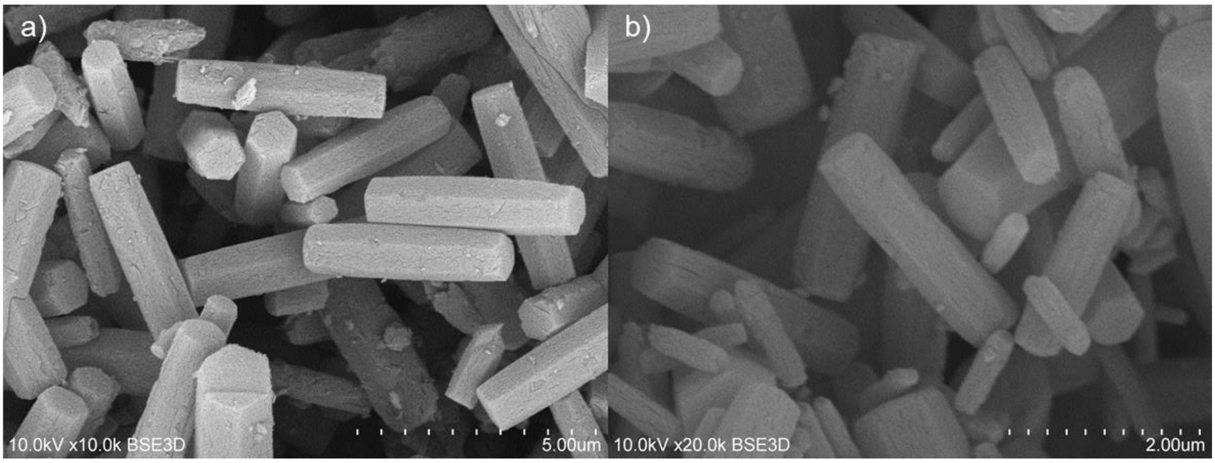

Figure S38. SEM images of a) **NU-1000(Zr)** and b) **NU-1000(Hf)**.

Table S2. EDS analysis of atomic weights of selected elements in **cat1–8** materials.

#	sample	M (=Zr, Hf)	I (or Br)	M/I (or M/Br)	
		atom%	atom%	experimental	theoretical[2]
1	**cat1**	1.51	1	1.51	1.5
2	**cat2**	1.34	1.34	1	
3	**cat3**	1.59	0.73	2.17	
4	**cat4**	2.40	1.75	1.37	
5	**cat5**	1.44	0.92	1.56	
6	**cat6**	1.57	1.12	1.40	
7	**cat7**	5.06	3.69	1.37	
8	**cat8**	3.72	2.05	1.81	

[1] based on data collected in EDS analysis

[2] calculated assuming complete two-step post-synthetic functionalization of **NU-1000(Zr)** resulting in materials with general chemical formula: $Zr_6O_4(\mu_3\text{-}OH)_4(TBAPy^-)_2(RX\text{-}n\text{-}COO_2^-)_4$, where RX = CH_3I, C_4H_9I, C_4H_9Br, $C_6H_4F_9I$, n = 2,3,4.

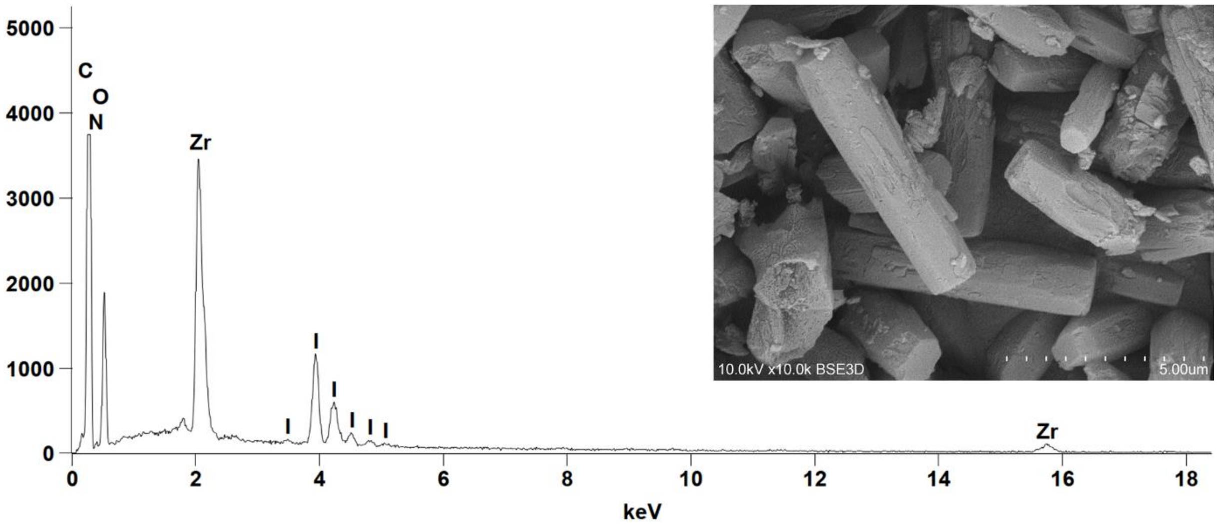

Figure S39. EDS spectrum of **cat1**. SEM image of **cat1** as an inset.

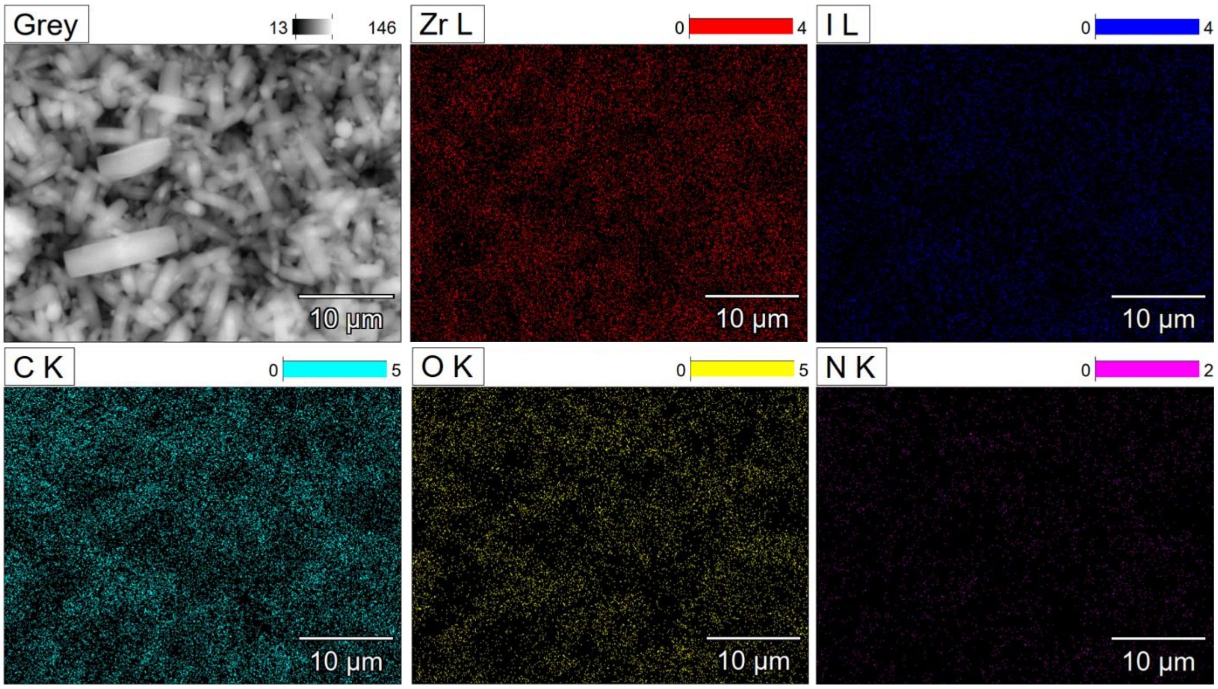

Figure S40. EDS mapping of **cat1**.

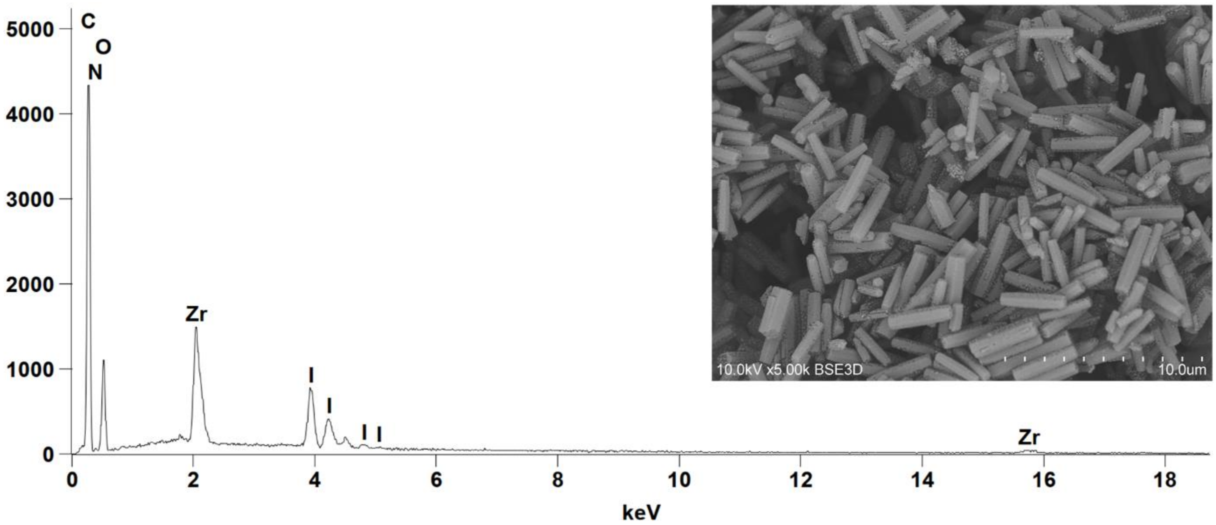

Figure S41. EDS spectrum of **cat2**. SEM image of **cat2** as an inset.

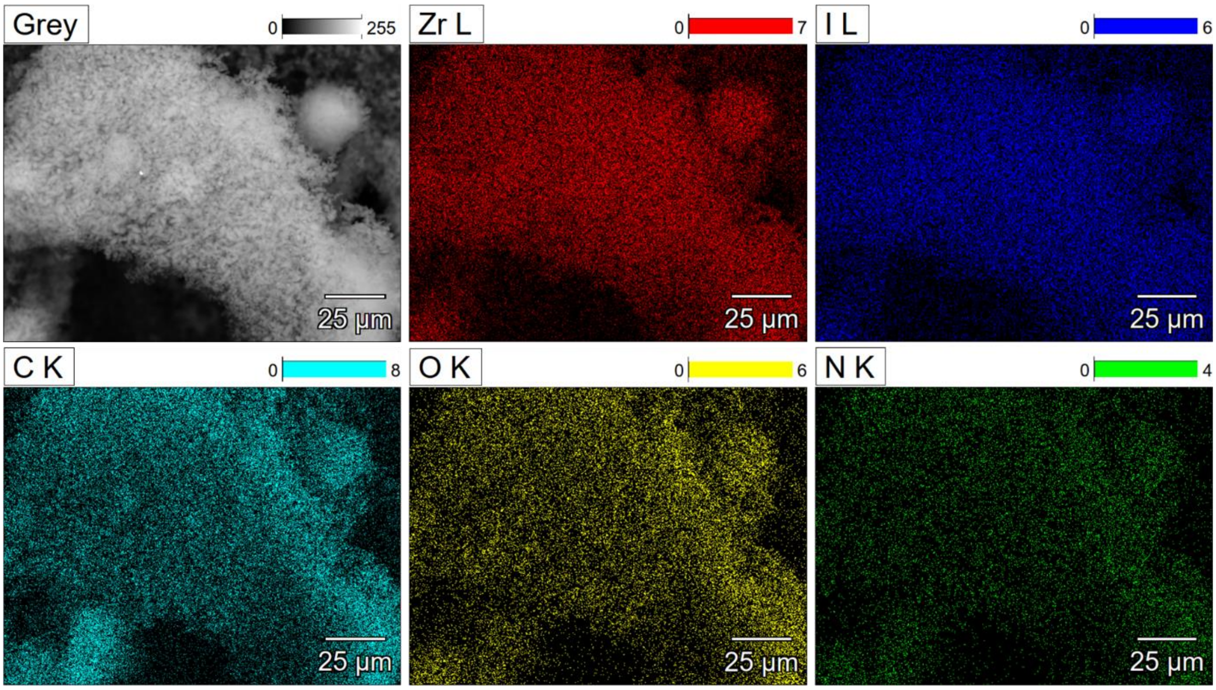

Figure S42. EDS mapping of **cat2**.

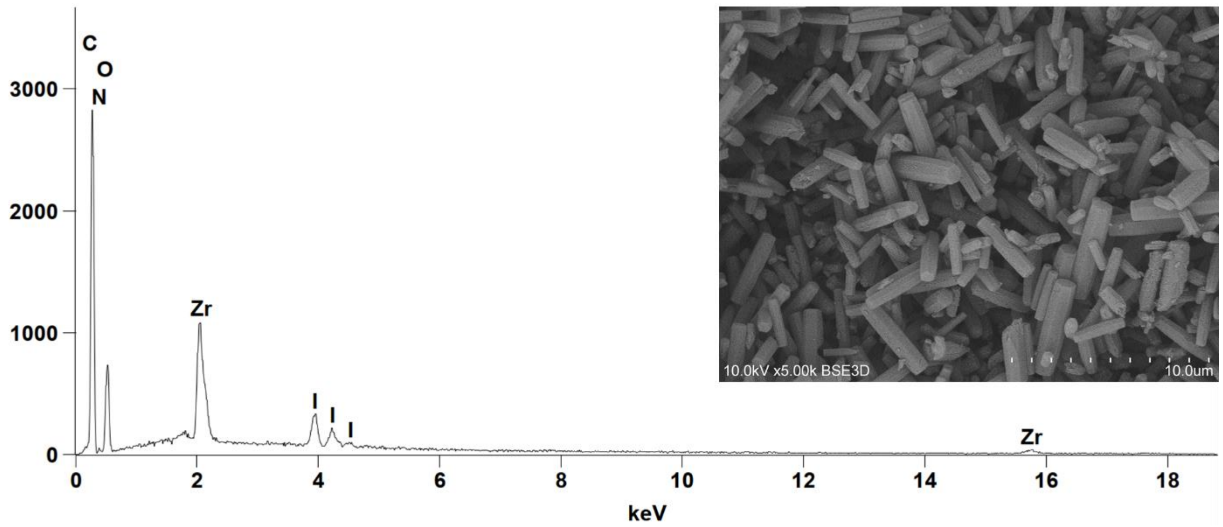

Figure S43. EDS spectrum of **cat3**. SEM image of **cat3** as an inset.

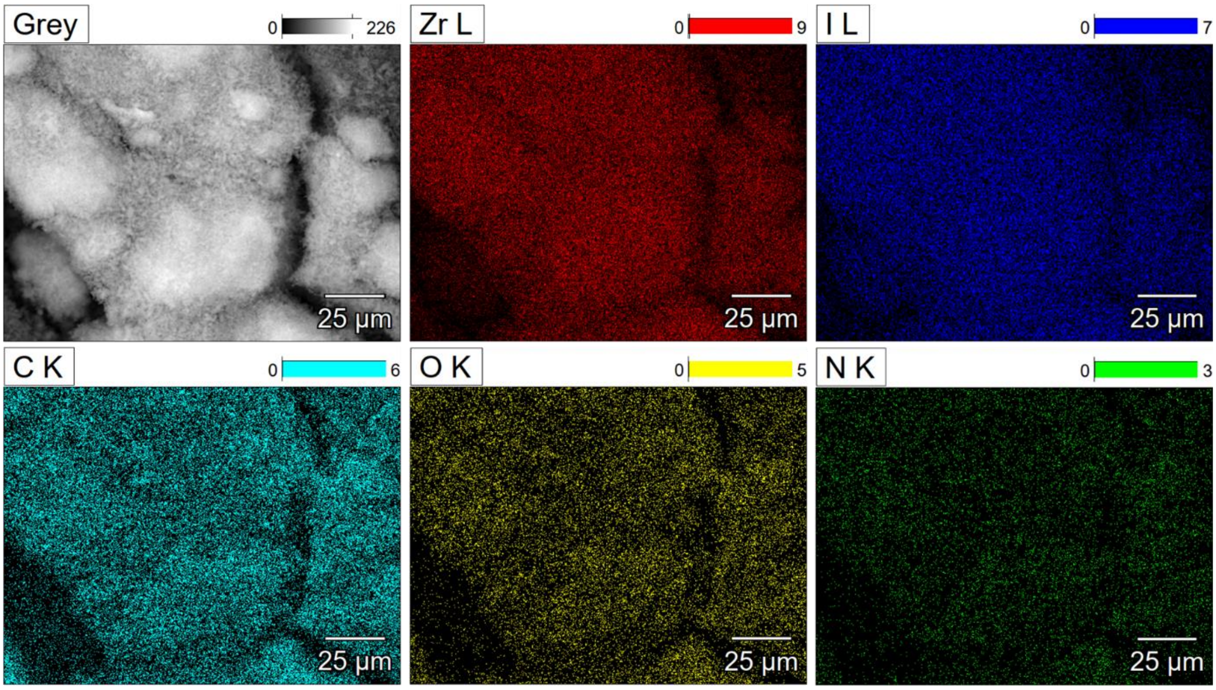

Figure S44. EDS mapping of **cat3**.

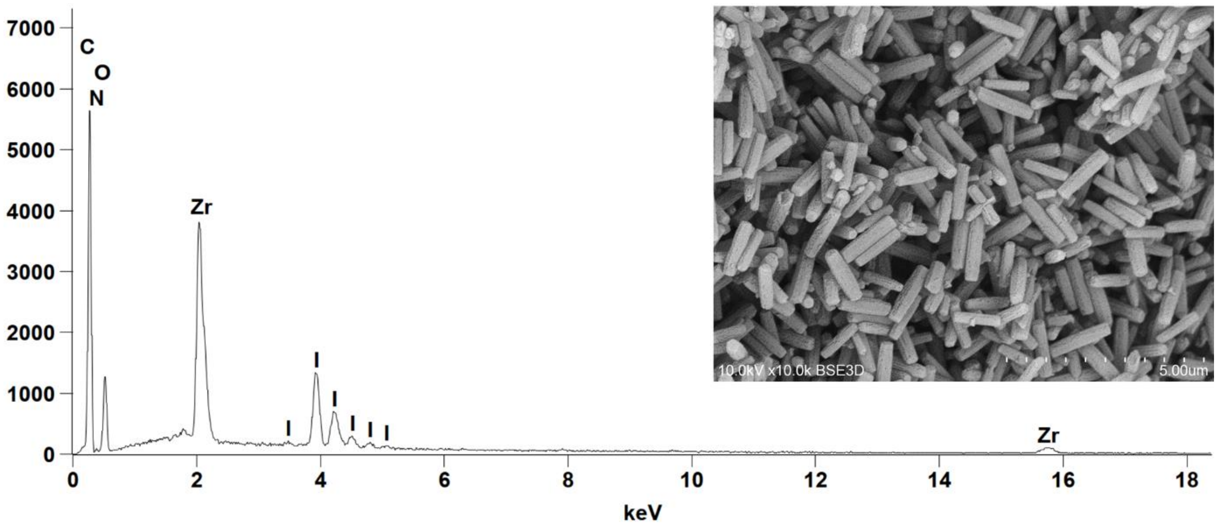

Figure S45. EDS spectrum of **cat4**. SEM image of **cat4** as an inset.

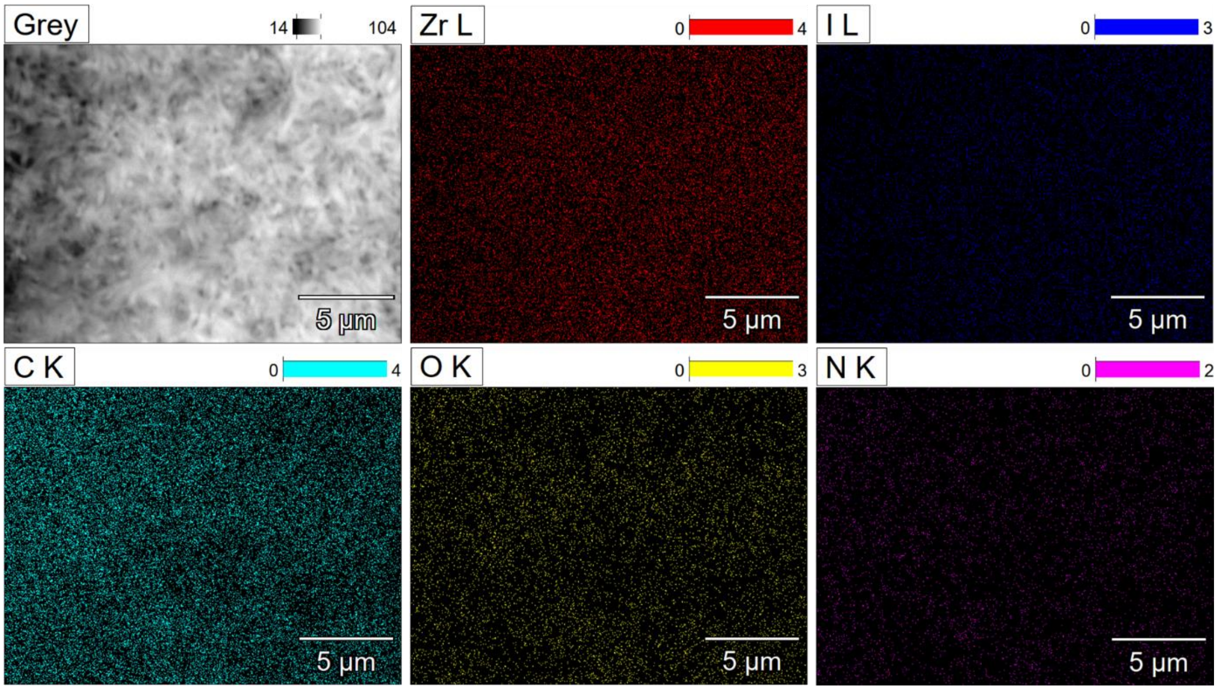

Figure S46. EDS mapping of **cat4**.

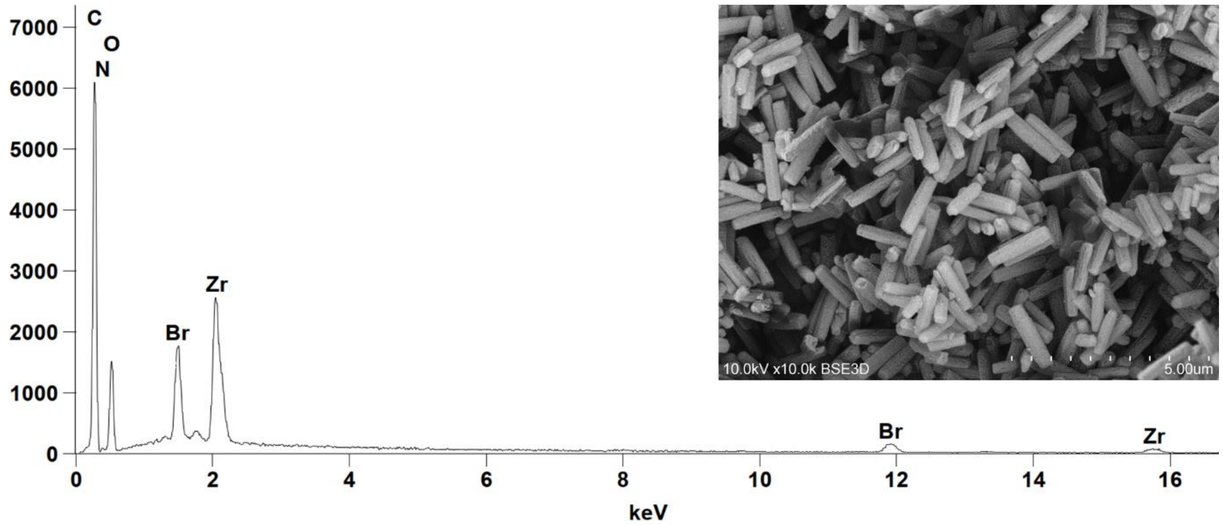

Figure S47. EDS spectrum of **cat5**. SEM image of **cat5** as an inset.

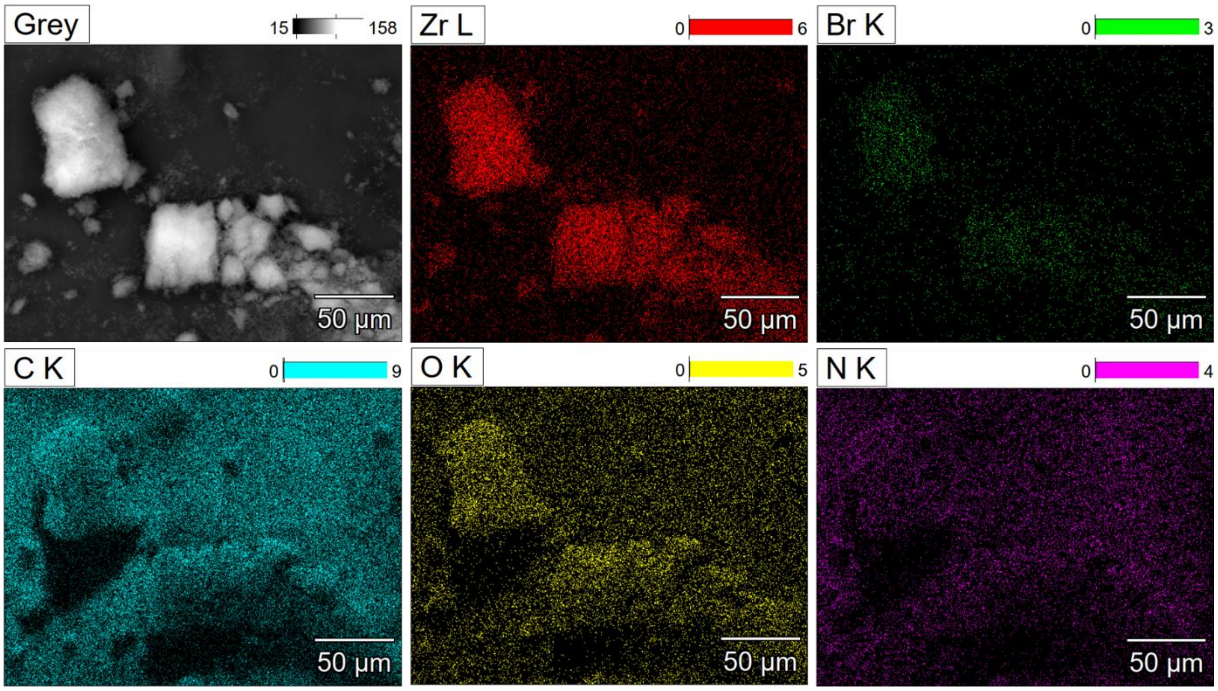

Figure S48. EDS mapping of **cat5**.

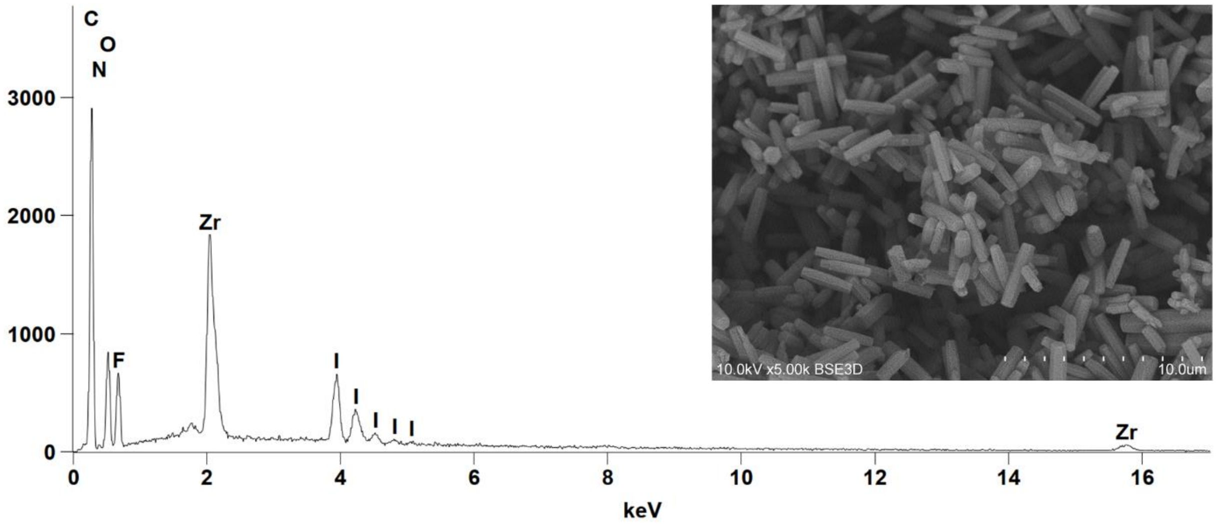

Figure S49. EDS spectrum of **cat6**. SEM image of **cat6** as an inset.

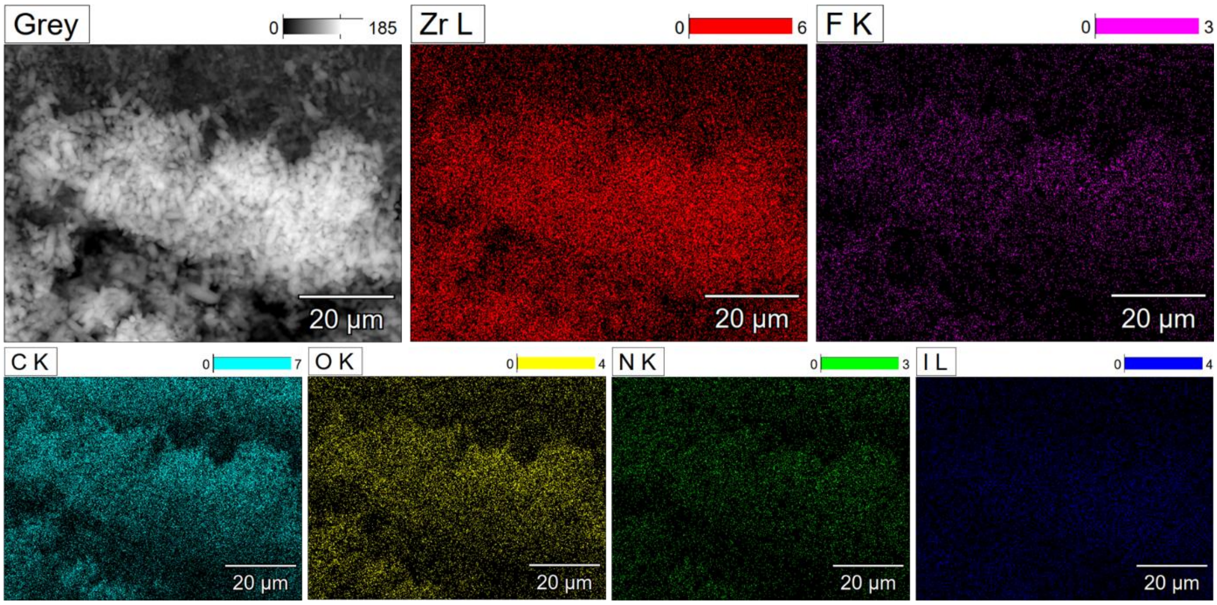

Figure S50. EDS mapping of **cat6**.

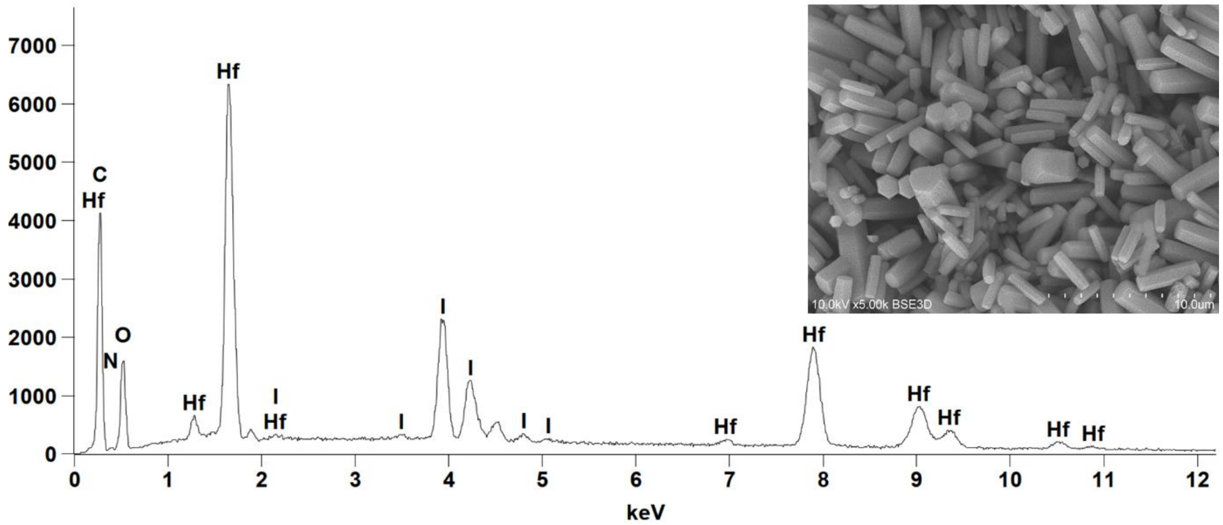

Figure S51. EDS spectrum of **cat7**. SEM image of **cat7** as an inset.

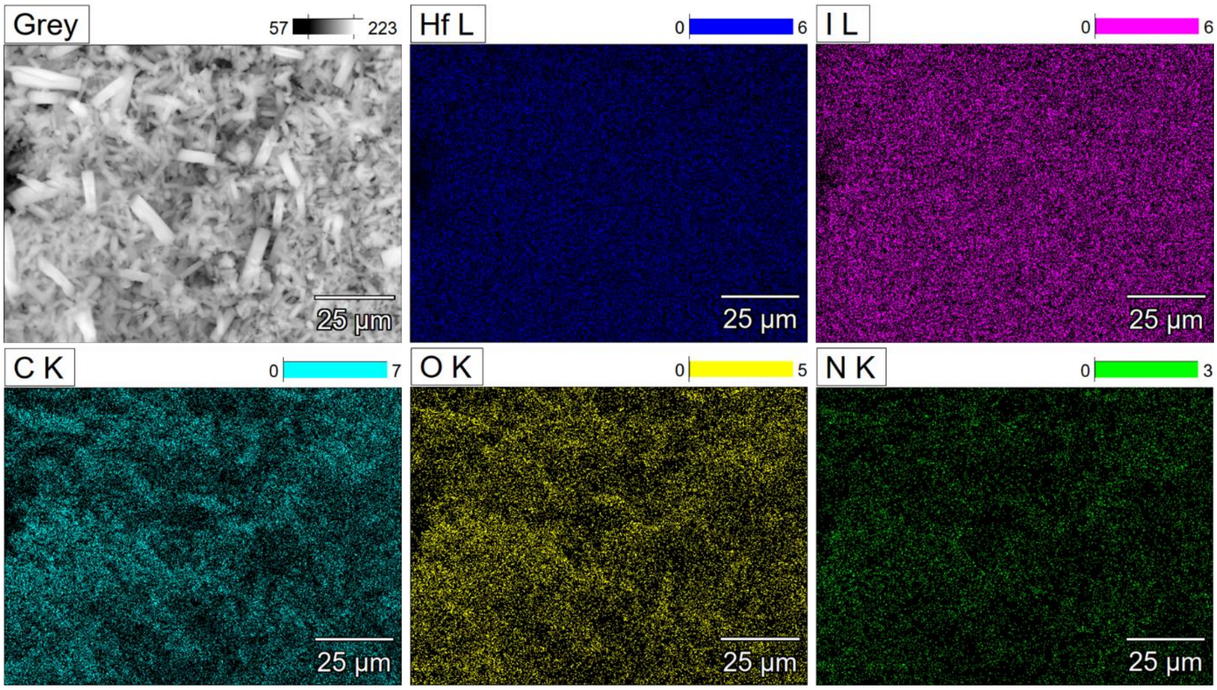

Figure S52. EDS mapping of **cat7**.

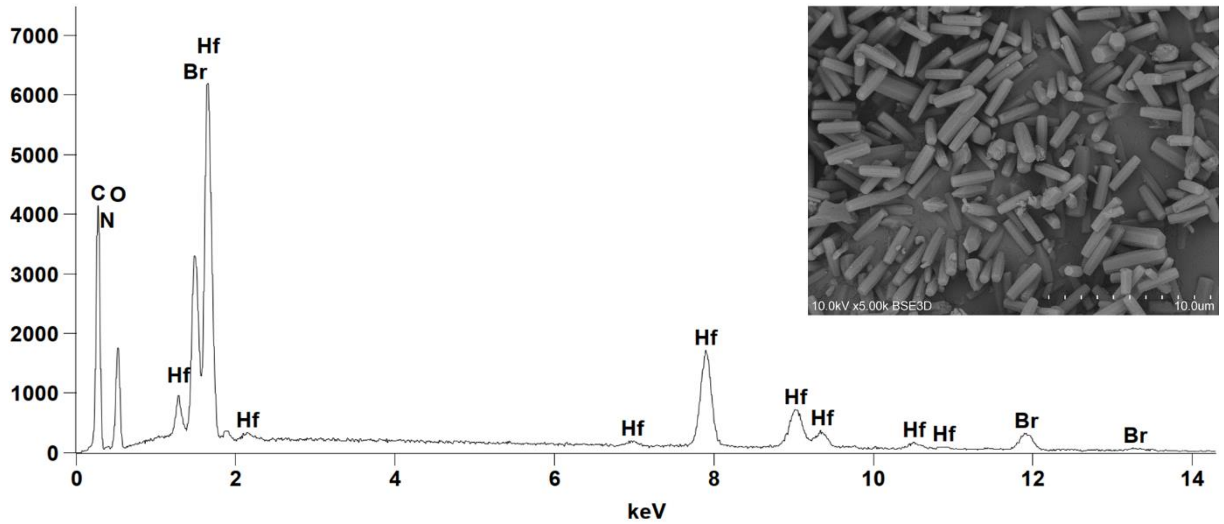

Figure S53. EDS spectrum of **cat8**. SEM image of **cat8** as an inset.

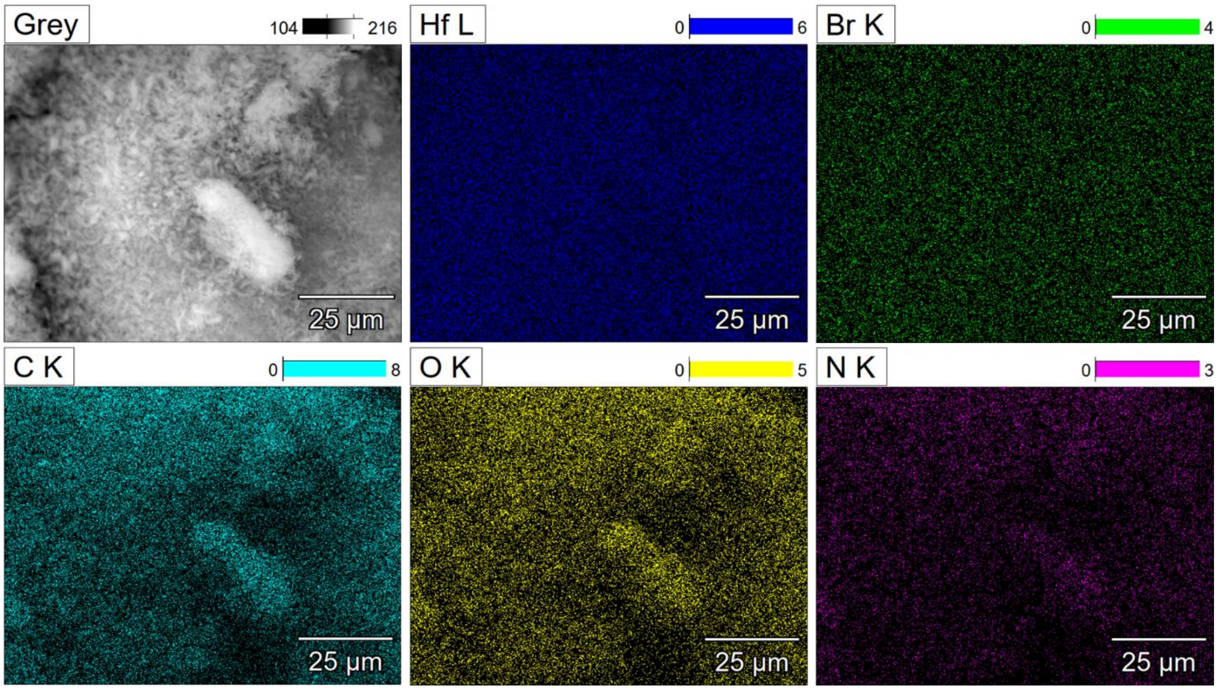

Figure S54. EDS mapping of **cat8**.

S4.8. DRIFT spectra

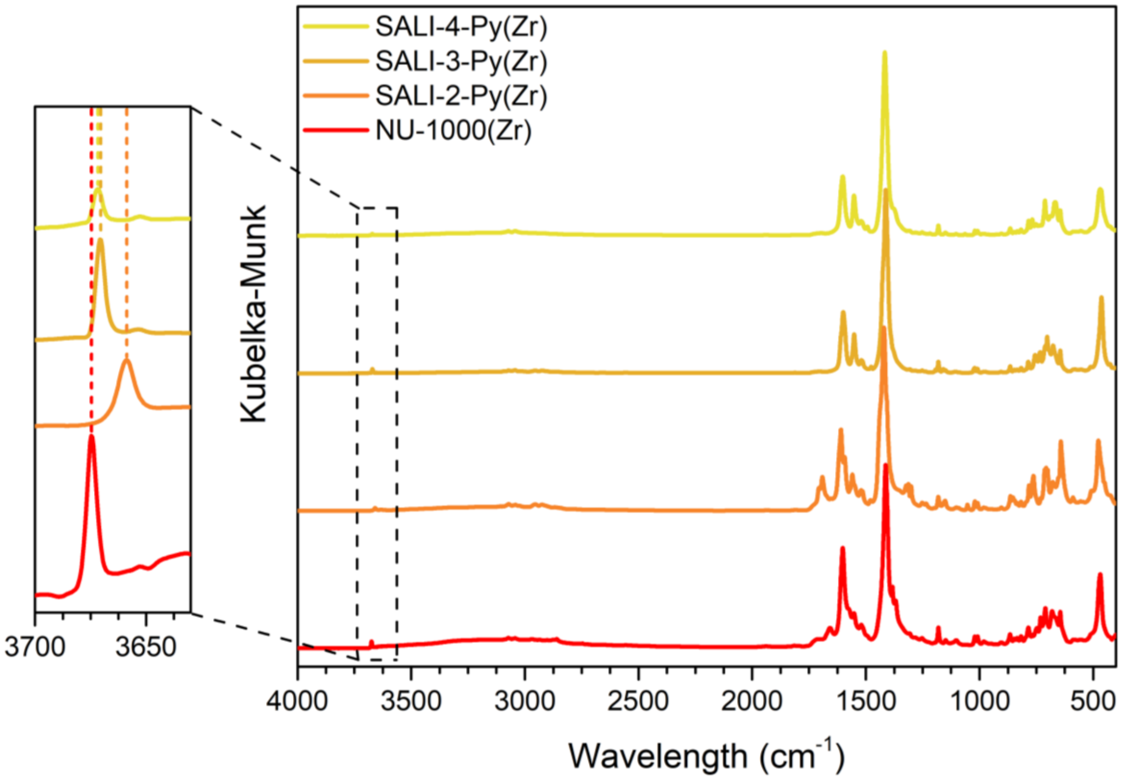

Figure S55. DRIFT spectra of **NU-1000(Zr)** before and after SALI functionalization with pyridinecarboxylates.

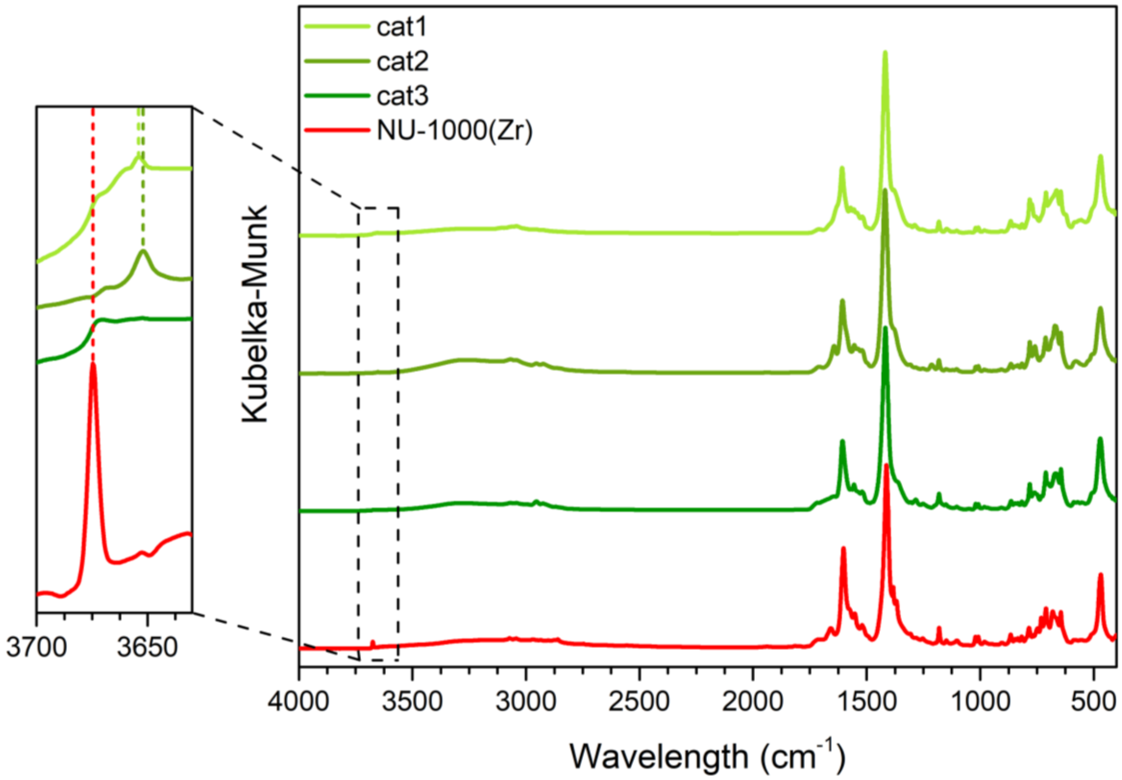

Figure S56. DRIFT spectra of **cat1–cat3**.

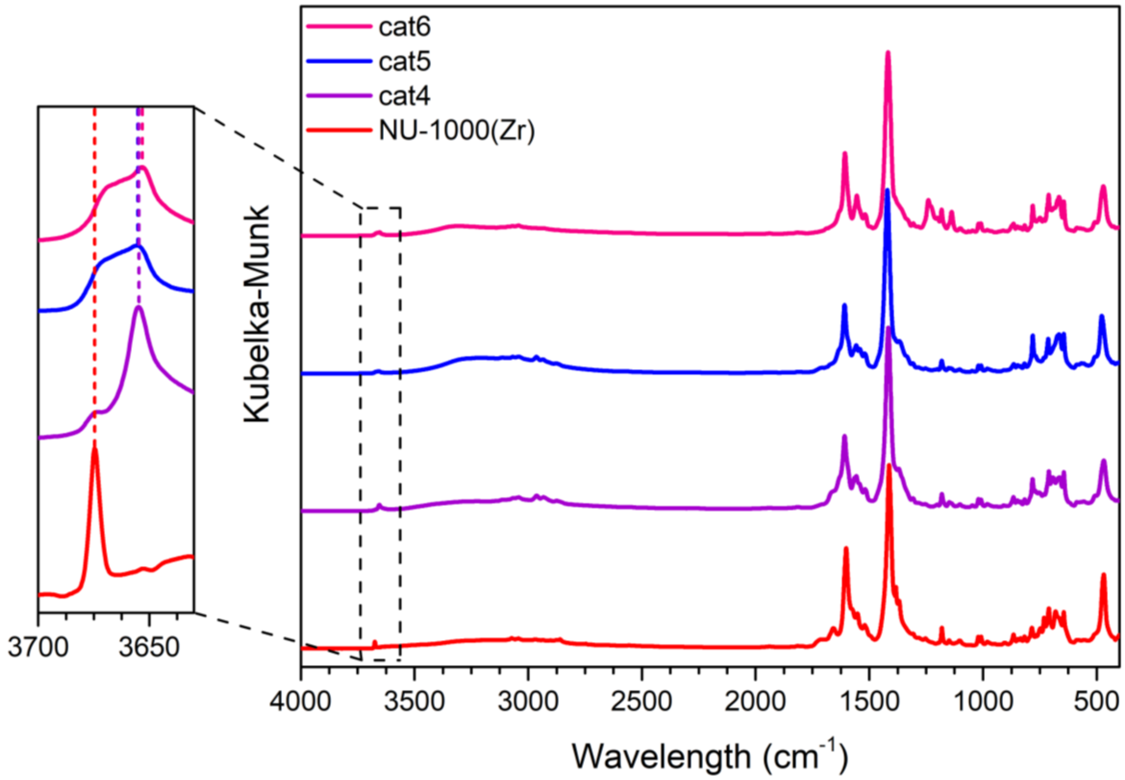

Figure S57. DRIFT spectra of **cat4–cat6**.

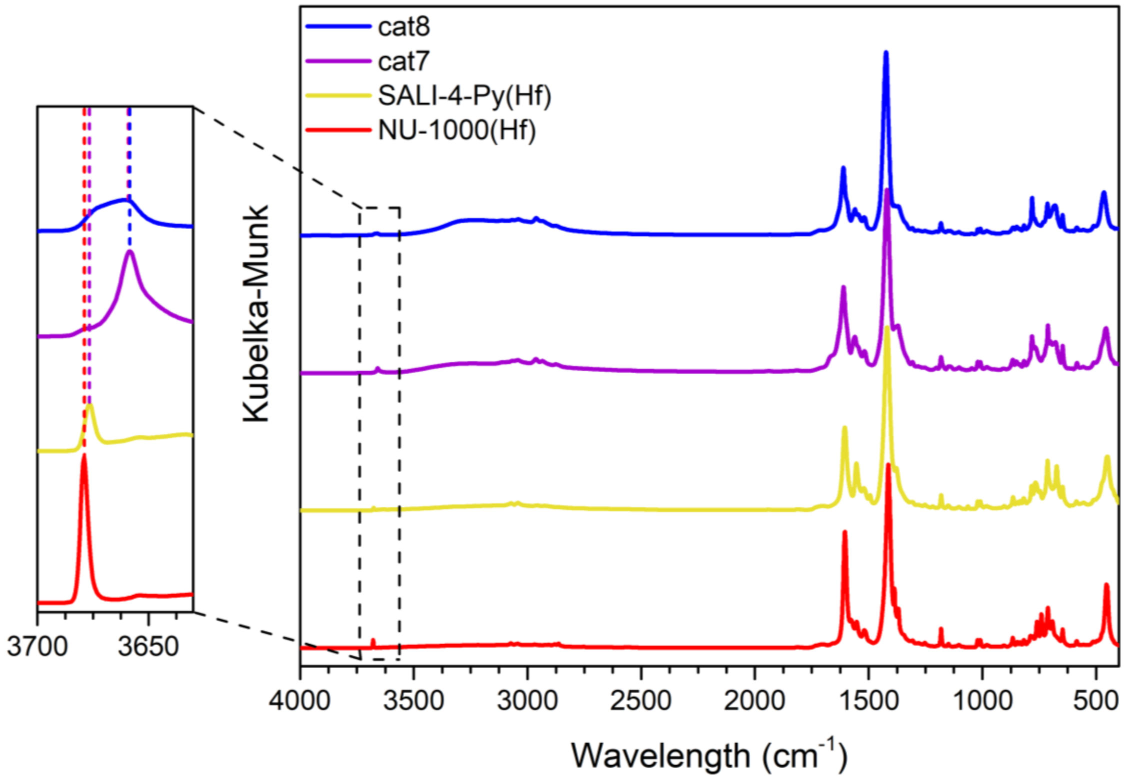

Figure S58. DRIFT spectra of Hf-based MOFs: **NU-1000(Hf)**, **SALI-4-Py(Hf)**, **cat7** and **cat8**.

S4.9. VT-DRIFTS

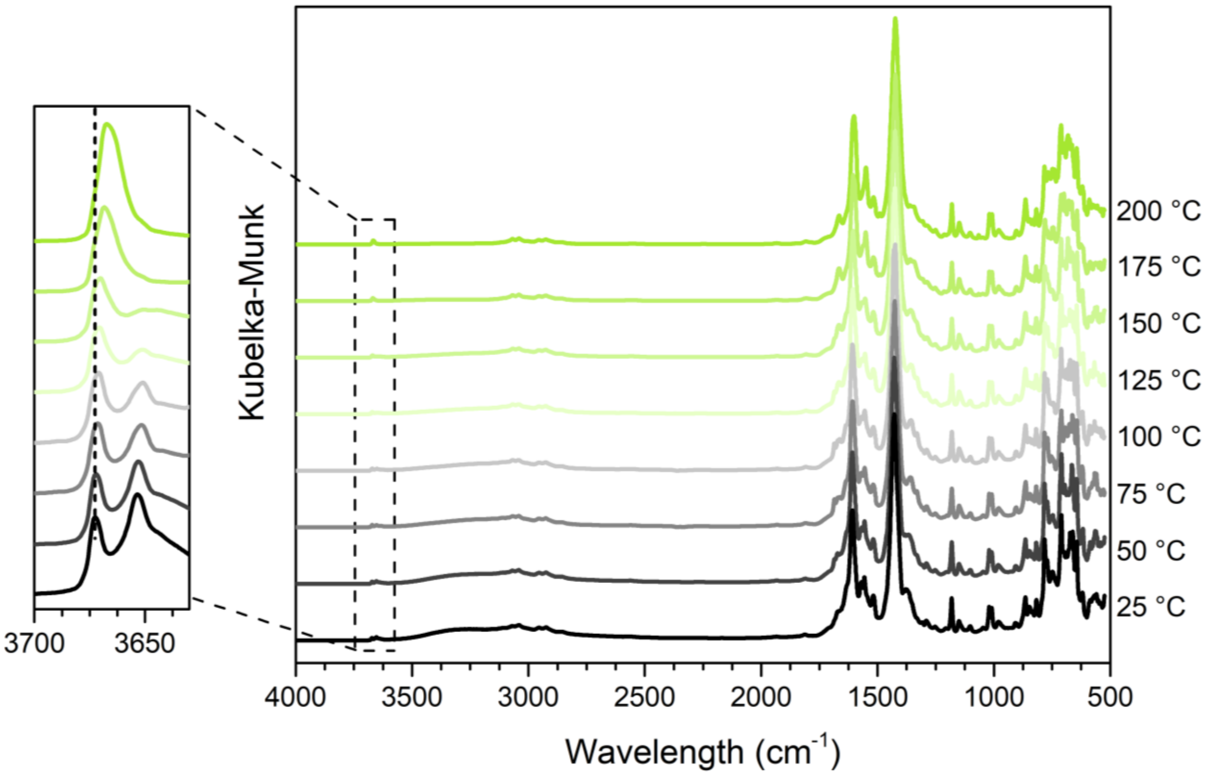

Figure S59. Variable-temperature DRIFT spectra of **cat1**.

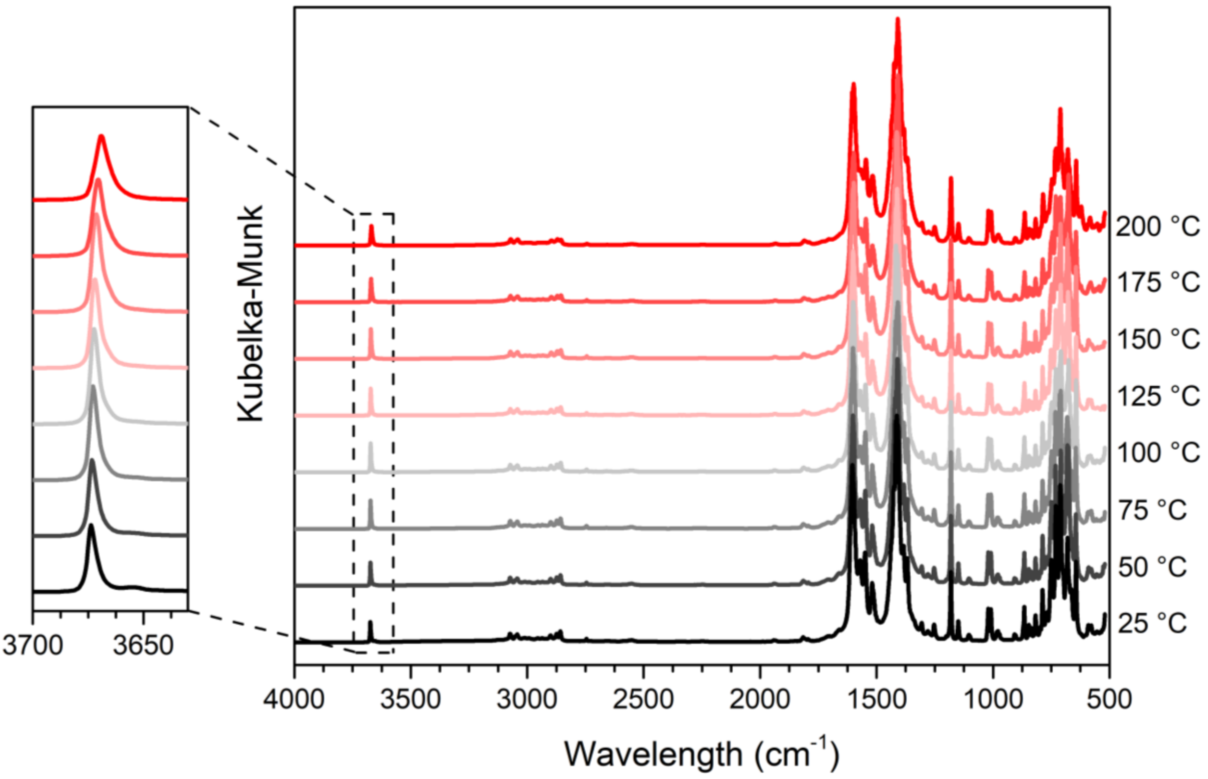

Figure S60. Variable-temperature DRIFT spectra of **NU-1000(Zr)**.

S5. Studies on catalytic performance of prepared *2-in-1* catalysts

S5.1. Probing cat1 as a model catalyst for cycloaddition of CO_2 to epoxides

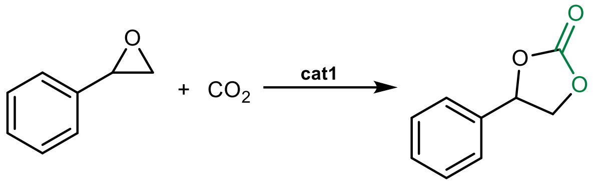

Figure S61. General scheme of synthesis of styrene carbonate by cycloaddition of carbon dioxide to styrene oxide catalyzed by **cat1** material.

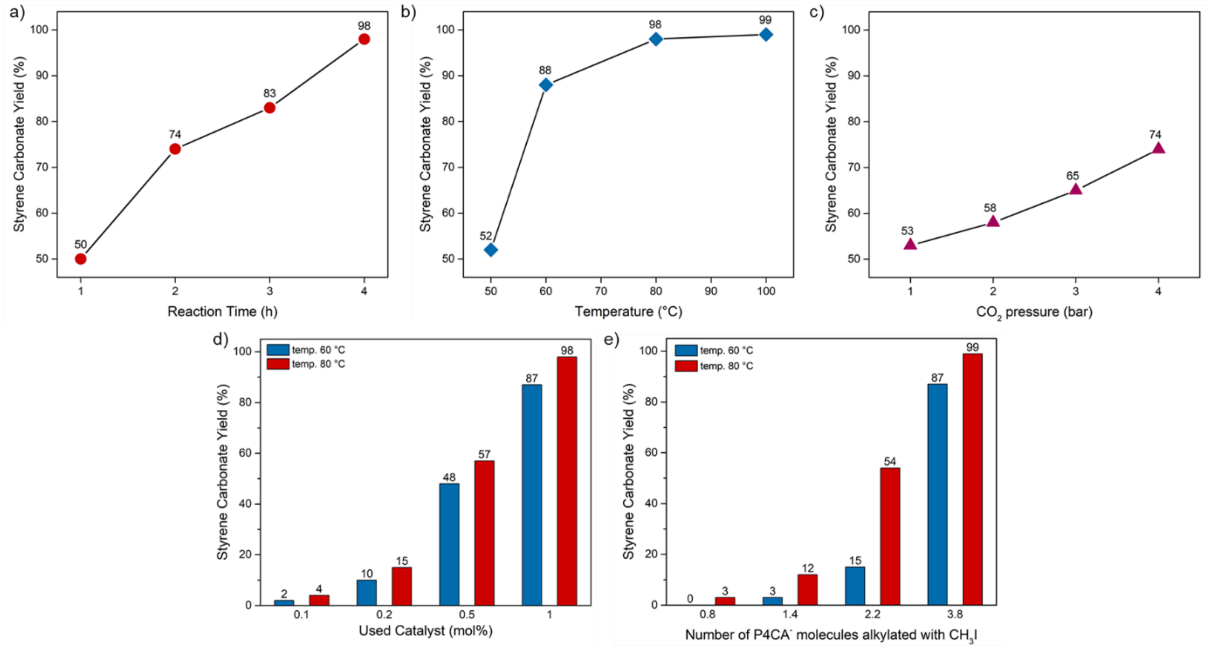

Figure S62. a) Yield versus time plot for cycloaddition of CO_2 to styrene oxide catalyzed by 1 mol% of **cat1** at 80 °C. b) Temperature influence on styrene carbonate yield investigated in reactions with 1 mol% of **cat1** for 4 h. c) The effect of the CO_2 amount on the styrene carbonate yield tested in reactions of 1 mol% of **cat1** at 80 °C for 2 h. d) Influence of the amount of **cat1** catalyst on styrene carbonate yield, reactions performed at 60 °C and 80 °C for 4 h. e) Influence of the amount of methylated pyridine group in **cat1** material on styrene carbonate yield, reactions performed at 60 and 80 °C for 4 h.

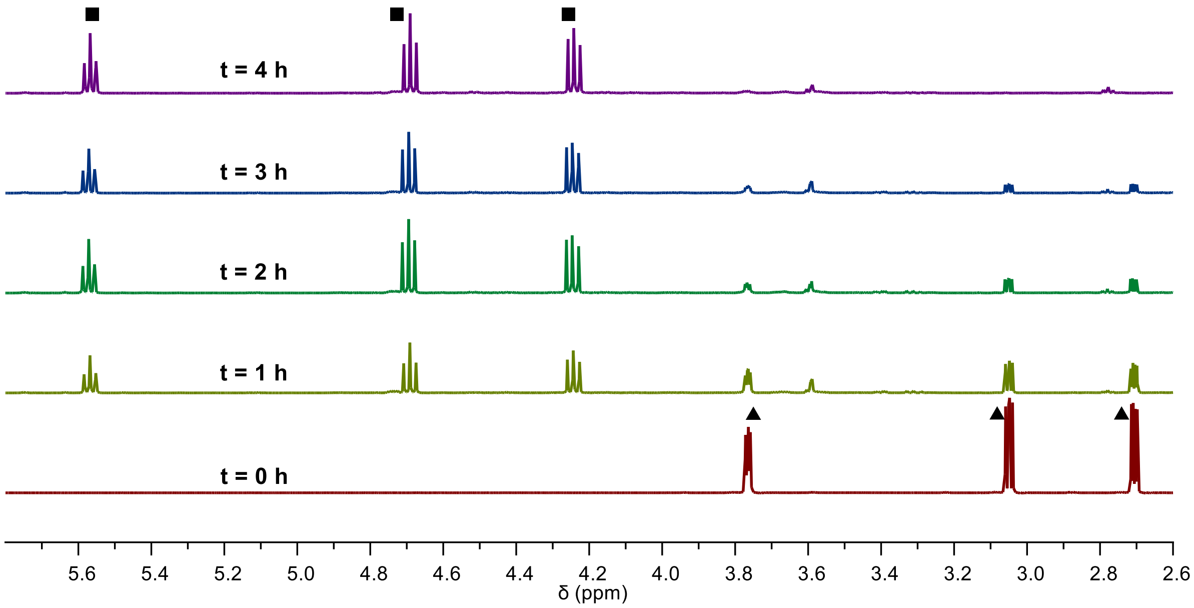

Figure S63. The ^{1}H NMR spectra of crude mixtures extracted after reaction of CO_2 with styrene oxide catalyzed by **cat1** material at 80 °C, using 1 mol% of obtained catalyst; ▲ denotates styrene oxide, ■ - styrene carbonate.

Table S3. Catalytic comparison of MOF-based catalyst used in CO_2 cycloaddition reaction with styrene oxide or butylene oxide.

MOF-based catalyst	Lewis acidic site	cocatalyst	solvent	substrate	CO_2 pressure (bar)	temperat ure (°C)	time (h)	cyclic carbonate yield (%)	ref
MOF-5	Zn	TBAB	–	styrene oxide	1	50	15	92	[10]
HbMOF1	Zn	TBAB	–	styrene oxide	1	30	24	78	[11]
Zn(Bmic)(AT)	Zn	TBAB	–	styrene oxide	5	90	6	78	[12]
PNU-21	Zn	TBAB	–	styrene oxide	4	80	8	66	[13]
PNU-22	Cd	TBAB	–	styrene oxide	4	80	8	34	[13]
Ni-TCPE1	Ni	TBAB	–	styrene oxide	10	100	12	99	[14]
$[Mn_4(L)_2(H_2O)_4]$	Mn	TBAB	–	styrene oxide	1	80	8	98	[15]
HP-MIL-88-NH_2(Fe)	Fe	TBAB	–	styrene oxide	1	45	12	40	[16]
$[Cu\text{-}(MTABA)(H_2O)]$	Cu	TBAB	–	styrene oxide	1	r.t.	24	68	[17]
PCN-222(Co)	Zr, Co	TBAB	–	styrene oxide	1	40	18	99	[18]
MOF-892	Zr	TBAB	–	styrene oxide	1	80	16	82	[19]
MOF-893	Zr	TBAB	–	styrene oxide	1	80	16	63	[19]
JLU-MOF58	Zr	TBAB	–	styrene oxide	1	80	24	95	[20]
NU-903	Zr	TBAB	CH_3CN	styrene oxide	1	r.t.	24	20	[21]
NU-904	Zr	TBAB	CH_3CN	styrene oxide	1	r.t.	24	23	[21]
NU-1008	Zr	TBAB	CH_3CN	styrene oxide	1	r.t.	24	99	[21]
Zr-NU-1008	Zr	TBAB	CH_3CN	styrene oxide	1	r.t.	20	89	[22]
Hf-NU-1000	Hf	TBAB	–	styrene oxide	1	r.t.	56	100	[23]
Hf-NU-1008	Hf	TBAB	CH_3CN	styrene oxide	1	r.t.	20	16	[22]
Ce-NU-1008	Ce	TBAB	CH_3CN	styrene oxide	1	r.t.	20	95	[22]
Th-NU-1008	Th	TBAB	CH_3CN	styrene oxide	1	r.t.	20	10	[22]
polyILs@MIL-101	Cr	–	CH_3CN	styrene oxide	1	70	48	81	[24]
FJI-C10	Cr	–	–	styrene oxide	1	80	48	48	[25]
MIL-IMAc-Br^-	Cr	–	–	styrene oxide	5	60	24	89.1	[26]
Mg-MOF-74	Mg	–	–	styrene oxide	20	100	1	50	[27]
ZnTCPP⊂(Br^-)Etim-UiO-66	Zn, Zr	–	–	styrene oxide	1	140	14	52.8	[28]
(I^-)Meim-UiO-66	Zr	–	–	styrene oxide	1	120	24	46	[29]
66Pym-MeI	Zr	–	–	styrene oxide	5	100	24	no reaction	[30]
67BPym-MeI	Zr	–	–	styrene oxide	5	100	24	4.8	[30]
**cat1**	Zr	–	–	styrene oxide	4	80	4	98	**this work**
**cat1**	Zr	–	–	styrene oxide	1	25	48	81	**this work**
**cat1**	Zr	–	–	styrene oxide	3.4	25	36	92	**this work**
$[Mn_4(L)_2(H_2O)_4]$	Mn	TBAB	–	butylene oxide	1	80	8	99	[15]
Zn(Bmic)(AT)	Zn	TBAB	–	butylene oxide	5	80	6	75	[12]
$[Zn_5(\mu_3\text{–}OH)_2(DBTA)_2(H_2O)_4]$	Zn	TBAB	–	butylene oxide	1	70	15	95	[31]
$[Cu_4[(C_{57}H_{32}N_{12})(COO)_8]$	Cu	TBAB	–	butylene oxide	1	r.t.	48	83	[32]
polyILs@MIL-101	Cr	–	CH_3CN	butylene oxide	1	45	48	94	[24]
MIL-101-N(*n*-Bu)$_3$Br	Cr	–	–	butylene oxide	20	80	8	86	[33]
MeIm-MIL-101	Cr	–	–	butylene oxide	20	115	1.5	83	[34]
MIL-101-IP	Cr	–	–	butylene oxide	1	25	48	95	[35]
MIL-IMAc-Br^-	Cr	–	–	butylene oxide	5	60	24	42	[26]
66Pym-MeI	Zr	–	–	butylene oxide	5	100	24	88	[30]
67BPym-MeI	Zr	–	–	butylene oxide	5	100	24	75	[30]
**cat1**	Zr	–	–	butylene oxide	4	80	4	97	**this work**

where H_2Bmic - 1-benzimidazole-5-carboxylic acid; HAT - 5-aminotetrazole; H_4L - 2,6-di(2′,5′-dicarboxylphenyl)pyridine; H_2MTABA - 4,4′-((6-methoxy-1,3,5-triazine-2,4-diyl)bis(azanediyl))dibenzoic acid); H_4DBTA - 2,2′-dihydroxy-1,1′-binaphthyl-3,3′,6,6′-tetrakis(4-benzoic acid)

S5.2. *In situ* IR studies on interactions of CO_2 with cat1 and NU-1000(Zr)

The influence of the presence of CO_2 in the sample of **NU-1000(Zr)** and **cat1** (Fig. S64) were studies using DRIFT spectroscopy. The spectra were collected on Nicolet iS50 FT-IR Spectrometer (Thermo Scientific). The measured samples were diluted with KBr, grinded in a mortar and placed in a special holder of the Praying Mantis DRIFT accessory equipped with high temperature reaction chamber (Harrick Scientific Products Inc). The sample was heated under the nitrogen flow in a rate of 1 °C/min up to 80 °C and further incubated at that temperature for all of the performed measurements. The precise temperature control was performed using EZ-ZONE software. The DRIFT spectra were collected in the range of 4000-700 cm^{-1} at constant temperature of 80 °C.

The difference DRIFT spectra were measured using as a background a spectrum of the selected sample (**cat1** or **NU-1000(Zr)**) under the nitrogen at 80 °C. After that, the CO_2 (1 atm.) was introduced to the reaction chamber and the sample was purged twice with this gas in order to remove the residual nitrogen. Then the system was closed (t = 0 min) and the following spectra were collected at specified times (Fig. S64). In the obtained time-resolved DRIFT difference spectra of **cat1** and **NU-1000(Zr)** the regions of CO_2 vibrational bands were omitted for clarity. After the introduction of CO_2 to the measured samples, no significant changes in collected spectra were observed, similarly as observed for UiO-66 material.[36] We observed the appearance of a new band at 2077 cm^{-1} (both in **NU-1000(Zr)** and **cat1**) which does not change its intensity throughout the performed experiment. The clear appearance and disappearance of this band when CO_2 is present in the measured system (Fig. S65) suggest that this band corresponds to the physisorpted CO_2 molecules in MOF.

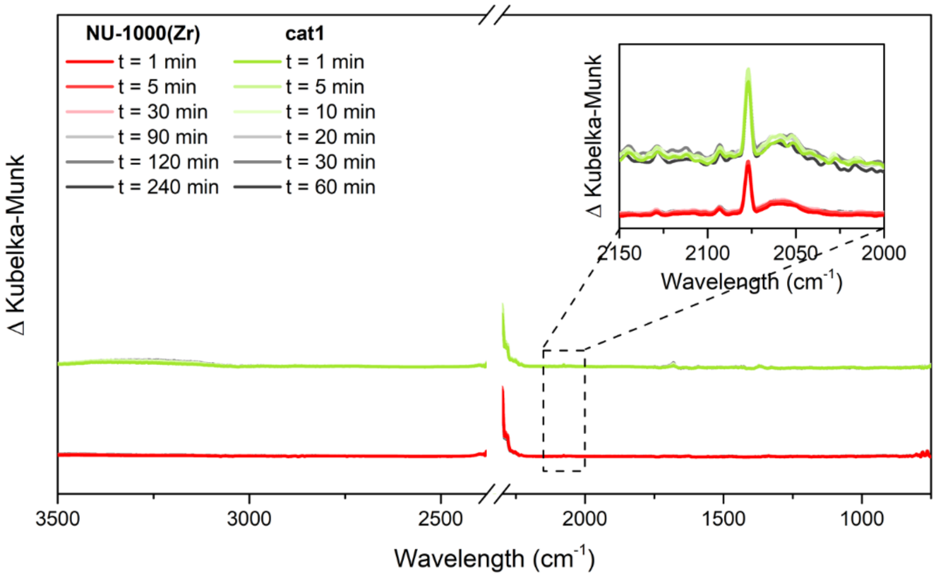

Figure S64. Time-resolved DRIFT difference spectra of **cat1** (green) and **NU-1000(Zr)** (red) under CO_2 (1 atm) at 80 °C. The spectrum of corresponding **cat1** or **NU-1000(Zr)** material at 80 °C under the N_2 was measured as a background for this study.

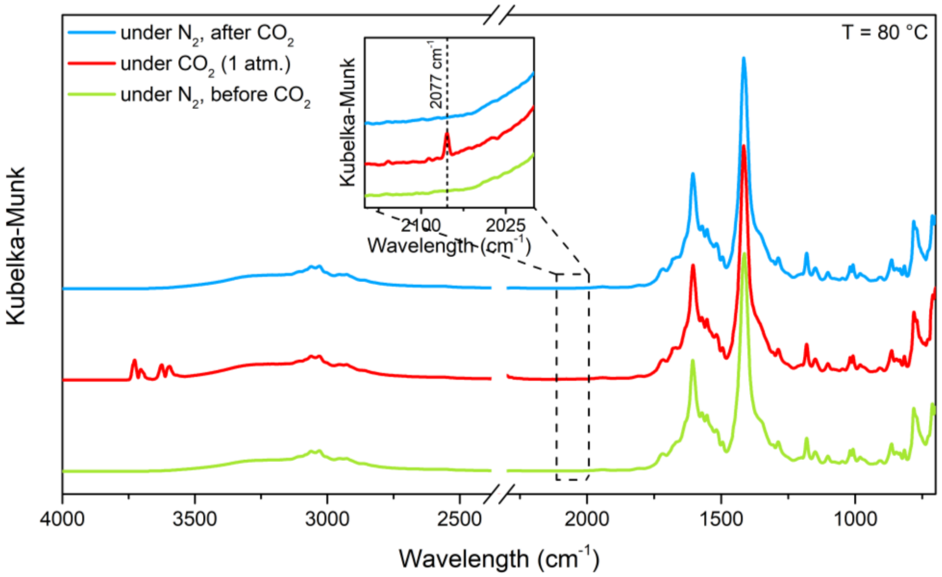

Figure S65. DRIFT spectra of **cat1** measured before and after exposure to CO_2 (1 atm) at 80 °C; (KBr was used as a background). The observed new band at 2077 cm^{-1} appear only when CO_2 is present in the system.

S5.3. The recyclability tests of cat1 in reaction of CO_2 with styrene oxide

The reactions of styrene oxide with CO_2 were carried out in the same optimized conditions (1 mol% of **cat1**, 80 °C, 4 h) without the addition of solvent or external co-catalyst and using the recovered catalyst from previous runs. Before each subsequent reaction, the remained solid (separated by centrifugation) was additionally washed three times with acetone and dried under vacuum. The yields of obtained cyclic styrene carbonate after each run are presented in Figure S66.

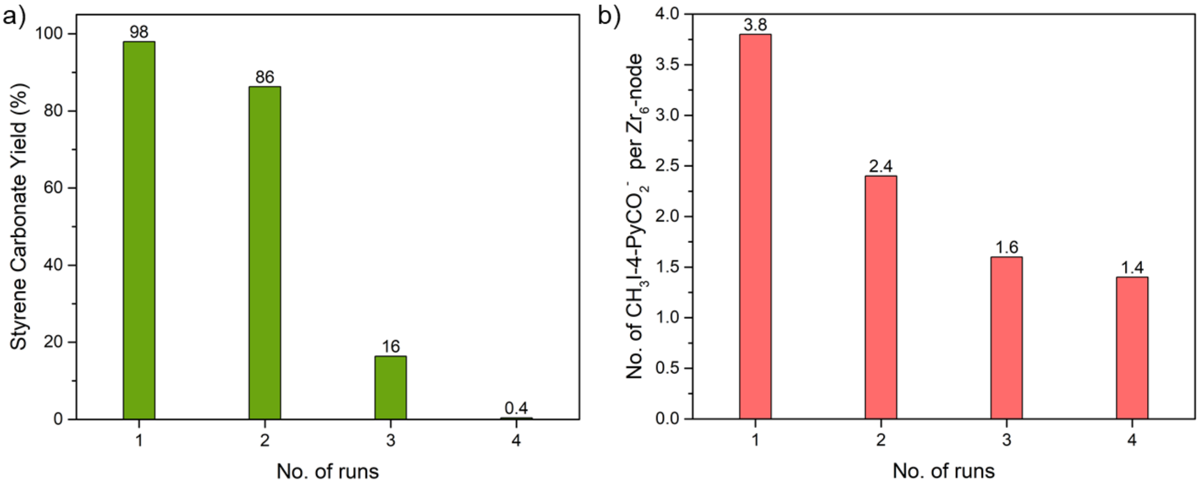

Figure S66. a) Test on recyclability of **cat1** in the reaction of styrene oxide and CO_2. Reaction conditions: 1 mol% of **cat1**, 80 °C, 4 h, without the addition of solvent or external co-catalyst. b) The number of CH_3I-4-$PyCO_2^-$ molecules per metal node estimated from ^{1}H NMR analysis of digested **cat1** samples (Fig. S68) used as the starting catalyst in the corresponding reactions in Fig. S66**S66**a.

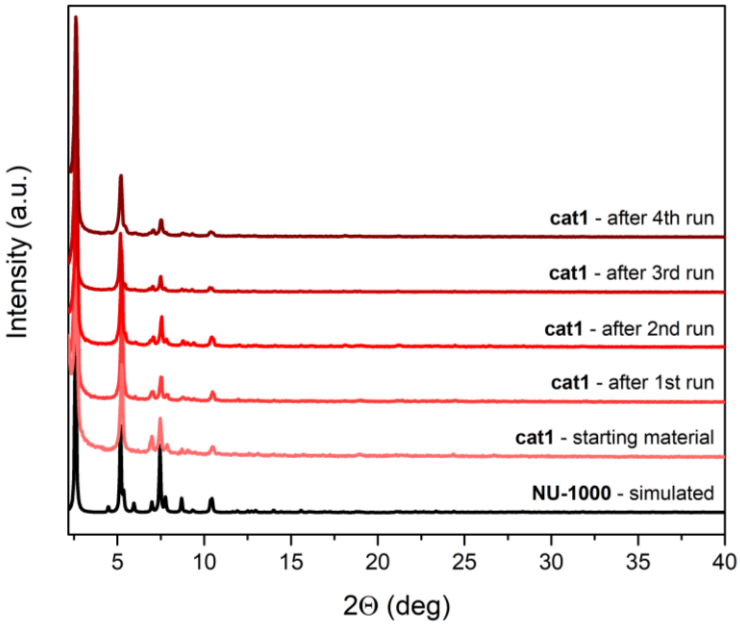

Figure S67. PXRD patterns of **cat1** before and after each run in the catalytic reaction of CO_2 with styrene oxide

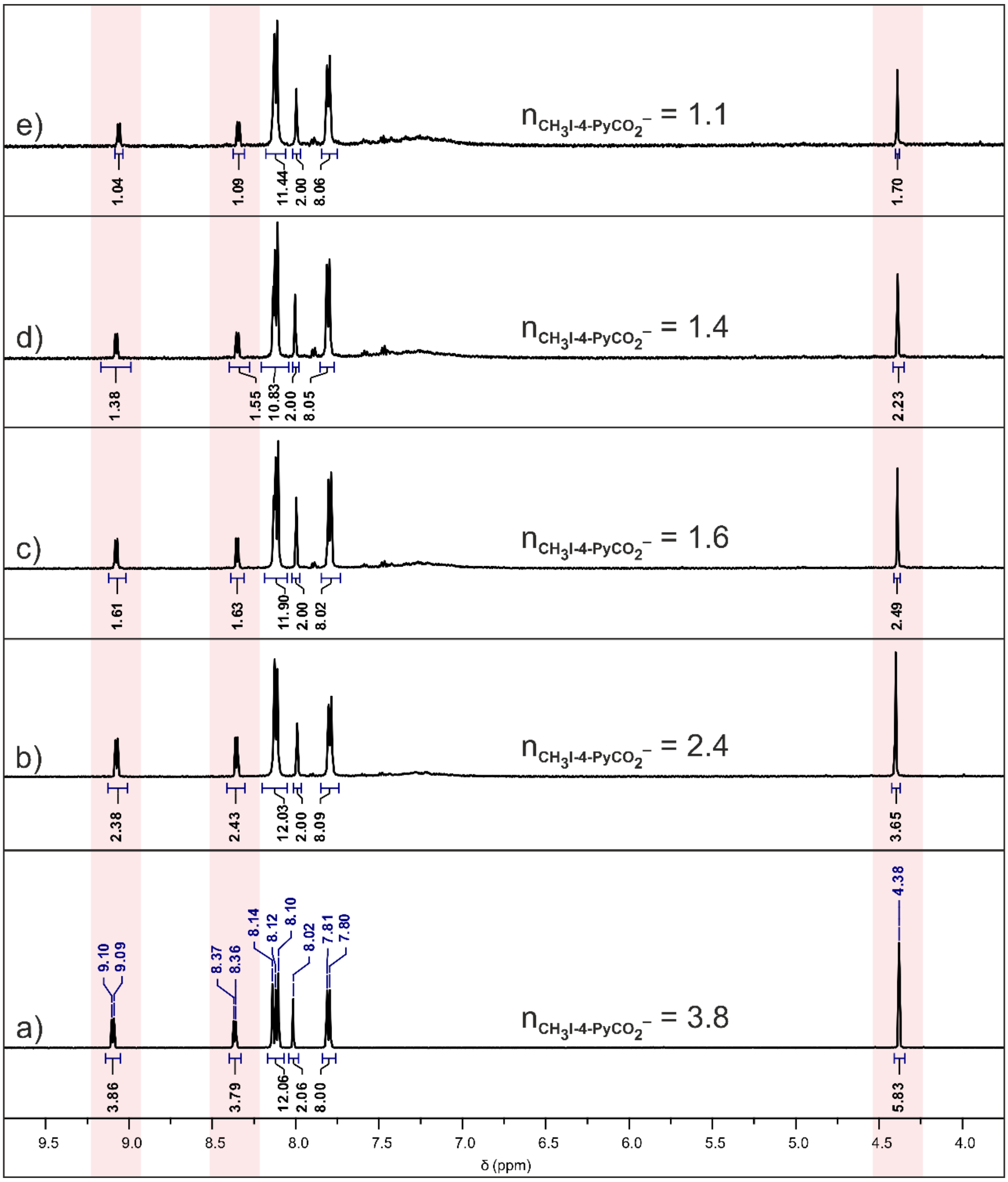

Figure S68. ^{1}H NMR spectrum of **cat1** used in a reaction of CO_2 and styrene oxide. a) before reaction, b) after 1st run, c) after 2nd run, d) after 3rd run, e) after 4th run. The number of CH_3I-4-$PyCO_2^-$ molecules was estimated by integrating proton signals of the ligand (annotated in red) against those of the $TBAPy^{4-}$ linker.

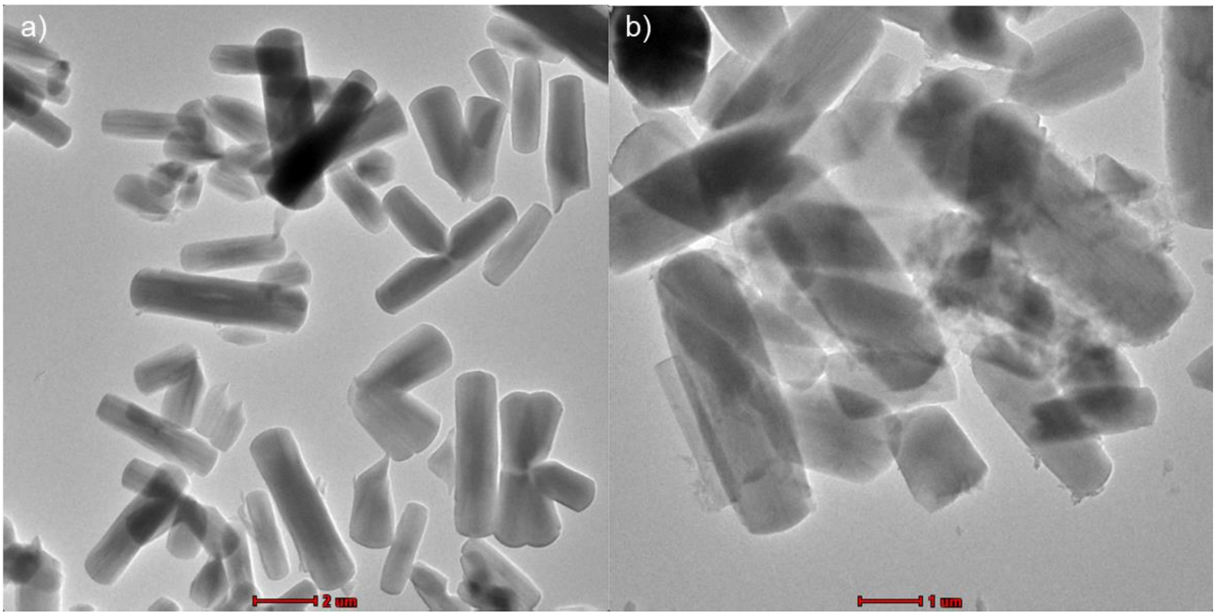

Figure S69. TEM images of **cat1** before (a) and after (b) catalytic reaction with CO_2 and styrene oxide at 80 °C for 4 h.

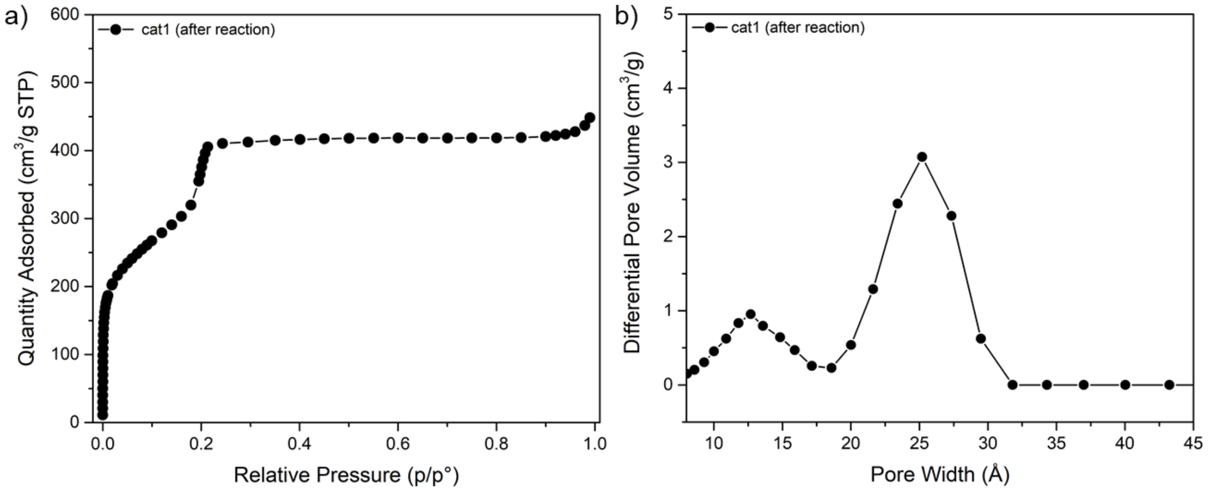

Figure S70. a) Nitrogen adsorption measured at 77 K in **cat1** collected after reaction of CO_2 with styrene oxide at 80 °C for 4 h. b) Density functional theory (DFT) pore size distribution (PSD) of **cat1** collected after catalytic reaction.

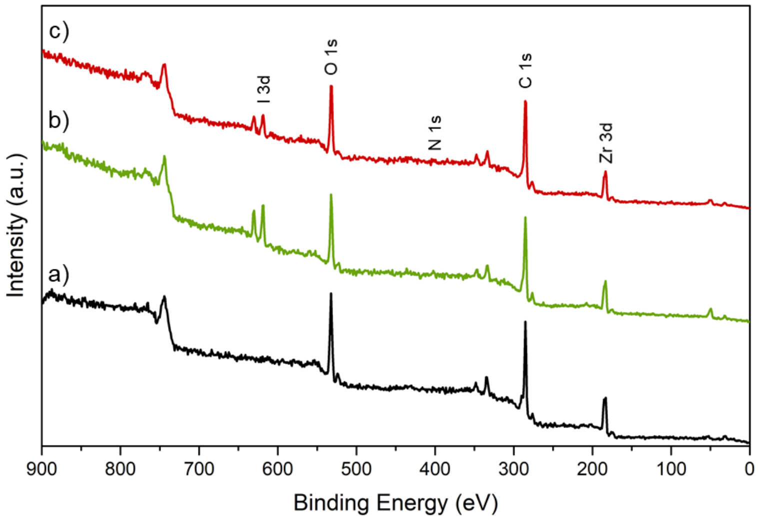

Figure S71. XPS survey of a) **NU-1000(Zr)** and **cat1** material before (b) and after (c) catalytic reaction of CO_2 with styrene oxide at 80 °C for 4 h.

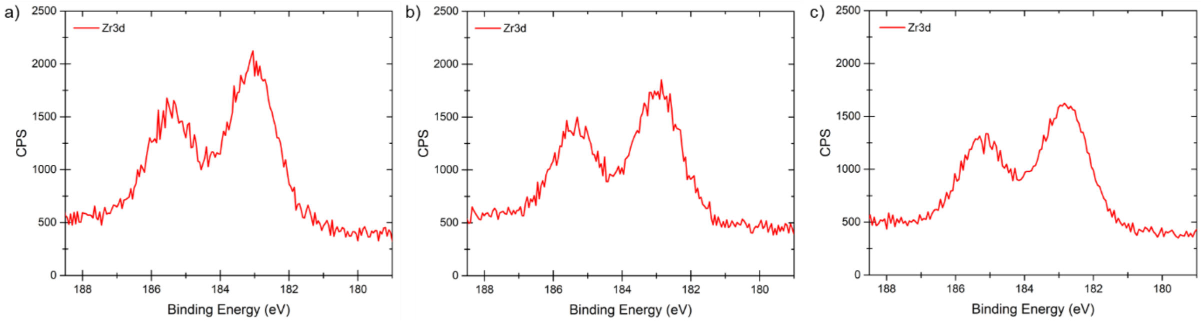

Figure S72. Zr 3d spectra of a) **NU-1000(Zr)** and **cat1** before (b) and after (c) catalytic reaction.

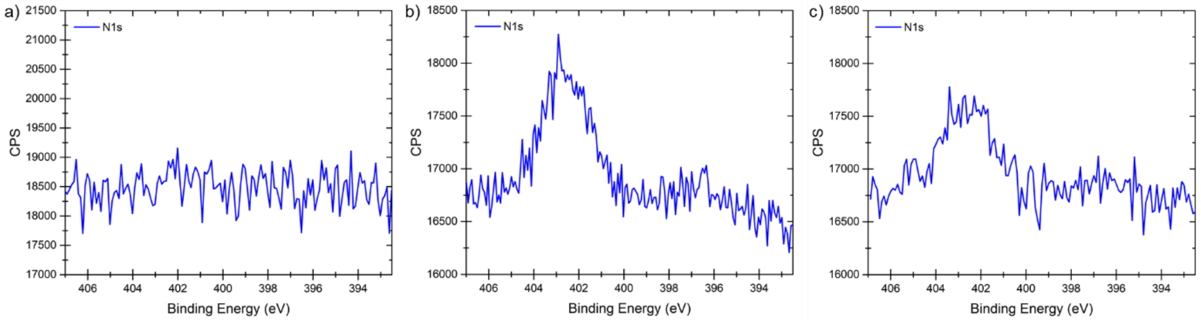

Figure S73. N 1s spectra of a) **NU-1000(Zr)** and **cat1** before (b) and after (c) catalytic reaction.

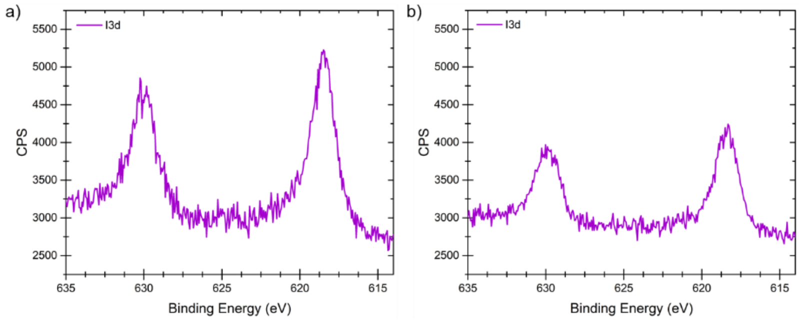

Figure S74. I 3d spectra of **cat1** before (a) and after (b) catalytic reaction.

Table S4. XPS analysis the I (a) and N (b) contents in **cat1** before and after reaction of CO_2 with styrene oxide.

a)

#	sample	Zr 3d	N 1s	Content of nitrogen in cat1	
		atom%[1]	atom%[1]	per Zr-node	%
1	**cat1**	4.39	1.16	1.58	100
2	**cat1** (after reaction)	4.44	0.97	1.31	83

b)

#	sample	Zr 3d	I 3d 5/2	Content of iodide in cat1	
		atom%[1]	atom%[1]	per Zr-node	%
1	**cat1**	4.39	1.55	2.12	100
2	**cat1** (after reaction)	4.44	0.90	1.22	58

[1] based on the collected XPS data

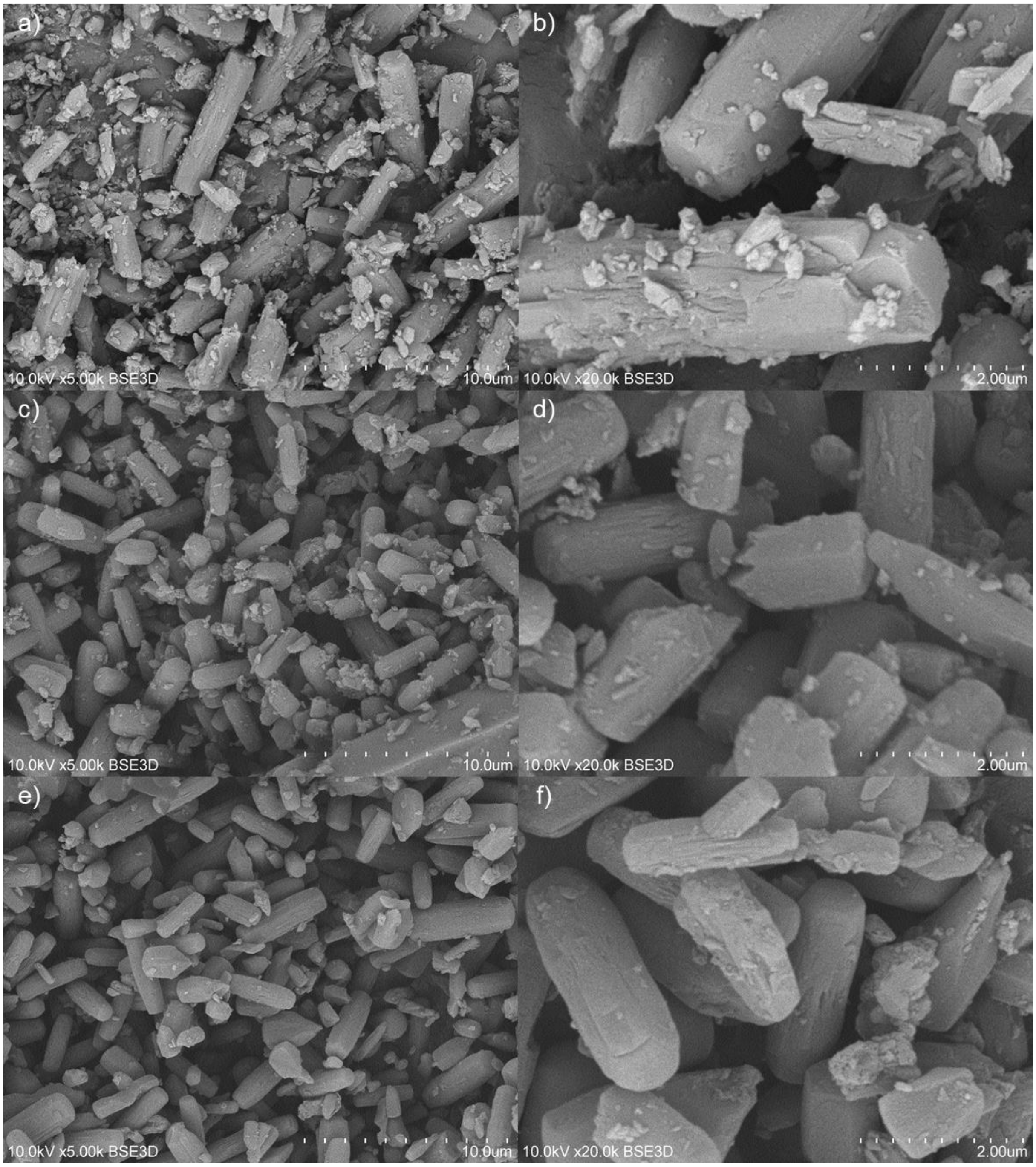

Figure S75. SEM images of **cat1** after reaction of CO_2 with styrene oxide at 80 °C for 4 h; sample collected after 1st (a-b), 2nd (c-d) and 3rd run (e-f).

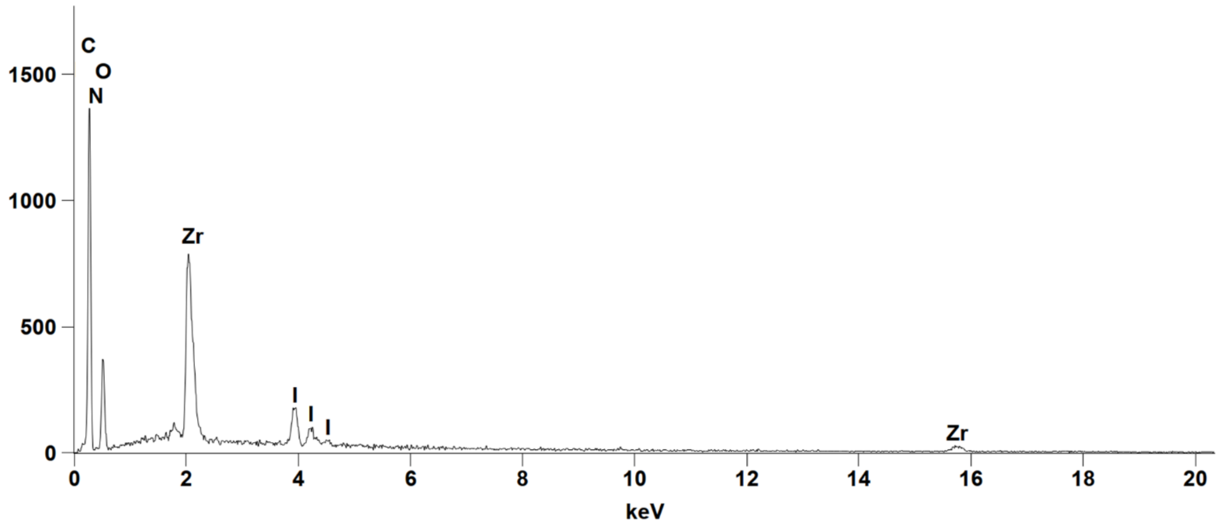

Figure S76. EDS spectrum of **cat1** collected after 1st run in reaction of CO_2 with styrene oxide.

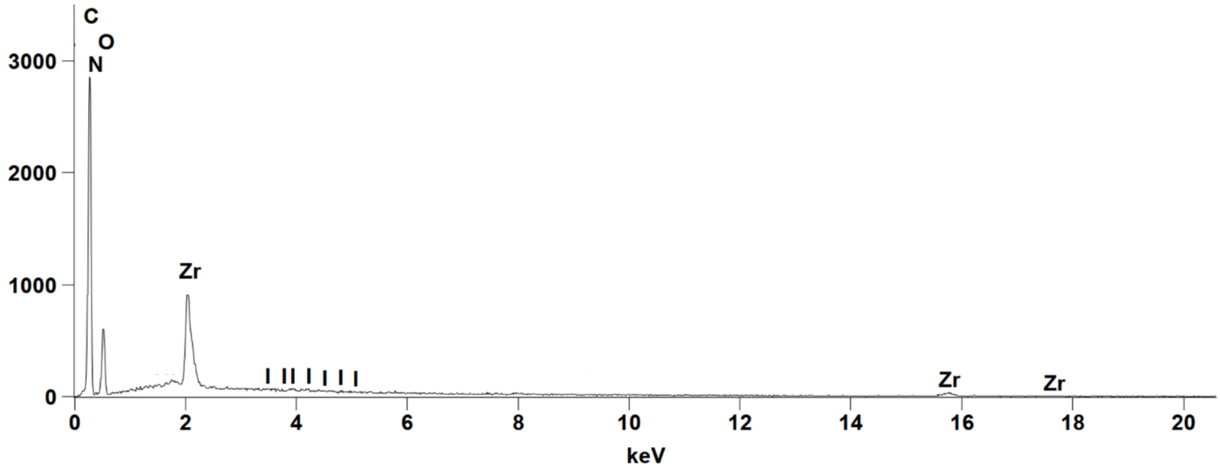

Figure S77. EDS spectrum of **cat1** collected after 2nd run in reaction of CO_2 with styrene oxide.

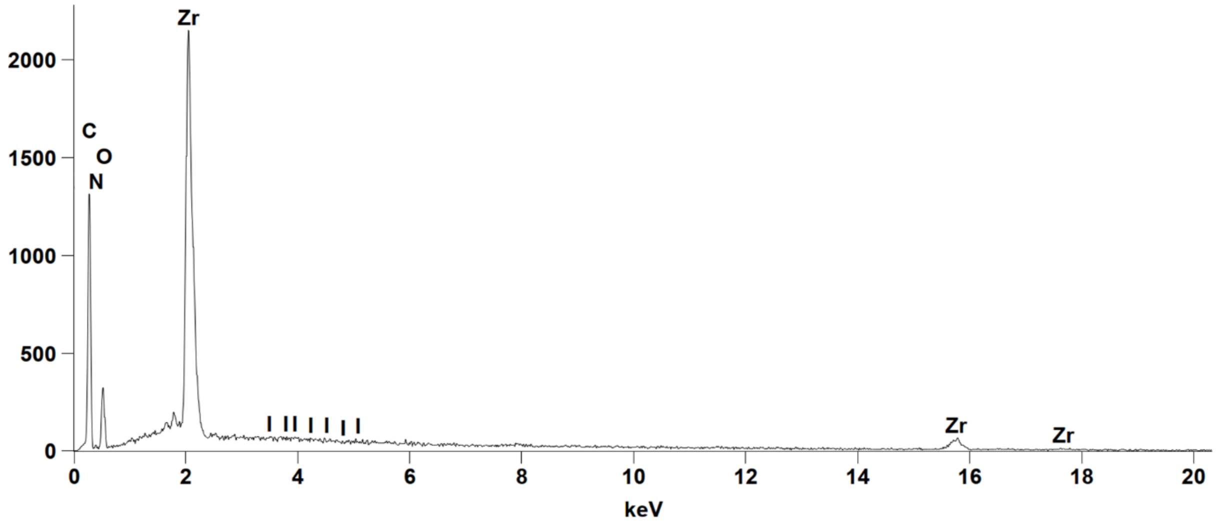

Figure S78. EDS spectrum of **cat1** collected after 3rd run in reaction of CO_2 with styrene oxide.

Table S5. EDS analysis of Zr and I content in **cat1** materials used in in the reaction of CO_2 with styrene oxide.

#	sample	Zr	I	Content of iodide in cat1	
		atom%[1]	atom%[1]	per Zr-node	%
1	**cat1**	1.51	1	3.97	100
2	**cat1** (after 1st run)	1.92	0.84	2.63	66
3	**cat1** (after 2nd run)	1.69	0.05	0.18	4
4	**cat1** (after 3rd run)	2.89	0.02	0.04	1

[1] based on data collected in EDS analysis

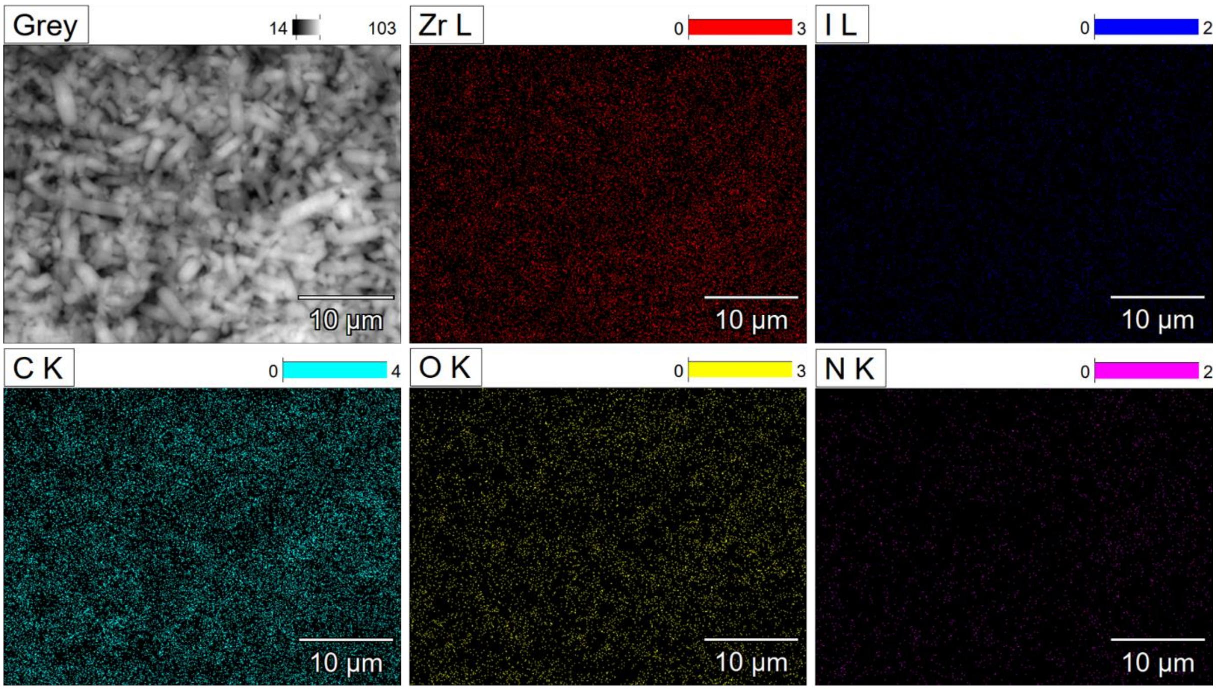

Figure S79. EDS mapping of **cat1** after reaction of CO_2 with styrene oxide at 80 °C for 4 h (1st run).

S5.4. Re-insertion of CH_3I-4-$PyCO_2^-$ in cat1 (after catalysis)

The **cat1** material was collected after catalytic reaction of CO_2 with styrene oxide (1 mol% of **cat1**, 80 °C, 4 h). The material was washed several times with acetone and dried under vacuum. Sample for the ^{1}H NMR analysis was then taken (**Fig. S80a**), and the rest of the solid was subjected the two-step procedure of Zr-node functionalization described in the Experimental Section. Namely, the collected solid was soaked in the 0.03 M solution of pyridine-4-carboxylic acid (4-PyCOOH) in ethanol at 60 °C for 24 h, which was followed by the reaction with methyl iodide in acetonitrile for 60 °C for 24 h. The obtained solid was washed with acetonitrile and dried under vacuum. The obtained material was analyzed with ^{1}H NMR spectroscopy (**Fig. S80b**) showing complete regeneration of the **cat1** material.

The regenerated **cat1** was then tested in the reaction of CO_2 with styrene oxide in the same optimized conditions (1 mol% of **cat1**, 80 °C, 4 h) without the addition of solvent or external co-catalyst. The yield

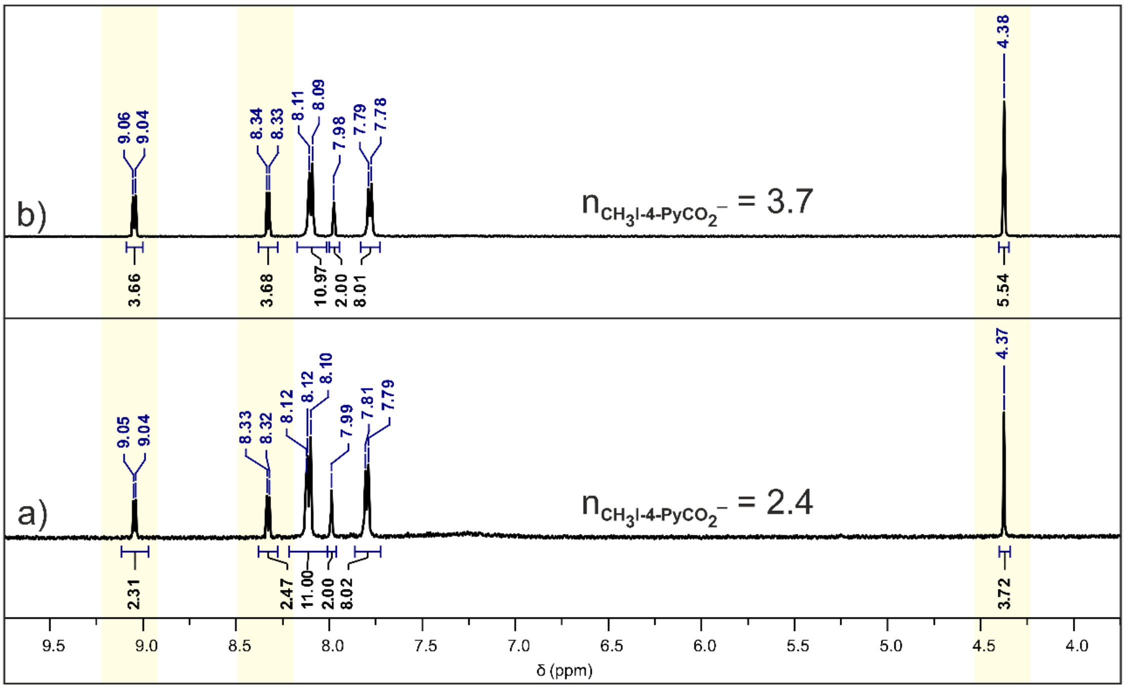

Figure S80. ^{1}H NMR spectrum of **cat1** before (a) and after (b) re-modification procedure leading to reintroduction of CH_3I-4-$PyCO_2^-$ ligands. The samples were digested in the D_2SO_4/DMSO-d_6 mixture; the number of CH_3I-4-$PyCO_2^-$ molecules was estimated by integrating proton signals of the ligand (annotated in yellow) against those of the $TBAPy^{4-}$ linker.

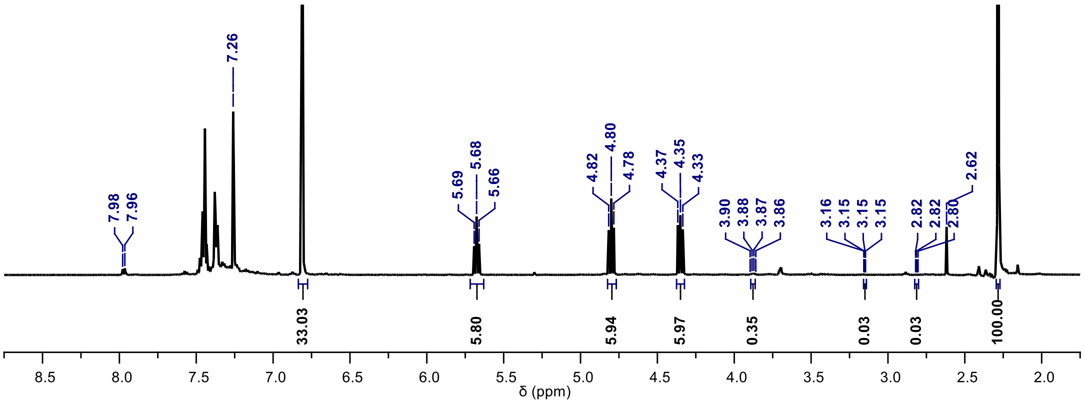

Figure S81. ^{1}H NMR of crude mixture in $CDCl_3$ collected after reaction of CO_2 with styrene oxide in the presence of re-modified **cat1** material (**Fig. S80b**). The mesitylene was used as an internal standard; the calculated yield of cyclic styrene carbonate was 98%. The doublet at 7.97 ppm and singlet at 2.62 ppm were assigned to the 2 aromatic protons and 3 protons of methyl group, respectively, confirming leakage of the CH_3I-4-$PyCO_2^-$ ligand from the catalyst.

S6. References

(1) Mondloch, J. E.; Bury, W.; Fairen-Jimenez, D.; Kwon, S.; Demarco, E. J.; Weston, M. H.; Sarjeant, A. A.; Nguyen, S. T.; Stair, P. C.; Snurr, R. Q.; Farha, O. K.; Hupp, J. T. Vapor-Phase Metalation by Atomic Layer Deposition in a Metal-Organic Framework. *Journal of the American Chemical Society* **2013**, *135* (28), 10294–10297. https://doi.org/10.1021/ja4050828.

(2) Islamoglu, T.; Otake, K.; Li, P.; Buru, C. T.; Peters, A. W.; Akpinar, I.; Garibay, S. J.; Farha, O. K. Revisiting the Structural Homogeneity of NU-1000, a Zr-Based Metal–Organic Framework. *CrystEngComm* **2018**, *20* (39), 5913–5918. https://doi.org/10.1039/c8ce00455b.

(3) Beyzavi, M. H.; Klet, R. C.; Tussupbayev, S.; Borycz, J.; Vermeulen, N. A.; Cramer, C. J.; Stoddart, J. F.; Hupp, J. T.; Farha, O. K. A Hafnium-Based Metal–Organic Framework as an Efficient and Multifunctional Catalyst for Facile CO_2 Fixation and Regioselective and Enantioretentive Epoxide Activation. *J. Am. Chem. Soc.* **2014**, *136* (45), 15861–15864. https://doi.org/10.1021/ja508626n.

(4) Deria, P.; Bury, W.; Hupp, J. T.; Farha, O. K. Versatile Functionalization of the NU-1000 Platform by Solvent-Assisted Ligand Incorporation. *Chem. Commun.* **2014**, *50* (16), 1965. https://doi.org/10.1039/c3cc48562e.

(5) Pander, M.; Żelichowska, A.; Bury, W. Probing Mesoporous Zr-MOF as Drug Delivery System for Carboxylate Functionalized Molecules. *Polyhedron* **2018**, *156*, 131–137. https://doi.org/10.1016/j.poly.2018.09.006.

(6) Rouquerol, J.; Llewellyn, P.; Rouquerol, F. Is the BET Equation Applicable to Microporous Adsorbents? In *Studies in surface science and catalysis*; Elsevier B. V., 2007; Vol. 160, pp 49–56. https://doi.org/10.1016/S0167-2991(07)80008-5.

(7) Walton, K. S.; Snurr, R. Q. Applicability of the BET Method for Determining Surface Areas of Microporous Metal-Organic Frameworks. *Journal of the American Chemical Society* **2007**, *129* (27), 8552–8556. https://doi.org/10.1021/ja071174k.

(8) Mason, J. A.; Sumida, K.; Herm, Z. R.; Krishna, R.; Long, J. R. Evaluating Metal–Organic Frameworks for Post-Combustion Carbon Dioxide Capture via Temperature Swing Adsorption. *Energy Environ. Sci.* **2011**, *4* (8), 3030–3040. https://doi.org/10.1039/C1EE01720A.

(9) Shearer, G. C.; Chavan, S.; Bordiga, S.; Svelle, S.; Olsbye, U.; Lillerud, K. P. Defect Engineering: Tuning the Porosity and Composition of the Metal–Organic Framework UiO-66 via Modulated Synthesis. *Chem. Mater.* **2016**, *28* (11), 3749–3761. https://doi.org/10.1021/acs.chemmater.6b00602.

(10) Song, J.; Zhang, Z.; Hu, S.; Wu, T.; Jiang, T.; Han, B. MOF-5/n-Bu4NBr: An Efficient Catalyst System for the Synthesis of Cyclic Carbonates from Epoxides and CO2 under Mild Conditions. *Green Chem.* **2009**, *11* (7), 1031. https://doi.org/10.1039/b902550b.

(11) Das, R.; Muthukumar, D.; Pillai, R. S.; Nagaraja, C. M. Rational Design of a Zn(II)-MOF with Multiple Functional Sites for Highly Efficient Fixation of CO2 at Mild Conditions: A Combined Experimental and Theoretical Investigation. *Chemistry – A European Journal* **2020**, *n/a* (n/a). https://doi.org/10.1002/chem.202002688.

(12) Li, Y.; Zhang, X.; Lan, J.; Xu, P.; Sun, J. Porous Zn(Bmic)(AT) MOF with Abundant Amino Groups and Open Metal Sites for Efficient Capture and Transformation of CO_2. *Inorg. Chem.* **2019**, *58* (20), 13917–13926. https://doi.org/10.1021/acs.inorgchem.9b01762.

(13) Rachuri, Y.; Kurisingal, J. F.; Chitumalla, R. K.; Vuppala, S.; Gu, Y.; Jang, J.; Choe, Y.; Suresh, E.; Park, D.-W. Adenine-Based Zn(II)/Cd(II) Metal–Organic Frameworks as Efficient Heterogeneous Catalysts for Facile CO_2 Fixation into Cyclic Carbonates: A DFT-Supported Study of the Reaction Mechanism. *Inorg. Chem.* **2019**, *58* (17), 11389–11403. https://doi.org/10.1021/acs.inorgchem.9b00814.

(14) Zhou, Z.; He, C.; Xiu, J.; Yang, L.; Duan, C. Metal–Organic Polymers Containing Discrete Single-Walled Nanotube as a Heterogeneous Catalyst for the Cycloaddition of

Carbon Dioxide to Epoxides. *J. Am. Chem. Soc.* **2015**, *137* (48), 15066–15069. https://doi.org/10.1021/jacs.5b07925.
(15) Cheng, S.; Wu, Y.; Jin, J.; Liu, J.; Wu, D.; Yang, G.; Wang, Y.-Y. New Multifunctional 3D Porous Metal–Organic Framework with Selective Gas Adsorption, Efficient Chemical Fixation of CO_2 and Dye Adsorption. *Dalton Trans.* **2019**, *48* (22), 7612–7618. https://doi.org/10.1039/C9DT01249D.
(16) Kurisingal, J. F.; Rachuri, Y.; Gu, Y.; Choe, Y.; Park, D.-W. Fabrication of Hierarchically Porous MIL-88-NH_2 (Fe): A Highly Efficient Catalyst for the Chemical Fixation of CO_2 under Ambient Pressure. *Inorg. Chem. Front.* **2019**, *6* (12), 3613–3620. https://doi.org/10.1039/C9QI01163C.
(17) Das, P.; Mandal, S. K. Unprecedented High Temperature CO2 Selectivity and Effective Chemical Fixation by a Copper-Based Undulated Metal–Organic Framework. *ACS Appl. Mater. Interfaces* **2020**. https://doi.org/10.1021/acsami.0c09024.
(18) Carrasco, S.; Sanz-Marco, A.; Martín-Matute, B. Fast and Robust Synthesis of Metalated PCN-222 and Their Catalytic Performance in Cycloaddition Reactions with CO_2. *Organometallics* **2019**, *38* (18), 3429–3435. https://doi.org/10.1021/acs.organomet.9b00273.
(19) Nguyen, P. T. K.; Nguyen, H. T. D.; Nguyen, H. N.; Trickett, C. A.; Ton, Q. T.; Gutiérrez-Puebla, E.; Monge, M. A.; Cordova, K. E.; Gándara, F. New Metal–Organic Frameworks for Chemical Fixation of CO_2. *ACS Appl. Mater. Interfaces* **2018**, *10* (1), 733–744. https://doi.org/10.1021/acsami.7b16163.
(20) Sun, X.; Gu, J.; Yuan, Y.; Yu, C.; Li, J.; Shan, H.; Li, G.; Liu, Y. A Stable Mesoporous Zr-Based Metal Organic Framework for Highly Efficient CO_2 Conversion. *Inorg. Chem.* **2019**, *58* (11), 7480–7487. https://doi.org/10.1021/acs.inorgchem.9b00701.
(21) Lyu, J.; Zhang, X.; Otake, K.; Wang, X.; Li, P.; Li, Z.; Chen, Z.; Zhang, Y.; Wasson, M. C.; Yang, Y.; Bai, P.; Guo, X.; Islamoglu, T.; Farha, O. K. Topology and Porosity Control of Metal–Organic Frameworks through Linker Functionalization. *Chem. Sci.* **2019**, *10* (4), 1186–1192. https://doi.org/10.1039/C8SC04220A.
(22) Lyu, J.; Zhang, X.; Li, P.; Wang, X.; Buru, C. T.; Bai, P.; Guo, X.; Farha, O. K. Exploring the Role of Hexanuclear Clusters as Lewis Acidic Sites in Isostructural Metal–Organic Frameworks. *Chem. Mater.* **2019**, *31* (11), 4166–4172. https://doi.org/10.1021/acs.chemmater.9b00960.
(23) Beyzavi, M. H.; Klet, R. C.; Tussupbayev, S.; Borycz, J.; Vermeulen, N. A.; Cramer, C. J.; Stoddart, J. F.; Hupp, J. T.; Farha, O. K. A Hafnium-Based Metal–Organic Framework as an Efficient and Multifunctional Catalyst for Facile CO_2 Fixation and Regioselective and Enantioretentive Epoxide Activation. *J. Am. Chem. Soc.* **2014**, *136* (45), 15861–15864. https://doi.org/10.1021/ja508626n.
(24) Ding, M.; Jiang, H.-L. Incorporation of Imidazolium-Based Poly(Ionic Liquid)s into a Metal–Organic Framework for CO_2 Capture and Conversion. *ACS Catal.* **2018**, *8* (4), 3194–3201. https://doi.org/10.1021/acscatal.7b03404.
(25) Liang, J.; Xie, Y.-Q.; Wang, X.-S.; Wang, Q.; Liu, T.-T.; Huang, Y.-B.; Cao, R. An Imidazolium-Functionalized Mesoporous Cationic Metal–Organic Framework for Cooperative CO_2 Fixation into Cyclic Carbonate. *Chem. Commun.* **2018**, *54* (4), 342–345. https://doi.org/10.1039/C7CC08630J.
(26) Ma, D.; Zhang, Y.; Jiao, S.; Li, J.; Liu, K.; Shi, Z. A Tri-Functional Metal–Organic Framework Heterogeneous Catalyst for Efficient Conversion of CO2 under Mild and Co-Catalyst Free Conditions. *Chem. Commun.* **2019**, *55* (95), 14347–14350. https://doi.org/10.1039/C9CC08236K.
(27) Yang, D.-A.; Cho, H.-Y.; Kim, J.; Yang, S.-T.; Ahn, W.-S. CO2 Capture and Conversion Using Mg-MOF-74 Prepared by a Sonochemical Method. *Energy Environ. Sci.* **2012**, *5* (4), 6465–6473. https://doi.org/10.1039/C1EE02234B.
(28) Liang, J.; Xie, Y.-Q.; Wu, Q.; Wang, X.-Y.; Liu, T.-T.; Li, H.-F.; Huang, Y.-B.; Cao, R. Zinc Porphyrin/Imidazolium Integrated Multivariate Zirconium Metal–Organic Frameworks for Transformation of CO2 into Cyclic Carbonates. *Inorg. Chem.* **2018**, *57* (5), 2584–2593. https://doi.org/10.1021/acs.inorgchem.7b02983.

(29) Liang, J.; Chen, R.-P.; Wang, X.-Y.; Liu, T.-T.; Wang, X.-S.; Huang, Y.-B.; Cao, R. Postsynthetic Ionization of an Imidazole-Containing Metal–Organic Framework for the Cycloaddition of Carbon Dioxide and Epoxides. *Chem. Sci.* **2017**, *8* (2), 1570–1575. https://doi.org/10.1039/C6SC04357G.

(30) Ji, H.; Naveen, K.; Lee, W.; Kim, T. S.; Kim, D.; Cho, D.-H. Pyridinium-Functionalized Ionic Metal–Organic Frameworks Designed as Bifunctional Catalysts for CO2 Fixation into Cyclic Carbonates. *ACS Appl. Mater. Interfaces* **2020**. https://doi.org/10.1021/acsami.0c05912.

(31) Zhu, Q.-Q.; Zhang, W.-W.; Zhang, H.-W.; Yuan, Y.; Yuan, R.; Sun, F.; He, H. A Double-Walled Porous Metal–Organic Framework as a Highly Efficient Catalyst for Chemical Fixation of CO_2 with Epoxides. *Inorg. Chem.* **2019**, *58* (22), 15637–15643. https://doi.org/10.1021/acs.inorgchem.9b02717.

(32) Li, P.-Z.; Wang, X.-J.; Liu, J.; Lim, J. S.; Zou, R.; Zhao, Y. A Triazole-Containing Metal–Organic Framework as a Highly Effective and Substrate Size-Dependent Catalyst for CO_2 Conversion. *J. Am. Chem. Soc.* **2016**, *138* (7), 2142–2145. https://doi.org/10.1021/jacs.5b13335.

(33) Ma, D.; Li, B.; Liu, K.; Zhang, X.; Zou, W.; Yang, Y.; Li, G.; Shi, Z.; Feng, S. Bifunctional MOF Heterogeneous Catalysts Based on the Synergy of Dual Functional Sites for Efficient Conversion of CO_2 under Mild and Co-Catalyst Free Conditions. *J. Mater. Chem. A* **2015**, *3* (46), 23136–23142. https://doi.org/10.1039/C5TA07026K.

(34) Webb, W. R.; Potter, M. E.; Stewart, D. J.; Elliott, S. J.; Sazio, P. J. A.; Zhang, Z.; Luo, H.-K.; Teng, J.; Zhang, L.; Ivaldi, C.; Miletto, I.; Gianotti, E.; Raja, R. The Significance of Metal Coordination in Imidazole-Functionalized Metal–Organic Frameworks for Carbon Dioxide Utilization. *Chemistry – A European Journal* **2020**, *26* (60), 13606–13610. https://doi.org/10.1002/chem.202001561.

(35) Aguila, B.; Sun, Q.; Wang, X.; O'Rourke, E.; Al-Enizi, A. M.; Nafady, A.; Ma, S. Lower Activation Energy for Catalytic Reactions through Host–Guest Cooperation within Metal–Organic Frameworks. *Angewandte Chemie International Edition* **2018**, *57* (32), 10107–10111. https://doi.org/10.1002/anie.201803081.

(36) Grissom, T. G.; Driscoll, D. M.; Troya, D.; Sapienza, N. S.; Usov, P. M.; Morris, A. J.; Morris, J. R. Molecular-Level Insight into CO_2 Adsorption on the Zirconium-Based Metal–Organic Framework, UiO-66: A Combined Spectroscopic and Computational Approach. *J. Phys. Chem. C* **2019**, *123* (22), 13731–13738. https://doi.org/10/ggvq27.
